# Structure and Biocatalytic Scope of Coclaurine *N*‐Methyltransferase

**DOI:** 10.1002/anie.201805060

**Published:** 2018-06-28

**Authors:** Matthew R. Bennett, Mark L. Thompson, Sarah A. Shepherd, Mark S. Dunstan, Abigail J. Herbert, Duncan R. M. Smith, Victoria A. Cronin, Binuraj R. K. Menon, Colin Levy, Jason Micklefield

**Affiliations:** ^1^ School of Chemistry Manchester Institute of Biotechnology The University of Manchester 131 Princess Street Manchester M1 7DN UK

**Keywords:** biocatalysis, biosynthesis, mechanism, Methyltransferase, structure

## Abstract

Benzylisoquinoline alkaloids (BIAs) are a structurally diverse family of plant secondary metabolites, which have been exploited to develop analgesics, antibiotics, antitumor agents, and other therapeutic agents. Biosynthesis of BIAs proceeds via a common pathway from tyrosine to (*S*)‐reticulene at which point the pathway diverges. Coclaurine *N*‐methyltransferase (CNMT) is a key enzyme in the pathway to (*S*)‐reticulene, installing the *N*‐methyl substituent that is essential for the bioactivity of many BIAs. In this paper, we describe the first crystal structure of CNMT which, along with mutagenesis studies, defines the enzymes active site architecture. The specificity of CNMT was also explored with a range of natural and synthetic substrates as well as co‐factor analogues. Knowledge from this study could be used to generate improved CNMT variants required to produce BIAs or synthetic derivatives.

Plants produce a wide range of bioactive benzylisoquinoline alkaloids (BIA) including the analgesics morphine and codeine, antitussive agent glaucine, muscle relaxant (+)‐tubocurarine, antitumor agent dauricine, as well as compounds that possess antimicrobial and other bioactivities. (Figures 1 & S1).^1^ Whilst some medically important BIAs can be isolated in high yields from plants, there are some BIAs of interest that are in limited supply.^1^, ^2^ In an effort to generate more robust platforms for BIA production, recent attention has focused on reconstituting BIA biosynthetic pathways in microbial host strains, such as *E. coli* and yeast, which are more genetically tractable and easily cultivated.^2^, ^3^ However, there are numerous challenges associated with producing BIAs in microbial hosts, including the low activity of some plant enzymes in microorganisms and the toxicity of some BIA biosynthetic intermediates to microbial hosts.^2^, ^3^ Moreover, a number of the enzymes from BIA pathways exhibit promiscuity leading to the production of multiple side‐products, which further reduce yields of desirable products.^2^

**Figure 1:**
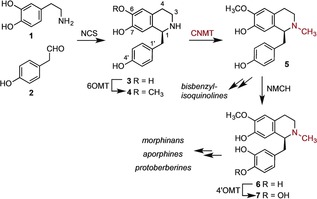
Early steps in the biosynthesis of benzylisoquinoline alkaloids (BIA) are catalysed by norcoclaurine synthase (NCS), norcoclaurine 6‐*O*‐methyltransferase (6OMT), coclaurine *N*‐methyltransferase (CNMT), *N*‐methyl‐coclaurine‐3′‐hydroxylase (NMCH), methylcoclaurine‐4′‐*O*‐methyltransferase (4′OMT) providing (*S*)‐reticulene **7** the main branching point.

In addition to their utility in microbial production platforms, enzymes from BIA biosynthesis have been exploited as biocatalysts to produce “non‐natural” products, which could provide additional bioactive scaffolds for drug development.^4^ For example, NCS has been utilized to produce a wide range of tetrahydroisoquinolines (THIQ) from simple synthetic precursors.^5^ Similarly the berberine bridge enzyme has also been used to produce non‐natural scoulerine analogues.^6^ Additionally, enzymes from BIA biosynthesis, have been combined with enzymes from other pathways to create novel products.^7^ The future exploitation of BIA biosynthetic enzymes for pathway engineering in vivo, or for enzymatic synthesis in vitro, is largely dependent on our increasing knowledge of the structure, mechanism and substrate scope of these important enzymes. Such insights can guide the engineering of enzymes towards improved activity or more stringent substrate specificity, which could reduce kinetic bottle necks or side‐product formation in engineered microbial BIA producing strains.^2^, ^3^ Similarly, engineered variants of BIA enzymes, with specificity for non‐natural substrates, could open up new routes to alternative bioactive THIQ scaffolds.^4^, ^5^, ^6^, ^7^ In this paper we describe the first crystal structure of CNMT, an essential enzyme in the core pathway to BIAs, which introduces the amino methyl functionality that is often essential for the bioactivity of most of the medically important BIAs (Figures 1 & S1). Active site mutagenesis and substrate screening provide further characterization of CNMT, which can guide engineering of CNMT for biosynthetic and synthetic applications.

Previous studies with CNMT from *Coptis japonica* showed that this *S*‐adenosyl‐l‐methionine (AdoMet) dependent enzyme can *N*‐methylate a number of natural THIQ, exhibiting highest activity with coclaurine **4** and heliamine **8**.^8^ To further characterize this enzyme, the *C. japonica* CNMT was overproduced in *E. coli* (Figure S2 in the Supporting Information). Initial attempts to obtain crystal structures in the presence of its natural substrates (**3** or **4**) proved unsuccessful. However, crystals of CNMT with *S*‐adenosyl‐l‐homocysteine (AdoHcy) and *N*‐methylheliamine **8 a** bound (PDB 6GKV, Figure 2 & Table S1), were obtained from co‐crystallization experiments set up in the presence of AdoMet and heliamine **8**, with methylation of **8** most likely occurring during the long incubation periods required for crystallization. The CNMT structure (Figures 2 & S3) reveals a typical class I methyltransferase α/β Rossmann fold forming the AdoMet‐binding domain, which is capped by a predominantly alpha helical BIA substrate recognition domain. CNMT shows a similar overall fold (RMSD of 1.1 Å) to the recently determined structure of the pavine *N*‐Methyltransferase (PavNMT).^9^ CNMT forms a dimeric interface made up of 13 hydrogen bonds and 3 salt bridges, similar to that seen in the PavNMT structure. The main differences observed between CNMT and PavNMT structures are located in close proximity to the active site.

**Figure 2:**
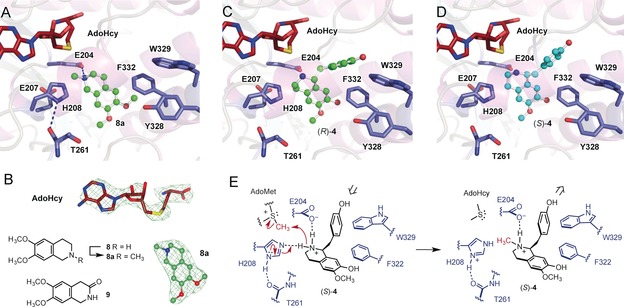
(**A**) The structure of CNMT (PDB 6GKV) with AdoHcy in red and the product *N*‐methylheliamine **8 a** in green (ball and stick) showing key amino acid residues involved in catalysis or substrate recognition. (**B**) m*F_o_‐mF_c_* electron density maps (contoured at 3.5 σ) of AdoHcy and **8 a** in the CNMT active site. Structure of substrate heliamine **8**, *N*‐methylated product **8 a** and substrate analog **9**. (**C**) Model of the CNMT (*R*)‐coclaurine **4** docked in the active site and (**D**) a model derived from docking (*S*)‐**4**. (**E**) Proposed mechanism of CNMT showing the putative role of key active site amino acid residues.

CNMT consists of a relatively smaller, more compact, active site (CNMT 387 Å^2^/PavNMT 1081 Å^2^) which is shaped by positioning an extended loop (residues 249–260) and residues 64–82 of helix α4 closer to the substrate binding pocket (Table S2). An apo structure was also determined with AdoHcy, but no substrate bound (PDB 6GKZ, Figure S3 & Table S1). This reveals that the helix α4 is disordered in the apo CNMT structure, however, in the presence of substrate becomes ordered and protrudes directly into the active site, forming a closed cap covering the substrate entrance, suggesting the helix α4 may control substrate accessibility and/or recognition by CNMT. A third structure (PDB 6GKY) was determined for CNMT in complex with a quinolinone substrate analog **9** (Figure S4). The structure of CNMT with **9** is very similar to the *N*‐methylheliamine **8 a** structure (Figure 2) except the two ligands are flipped relative to one another in the active site, with the carbonyl group of **9** in the same position as the amino group of **8 a**.

Modeling studies were also conducted for CNMT with the natural substrate coclaurine **4**, using the software ICM PRO Molsoft and the ligand bound structure of CNMT to guide the docking of both (*S*)‐ and (*R*)‐**4** (Figures 2 & S5). The top docking hits for (*S*)‐ and (*R*)‐**4** returned docking energies of *e*=−19.39 and −9.98 kcal mol^−1^, respectively. In both of these lowest energy structures the THIQ ring of (*S*)‐ and (*R*)‐**4** occupy the same position as the product **8 a**, except the ring is flipped; C1 of **8 a** is orientated towards H208, whilst the C1 *p*‐hydroxybenzyl substituent of (*S*)‐ and (*R*)‐**4** is on the opposite side of the active site orientated out of the active site. Overall the modeling shows both enantiomers of coclaurine can be accommodated in the CNMT active site, consistent with previous studies with this enzyme^8^ and related CNMTs^10^ which show that CNMTs exhibit little stereoselectivity and can methylate both (*S*)‐ and (*R*)‐**4**.

Three active site residues, E204, E207 and H208 are in close proximity to the methyl group of the product **8 a** in the CNMT structure. The amino group of substrates (**3**, **4** & **8**) have p*K*_a_ values of ca. 8.7–8.8, and will exist predominately as protonated ammonium ions in aqueous solution at neutral pH. Consequently, E204, E207, and H208 could potentially stabilize the substrate ammonium ion through H‐bonding or a salt bridge. One of these residues could also serve as a general base, deprotonating the ammonium ion to generate a nucleophilic free amine required for subsequent methylation. To explore these possibilities, E204, E207, and H208 were each mutated to alanine. The H208A mutant showed the most significant effect, resulting in very low enzyme activity of 1 and 4 % relative to wild‐type CNMT with substrates **8** and **3** respectively (Figure S6), suggesting H208 plays a key role in catalysis, most likely functioning as a general base to deprotonate the substrate ammonium ion. Sequence alignments with related NMTs including the PavNMT,^9^, ^11^ indicate that the H208 and E207 residues are highly conserved. Furthermore, mutation of these residues to alanine also results in a large decrease in the activity of PavNMT. Given that the E207 carboxyl is 7 Å from H208 imidazole, which is in turn 5 Å from the N atom of **8 a** in the CNMT structure, H208 and E207 may function as a catalytic diad. However, the E207A mutation did not have a significant impact, with the mutant retaining 60–75 % activity relative to the wild type (Figure S6). Moreover, the backbone carbonyl group of T261 is within H‐bonding distance of H208, which could similarly assist H208 in functioning as a general base. In contrast to E207A, the mutant E204A resulted in a significant drop in activity (4–6 % relative to wild‐type). The close proximity of E204 carboxyl to the N atom of **8 a** (4 Å) suggest that E204 may interact electrostatically with the substrate/product ammonium ion. The proximity of the residues Y328, W329, R330, G331, and F332 to **8 a** also indicates these residues may be involved in substrate binding. Single alanine mutations were carried out for all five positions and these mutants were tested with substrates **8** and **3**. The R330A and G331A mutations showed negligible effect on activity, whilst W329A and F332A resulted in a more significant decrease in activity (Figure S6), suggesting these aromatic residues may provide potential hydrophobic and π stacking interactions. Interestingly, whilst F332A resulted in a similar drop in activity with both substrates (**8** & **3**), the W329A mutant exhibited a more significant drop in activity with **3** than **8**, indicating that F332 may interact with the THIQ aryl group common to both substrates, whilst W329 may be interacting with the *p*‐hydroxybenzyl group of **3**. The fact that F332 is closer to the product **8 a**, in the CNMT structure, than W329, would also support this hypothesis (Figure 2). To explore this further, kinetic parameters of the WT along with mutants Y328A, W329A, and F332A were determined with substrates **8** and **3** (Table 1 & Figures S7–S13). The mutants all showed increased *K*_m_ values with the two substrates, signifying that these residues are important in substrate binding. Moreover, W329A afforded far slower turnover with **3,** exhibiting a *k*_cat_ value with **3** that is 13‐fold lower than with **8**, which is consistant with W329 participating in π‐π stacking or an edge‐to‐face interaction with the *p*‐hydroxybenzyl substituent of **3** that may stabilize the orientation of **3**, relative to AdoMet, for efficient methylation (Figure 2). Similarly, the kinetic parameters for F332A with **3** indicated that the F332 residue may also be involved in substrate binding through π‐π stacking. Kinetic parameters could not be determined for F332A with **8** due to lack of substrate saturation, which further supports the hypothesis that F332 interacts with the THIQ aryl ring common to **8** and **3**. The combined structural and mutagenesis studies, indicate that substrate binding in CNMT occurs through hydrophobic aromatic residues (F322, W329) as well as a salt bridge between E204 and the substrate ammonium ion, which is most likely deprotonated by H208, possibly aided by the backbone carbonyl group of T261, to generate the nucleophilic amine species required for methylation by AdoMet (Figure 2 E).

**Table 1:** Kinetic parameters of the CNMT wild‐type and mutants enzymes.

Sub.	Enzyme	*K*_M_ [μm]	*k*_cat_ [min^−1^]	*k*_cat_/*K*_M_ [min^−1^ μm ^−1^]×10^−3^
**8**	WT	311±18	35.9±0.6	120±7.3
	Y328A	2096±144	11.8±0.4	5.6±0.43
	W329A	1565±77	8.1±0.2	5.2±0.27
	F332A	ND	ND	ND
				
**3**	WT	265±31	31.9±1.1	120±14
	Y328A	601±84	7.0±0.3	12±1.7
	W329A	1033±64	0.6±0.01	0.63±0.045
	F332A	1726±142	1.2±0.04	0.70±0.062

Whilst earlier studies have shown that *C. japonica* CNMT accepts a number of natural THIQs as substrates, the broader synthetic potential of this enzyme has not been explored.^8^ Given that *N*‐methyl THIQ is a privileged pharmacophore, found in many pharmaceuticals, (Figure S1) we sought to explore the substrate scope of CNMT with synthetic substrates. *N*‐methylation can be achieved using non‐enzymatic chemistries, but often these methods use toxic, or otherwise deleterious, reagents and lack selectivity, which with more complex substrates can lead to side products or the laborious use of protective groups. Cleaner, milder and more selective biocatalytic methods for THIQ *N*‐methylation may thus be useful in drug synthesis. Accordingly, a colorimetric assay with CNMT and *S*‐adenosylhomocysteine hydrolase (Figure S14) was used to screen for alternative, non‐natural substrates (Figures S15 & S16). The conversions of the substrates to *N*‐methylated products (Figures 3 & S15), was then confirmed by HPLC and LCMS (Figures S17–S25). Coclaurine **4** and heliamine **8** proved to be the best substrates (90–92 % conversion), whilst norcoclaurine **3** and C1‐substituted THIQs **10**–**13** were turned over to lesser extent (85–88 %). The 6,7‐diethoxy derivative **14**, was also methylated at lower levels than the substrates possessing 6,7‐dimethoxy substituents. THIQ **16,** possessing a C1‐acetate substituent, was also a poor substrate, presumably because the carboxyl side chain is destabilized in a more hydrophobic binding pocket that accommodates the *p*‐hydroxybenzyl substituent of the natural substrate. A series of THIQs with C3‐ or C4‐substituents were also synthesized (Figure S16) and tested as CNMT substrates. THIQs **17** and **18**, with C4‐methyl substituents were *N*‐methylated (54 % and 22 % respectively, Figure 3). However, THIQs with larger C3‐ or C4‐substituents gave very low levels of methylation products (Figure S15). Thus whilst CNMT can accommodate THIQs with various C1‐substituents, the enzyme is less tolerant to modifications at C3 or C4. The CNMT structure (Figure 2) indicates that larger substituents at C3 or C4 would clash with E204, E207, H208, and I234 residues of the CNMT active site.

**Figure 3:**
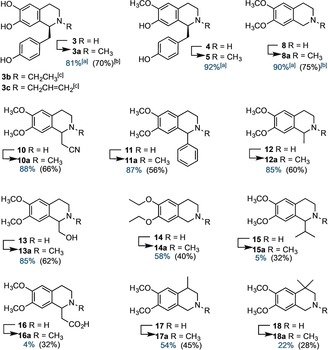
Substrate scope of CNMT showing [a] % conversions determined by HPLC 45 min assays [b] isolated yields from CNMT‐CLEA reactions and [c] products from alkylation with *S*‐ethyl and *S*‐allyl AdoMet (Figures S25 & S26).

Given that CNMT has relatively low catalytic activity even with native substrates (Table 1), immobilization of CNMT through the formation of cross‐linked enzyme aggregates (CLEAs)^12^ was explored in order to obtain a more robust biocatalyst. The CNMT‐CLEAs proved to have improved stability and can be recycled at least 3 times without significant loss of activity. In addition, using CNMT‐CLEAs allows the substrate concentration to be increased to 3–4 mm enabling biotransformations to be achieved on a preparative scale and methylated products to be isolated in good yields (Figure 3), even with poor substrates (e.g. **15**–**18**).

To further explore the biocatalytic scope of CNMT, AdoMet analogs with alternative *S*‐alkyl substituents were tested as co‐factors. AdoMet analogs have been used for alkyl diversification of natural products in an effort to generate variants with improved properties.^13^ Such biocatalytic alkyl diversification of THIQ is an attractive goal given the broad ranging bioactivity of these compounds. In addition to CNMT there are several other methyltransferases involved in biosynthesis of BIAs and related alkaloids, which could facilitate regioselective alkyl derivatization of these pharmacologically important compounds. To test this approach with CNMT, ethyl‐ and allyl‐AdoMet analogs were generated in situ, from l‐ethionine and *S*‐allyl‐l‐homocysteine using a mutant of the human methionine adenosyltransferase (hMAT2A I322V) as described previously.^13^ Both co‐factor analogues were accepted by CNMT, with **3** as a substrate, affording *N*‐ethyl and *N*‐allyl norcoclaurine **3 b** and **3 c** (Figures 3, S26 & S27).

In summary, we have determined the first structure of CNMT, which combined with active site mutagenesis suggest the likely function of key active site residues. The catalytic scope of CNMT was assessed with a range of natural and synthetic substrates, indicating that the enzyme exhibits relaxed substrate specificity with C1‐substituted THIQs. Finally, analogs of the AdoMet co‐factor were shown to be accepted by CNMT leading to *N*‐alkylated products. Taken together, the knowledge presented here will be valuable in structure‐guided engineering of CNMT to generate enzymes with improved properties required for microbial production of BIAs. Similarly engineered variants of CNMT with improved activity and specificity could provide new routes to synthetic drugs containing the privileged *N*‐alkyl THIQ pharmacophore.

## Conflict of interest

The authors declare no conflict of interest.

## Supporting information

As a service to our authors and readers, this journal provides supporting information supplied by the authors. Such materials are peer reviewed and may be re‐organized for online delivery, but are not copy‐edited or typeset. Technical support issues arising from supporting information (other than missing files) should be addressed to the authors.

Supplementary

## References

[1] J. M. Hagel , P. J. Facchini , Plant Cell Physiol. 2013, 54, 647–672. 23385146 10.1093/pcp/pct020

[2] L. Narcross , E. Fossati , L. Bourgeois , J. E. Dueber , V. J. J. Martin , Trends Biotechnol. 2016, 34, 228–241. 26775900 10.1016/j.tibtech.2015.12.005

[3a] A. Nakagawa , C. Matsuzaki , E. Matsumura , T. Koyanagi , T. Katayama , K. Yamamoto , F. Sato , H. Kumagai , H. Minami , Sci. Rep. 2014, 4, 1–8; 10.1038/srep06695 PMC4204043 25331563

[3b] A. Nakagawa , H. Minami , J.-S. Kim , T. Koyanagi , T. Katayama , F. Sato , H. Kumagai , Nat. Commun. 2011, 2, 326; 21610729 10.1038/ncomms1327 PMC3112539

[3c] E. Fossati , A. Ekins , L. Narcross , Y. Zhu , J. Falgueyret , G. A. W. Beaudoin , P. J. Facchini , V. J. J. Martin , Nat. Commun. 2014, 5, 1–11; 10.1038/ncomms4283 24513861

[3d] S. Galanie , K. Thodey , I. J. Trenchard , M. F. Interrante , C. D. Smolke , Science 2015, 349, 1095–1100. 26272907 10.1126/science.aac9373 PMC4924617

[4a] J. H. Schrittwieser , et al., RSC Adv. 2013, 3, 17602; 25580241 10.1039/c3ra42123f PMC4285126

[4b] H. Kries , S. E. O'Connor , Curr. Opin. Chem. Biol. 2016, 31, 22–30. 26773811 10.1016/j.cbpa.2015.12.006

[5a] B. M. Ruff , S. Bräse , S. E. O'Connor , Tetrahedron Lett. 2012, 53, 1071–1074; 22966211 10.1016/j.tetlet.2011.12.089 PMC3435518

[5b] B. R. Lichman , J. Zhao , H. C. Hailes , J. M. Ward , Nat. Commun. 2017, 8, 14883. 28368003 10.1038/ncomms14883 PMC5382262

[6] V. Resch , H. Lechner , J. H. Schrittwieser , S. Wallner , K. Gruber , P. MacHeroux , W. Kroutil , Chem. Eur. J. 2012, 18, 13173–13179. 22962029 10.1002/chem.201201895 PMC3533790

[7] V. Erdmann , B. R. Lichman , J. Zhao , R. C. Simon , W. Kroutil , J. M. Ward , H. C. Hailes , D. Rother , Angew. Chem. Int. Ed. 2017, 56, 12503–12507; 10.1002/anie.201705855 PMC5658969 28727894

[8a] K. B. Choi , T. Morishige , F. Sato , Phytochemistry 2001, 56, 649–655; 11314949 10.1016/s0031-9422(00)00481-7

[8b] K. B. Choi , T. Morishige , N. Shitan , K. Yazaki , F. Sato , J. Biol. Chem. 2002, 277, 830–835. 11682473 10.1074/jbc.M106405200

[9] M. A. Torres , E. Hoffarth , L. Eugenio , J. Savtchouk , X. Chen , J. S. Morris , P. J. Facchini , K. K. S. Ng , J. Biol. Chem. 2016, 291, 23403–23415. 27573242 10.1074/jbc.M116.747261 PMC5095397

[10] T. Frenzel , M. H. Zenk , Phytochemistry 1990, 29, 3491–3497.

[11a] D. K. Liscombe , J. Ziegler , J. Schmidt , C. Ammer , P. J. Facchini , Plant J. 2009, 60, 729–743; 19624470 10.1111/j.1365-313X.2009.03980.x

[11b] J. S. Morris , P. J. Facchini , J. Biol. Chem. 2016, 291, 23416–23427. 27634038 10.1074/jbc.M116.750893 PMC5095398

[12] R. A. Sheldon , Biochem. Soc. Trans. 2007, 35, 1583–1587. 18031271 10.1042/BST0351583

[13a] B. J. C. Law , A. Struck , M. R. Bennett , B. Wilkinson , J. Micklefield , Chem. Sci. 2015, 6, 2885–2892; 29403635 10.1039/c5sc00164a PMC5729408

[13b] B. J. C. Law , M. R. Bennett , M. L. Thompson , C. Levy , S. A. Shepherd , D. Leys , J. Micklefield , Angew. Chem. Int. Ed. 2016, 55, 2683–2687; 10.1002/anie.201508287 PMC4770447 26797714

