# Supplementary material for: Structure and Biocatalytic Scope of Coclaurine N‐Methyltransferase
Source: Angew Chem Int Ed Engl. 2018 Jun 28;57(33):10600–4. doi: 10.1002/anie.201805060 (PMC6099451; doi:10.1002/anie.201805060)
Supplement: Supplementary file 1 — Supplementary [file ANIE-57-10600-s001.pdf]

Angewandte Chemie
Eine Zeitschrift der Gesellschaft Deutscher Chemiker

Supporting Information

Structure and Biocatalytic Scope of Coclaurine *N*-Methyltransferase

*Matthew R. Bennett, Mark L. Thompson, Sarah A. Shepherd, Mark S. Dunstan, Abigail J. Herbert, Duncan R. M. Smith, Victoria A. Cronin, Binuraj R. K. Menon, Colin Levy, and Jason Micklefield**

anie_201805060_sm_miscellaneous_information.pdf

Content

Page S2-S17: Experimental

Page S18-S56: Supplementary Figures

Experimental

Cloning of CNMT. A synthetic gene codon optimised for *E. coli*, encoding for the *Coptis japonica* coclaurine *N*-methyltransferase (CNMT) was obtained from GeneArt (Invitrogen), and subcloned into the pET28a (+) vector using *EcoRI* and *XhoI* restriction sites for production of CNMT with an *N*-terminal His-tag.

Site-directed mutagenesis of CNMT. Mutagenic primers containing codon changes for E207, H208, Y328, W329, R330, G331 and F332 to Ala were used to introduce mutations by standard PCR techniques. Following PCR, the wild-type DNA was digested with the *Dpn*I restriction endonuclease. The mutant DNA was then used to transform chemically competent *E. coli* DH5α cells. Plasmids were subsequently extracted using a Qiagen miniprep spin kit and verified through sequencing (GATC Biotech).

Growth, expression and purification of CNMT. The wild-type and mutant CNMT encoding plasmids were used to transform *E. coli* BL21 (DE3) chemically competent cells for protein production. Transformants were grown on LB agar containing kanamycin (50 µg/mL) overnight. Single colonies from the agar plates were then used to inoculate LB media (containing kanamycin, 50 µg/mL) and then cultivated overnight at 37 °C with 180 rpm agitation. Fresh LB (kanamycin, 50 µg/mL) was then prepared and the overnight seeder cultures were diluted 100-fold before further cultivation at 37 °C with 180 rpm agitation until an OD_{600} of >0.6 was reached. Isopropyl β-D-1-thiogalactopyranoside (IPTG, 1 mM) was then added to induce protein production, and after induction cells were cultivated overnight at 30 °C, 180 rpm agitation. The cells were harvested by centrifugation (1600 *x g*, 10 min, 4 °C) and the resultant cell pellets were resuspended in lysis buffer (100 mM potassium phosphate buffer pH 7.0, 200 mM NaCl, 20 mM imidazole). The cell pellets were lysed by sonication and the lysate clarified by centrifugation (11,000 *x g*, 4 °C, 45 min). The soluble lysate was loaded onto Ni-NTA and rocked for two hours at 4 °C. The resin-bound CNMT was then loaded onto a gravity flow column and washed with 7 column volumes (CV) of Ni-NTA wash buffer (100 mM potassium phosphate, 200 mM NaCl, 60 mM imidazole) CNMT was then eluted with 5 CV of Ni-NTA elution buffer (100 mM potassium phosphate, 200 mM NaCl, 500 mM imidazole). The elution fraction of the CNMT protein was then dialysed and concentrated to approximately 1 mL of storage buffer (100 mM potassium phosphate pH 7.0, 200 mM NaCl) using a 30,000 MWCO Vivaspin 20 centricon (Sartorius Stedim Biotech). Analysis of all fractions by SDS-PAGE determined CNMT's purity (Figure S2). After concentration Glycerol (10% v/v) was added to all enzyme samples for –80 °C storage. In subsequent crystallography trials, further purification steps (anion exchange and gel filtration chromatography) were added to increase CNMT purity.

Anion exchange and gel filtration chromatography of CNMT for crystallography. Anion exchange chromatography was carried out using an ÄKTA prime FPLC with a HiTrap™ Q HP anion exchange column (GE Healthcare). The CNMT protein eluate from the Ni-NTA purification of CNMT was diluted into anion exchange buffer (20 mM Tris-HCl, 20 mM NaCl, pH 8) and loaded onto the anion exchange column. The column was washed with anion exchange wash buffer (20 mM Tris HCl, 50 mM NaCl, pH 8 buffer) before CNMT was eluted with anion exchange elution buffer (20 mM Tris-HCl pH 8, 1 M NaCl). Fractions were collected and analyzed by SDS-PAGE to determine CNMT

purity. Gel filtration was carried out using a Superdex™ 200 10/300 GL column (GE Healthcare) on an ÄKTA purifier FPLC system. Fractions, containing CNMT (from the anion exchange purification), in anion exchange elution buffer (20 mM Tris-HCl pH 8, 1 M NaCl) were pooled and buffer exchanged into gel filtration buffer (25 mM Tris-HCl pH 8.4, 150 mM NaCl) using a Vivaspin 20 centricon (10,000 MWCO). The CNMT protein was concentrated to approximately 10 mg/mL and was loaded onto the column in 0.5 mL injections. Using an isocratic gradient of 100% gel filtration buffer at a flow rate of 1 mL/min fractions were collected in a 96-deep well block and analyzed by SDS-PAGE, to determine purity of CNMT. Pure samples of CNMT were concentrated and taken forward to crystallography trials.

Crystallization, refinement and model building. A selenomethionine (SeMet) derivative of CNMT, prepared by standard procedures, was concentrated to 35 mg/ml in the presence of 5 mM AdoHcy. Crystals were obtained using the sitting-drop vapour-diffusion method and grew within 10 days at 4 °C in 0.12 M Ethylene Glycol 0.1 M HEPES/MOPS (pH 7.4) 37.5 % MPD_PEG 1K/PEG 3350 and 1 mM AdoHcy. A highly redundant Single Anomalous diffraction (SAD) data set was collected from a single flash-cooled crystal at the Diamond light source (beamline IO4) and reflections merged and scaled with Xia2. Initial phases for CNMT were obtained using AUTO-RICKSHAW package[S1] followed by density modification with RESOLVE.[S2] Initial model building was done with Buccaneer followed by iterative cycles of manual model building and refinement in COOT and Phenix.refine. Crystals containing ligands were obtained in 0.09 M Halogens 0.1 M Imidazole; MES (pH6.5), 30% P500MME_P20K and 1 mM Ligand. Crystals with *N*-methylheliamine **8a** were obtained using heliamine **8** and 1 mM AdoMet and crystals were also obtained with quinolinone substrate analog **9** with 1 mM AdoHcy. Crystals routinely took between 10-15 days to grow and were directly frozen in liquid nitrogen. For final data and refinement statistics see **Table S1**.

Colorimetric assay with CNMT and *S*-adenosylhomocysteine hydrolase (SAHH). A synthetic gene for SAHH (origin: *Rattus norvegicus*) optimised for *E. coli* was obtained from GeneArt (Invitrogen) and subcloned into the pET28a (+) vector containing an *N*-terminal His-tag. *E. coli* BL21 (DE3) cells were subsequently transformed with the construct for overexpression of the recombinant protein with purification routinely performed using Ni^{2+} affinity chromatography. Substrate screening assays were carried out at a volume of 150 µL in 100 mM phosphate buffer using 5 µM CNMT, 80 µM AdoMet and 80 µM substrate. Samples were incubated for 30 min before heating at 95˚ C for 5 min to precipitate the CNMT which was subsequently removed via centrifugation. Samples were transferred to a microtitre plate and chromene (2mM) was added prior to the addition of 1 µM SAHH immediately before sampling in the plate reader every 1 min for 25 min at 37˚ C, measuring absorbance at 405 nm.

Enzyme activity assays. The substrates norcoclaurine (**3**), coclaurine (**4**), heliamine (**8**), 2-(6,7-dimethoxy-1,2,3,4-tetrahydroisoquinolin-1-yl)acetonitrile (**10**), 6,7-dimethoxy-1-phenyl-1,2,3,4-tetrahydroisoquinoline (**11**), 6,7-dimethoxy-1-methyl-1,2,3,4-tetrahydroisoquinoline (**12**), (6,7-dimethoxy-1,2,3,4-tetrahydroisoquinolin-1-yl)methanol (**13**), 6,7-diethoxy-1,2,3,4-tetrahydroiso-quinoline (**14**), 1-isopropyl-6,7-dimethoxy-1,2,3,4-tetrahydroisoquinoline (**15**), 2-(6,7-dimethoxy-1,2,3,4-tetrahydroisoquinolin-1-yl)acetic acid (**16**), 6,7-dimethoxy-4-methyl-1,2,3,4-tetrahydro-

isoquinoline **(17),** 6,7-dimethoxy-4,4-dimethyl-1,2,3,4-tetrahydroisoquinonline **(18)**, 6-methoxy-1,2,3,4-tetrahydroisoquinoline **(S1)**, 6-chloro-1,2,3,4-tetrahydroisoquinoline **(S2)**, 6,7-Dimethoxy-4-ethyl-1,2,3,4-tetrahydroisoquinoline **(S3)**, 6,7-Dimethoxy-4-propyl-1,2,3,4-tetrahydroiso-quinoline **(S4)**, 4-benzyl-6,7-dimethoxy-1,2,3,4-tetrahydroisoquinoline **(S5)**, (*S*)-6,7-dimethoxy-1,2,3,4-tetrahydroisoquinoline-3-carboxylic acid **(S6)**, methyl (*S*)-6,7-dimethoxy-1,2,3,4-tetra-hydroisoquinoline-3-carboxylate **(S7)**, (*S*)-6,7-Dimethoxy-3-hydroxymethyl-1,2,3,4-tetrahydro-isoquinoline **(S8)**, were assayed with WT CNMT in following reaction mixtures: 3 mM AdoMet, 0.5 mM substrate, 5 µM CNMT in 100 mM phosphate buffer at pH 7. The reactions were incubated at 30°C, with 800 rpm agitation for 45 min in an Eppendorf Thermomixer before assays were quenched with an equal volume of methanol. Protein precipitate was removed by centrifugation and the assays were analyzed by C_{18} RP-HPLC using a Shimadzu Prominence UFLC XR HPLC system: (Phenomenex Kinetex C_{18} 5 µm 4.6 x 250 mm column, flow rate 1 mL/min, wavelength 283 nm; gradient (**Method I for reactions with 8, 10, 12, 15 & 16**) 0-1 min 5% B, 1-2min 5-10% B, 2-11 min 10-33% B, 11-13 min 95% B, 13-15 min 5% B(**Method II for reactions with 11 & 14**) 0-1 min 5% B, 1-2 min 10% B, 2-11 min 10-75% B, 11-11.1 min 95% B, 11.1-13 min 95% B, 13.1-15 min 5% B; gradient (**Method III for reactions 3, 4, 13, 17, S7)** 0-2 min 5% B, 2-3 min 5-15% B, 3-10 min 15% B, 10-10.1 min 15-95% B, 10.1-12 min 95% B, 12-12.1 min 95-5% B and 12.1-14.5 min 5% B. Mobile phase A consisted of H_2O + 0.05% TFA, mobile phase B consisted of acetonitrile + 0.05% TFA. Phenomenex Kinetex Phenyl-Hexyl 2.6 µm 4.6 x 100 mm column, flow rate 1.85 mL/min, wavelength 280 nm; gradient (**Method IV for reactions with 18, S1-S6 , S8**) 0-0.75 min 5% B, 0.75-1.5 min 5-15% B, 1.5-6 min 15% B, 6-6.1 min 15-95% B, 6.1-7.5 min 95% B, 7.5-7.6 95-5% B, 7.6-8.7 min 5% B. Mobile phase A consisted of H_2O + 0.1% FA, mobile phase B consisted of methanol + 0.1% FA. Substrate and product peaks on the HPLC chromatogram for each were confirmed by LCMS and comparison with authentic substrate standards that were either synthetic or isolated from CLEA reactions (see below). Calibration curves were used to adjust HPLC peak areas to account for differences in extinction coefficients between substrates and products. The relative activities were calculated as the percentage of product formed from the starting material in the time period stated.

Enzyme mutant activity assays. Assays were conducted with E207A, H208A, Y328A, W329A, R330A, G331A and F332A mutants following the same conditions as noted above except the assays were incubated for 30 minutes.

Michaelis-Menten enzyme kinetics. Kinetic constants of wild-type and mutant CNMT were determined through the following approach: CNMT enzymes (1-6 µM) were assayed against a range of substrate (**3** or **8**) concentrations (100-5000 µM) with the initial rate of reaction measured by monitoring substrate conversion over time points of 2, 5 and 10 minutes. The rates were plotted against substrate concentrations using Sigmaplot 12.0 and the K_m and k_{cat} constants generated from the resulting Michaelis-Menten plot.

CNMT Alkylation assays. Initial assays were conducted as described previously (see Law *et al* 2016 ref. 14b) with hMAT2a I322V (50 µM), $MgCl_2$ (3 mM), ATP (2 mM), ethionine or *S*-allyl-L-homocysteine (2 mM) in a 20 mM potassium phosphate buffer pH 7.4 incubated at 37° C for 2 hours with shaking at 800 rpm to prepare enzymatic *S*-adenosyl-allyl-L-homocysteine and *S*-

adenosyl-L-ethionine respectively. The enzymatically produced AdoMet analogs (1 mM) were added to 100 µM WT CNMT, 0.5 mM norcoclaurine **3**, in 100 mM phosphate buffer at pH 7.0 and were shaken at 800 rpm overnight. The assays were terminated with an equal volume of methanol and analyzed using an Agilent HPLC system following method II above.

CNMT cross-linked enzyme aggregates (CLEA) production and biotransformations. The wild-type CNMT expression plasmid was used to transform *E. coli* BL21 (DE3) containing pGro7 (Takara) chemically competent cells for protein production. The cells were maintained on LB agar containing kanamycin (50 µg/mL) and chloramphenicol (25 µg/mL). Single colonies from the agar plates were then used to inoculate LB media (containing kanamycin, 50 µg/mL and chloramphenicol, 25 µg/mL) and the seed cultures were cultivated overnight at 37 °C, 180 rpm agitation. Fresh LB (kanamycin, 50 µg/mL and chloramphenicol, 25 µg/mL) was then prepared and the overnight seeder cultures were diluted 100-fold and cultivated further at 37 °C with 180 rpm agitation until an OD_{600} of >0.6 was reached. Arabinose (1 mg/mL) and isopropyl β-D-1-thiogalactopyranoside (IPTG, 1 mM) were then added to induce protein production, and after induction cells were cultivated overnight at 30 °C, 180 rpm agitation. The cells were harvested by centrifugation (1600 *x g*, 10 min, 4 °C) and the resultant cell pellets were resuspended in lysis buffer (100 mM sodium phosphate buffer pH 7.0, 30 mL) and lysed by sonication. The cell lysates were clarified through centrifugation (10000 *x g*, 30 minutes) and the soluble lysate was precipitated over 2 hours at 4 ˚C with gentle rocking through addition of ammonium sulfate (16.2 g, 95 % saturation). After 2 hours the cross linker glutaraldehyde (250 µL) was added and the protein was allowed to aggregate for a further 2 hours at 4˚C. The solution was centrifuged (10000 *x g*, 30 minutes) and the supernatant discarded. The solid CLEA was then washed three times with lysis buffer (100 mM sodium phosphate buffer pH 7), before being resuspended in lysis buffer (5 mL). To this solution distilled water (25 mL) was added as well as the substrate (3 mM final concentration) and AdoMet (6 mM) in solid form. The reaction mixture was then incubated for 1-3 days at room temperature and analyzed by HPLC to determine completion of the reaction. The CNMT-CLEA was then removed by centrifugation (10,000 *x g*) and the supernatant was adjusted to pH 3 with 1.2 M HCl (aq) before being washed with diethyl ether (50 mL). The aqueous fraction was then adjusted to pH 10 with 10 M NaOH (aq), extracted with diethyl ether (3 x 50 mL) and the organic extracts were evaporated under reduced pressure to provide the title compounds listed below.

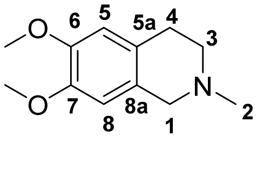

8a (75 %)

***N*-methylheliamine 8a** was prepared, following 16hr incubation period as described above. The product **8a** was obtained as a yellow solid (13.8 mg, 75%). ^{1}H NMR (400 MHz, $CDCl_3$) δ 6.60 (s, 1H, H5), 6.51(s, 1H, H8), 3.85 & 3.84 (2 x s, 3H, OCH_3), 3.51 (s, 1H, H1), 2.84 (t, *J* = 6.0 Hz, 2H, H4), 2.67 (t, *J* = 6.0 Hz, 2H, H3), 2.45 (s, 3H, NCH_3). ^{13}C NMR (100 MHz, $CDCl_3$) δ 147.5, 147.1 (C6 & C7),

126.5, 125.7 (C5a & C8a), 111.3 (C5), 109.3 (C8), 57.6 (C1), 55.9, 55.9 (2 x OCH_3), 52.9 (C3), 46.0 (*N*-C2), 28.8 (C4). ESI-HRMS mass calculated for $C_{12}H_{18}NO_2^+$ 208.1332; found 208.1329.

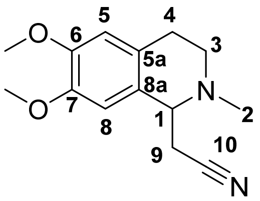

10a (66 %)

***N*-methyl-2-(6,7-dimethoxy-1,2,3,4-tetrahydroisoquinolin-1-yl)acetonitrile 10a** was prepared, following 16hr incubation period as described above, except that after the CNMT-CLEA was removed by centrifugation the resulting solution was loaded onto an Agilent C18 BondElut column, which was washed with H_2O (15 mL) and aqueous MeOH (25% MeOH) (15 mL) before elution of the product with MeOH. The eluent was evaporated under reduced pressure to give the product **10a** as an off-white solid (12.1 mg, 66%) ^{1}H NMR (400 MHz, 1:1 CD_3OD/D_2O) δ 6.82, 6.78 (2 x s, 2H, H5 & H8), 3.91 (t, *J* = 5.4, 1H, H1), 3.82 (2 x s, 6H, OCH_3), 3.11-3.04 (m, 1H, H3a), 3.00 (m, 2H, H9), 2.80 (m, 2H, H4), 2.76-2.65 (m, 2H, H3b), 2.48 (s, 3H, NCH_3); ^{13}C NMR (100 MHz, 1:1 CD_3OD/D_2O) δ 149.1, 148.3 (C6 & C7) 128.4, 127.7 (C5a & C8a) 120.4 (C10) 112.8, 111.3 (C5 & C8), 60.6 (C1), 56.7, 56.6 (2 x OCH_3), 49.2 (C3), 43.0 (NCH_3), 26.9 (C4) 23.3 (C9) HRMS ES$^+$MS, mass calculated for $C_{14}H_{20}NO_2^+$: 247.1441; found 247.1448.

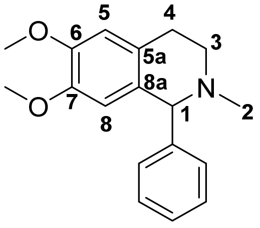

11a (56 %)

***N*-methyl-6,7-dimethoxy-1-phenyl-1,2,3,4-tetrahydroisoquinoline** (**11a**) was prepared, following 16hr incubation period as described above, except that after the CNMT-CLEA was removed by centrifugation the resulting solution was adjusted to pH 10 with 2 M NaOH (10 mL) and extracted with chloroform (3 x 40 mL). The organic fractions were combined and washed with brine (120 mL) and evaporated under reduced pressure to give **11a** as a yellow solid (14.0 mg, 56%). ^{1}H NMR (400 MHz, $CDCl_3$) δ 7.30-7.21 (m, 5H, Ph), 6.58, 6.08 (2 x s, 2H, H5 & H8), 4.16 (s, 1H, H1), 3.82 & 3.54 (2 x s, 3H, -OMe), 3.14 (m, 1H, H3), 3.07 (m, 1H, H4), 2.72 (m, 1H, H3’), 2.59 (m, 1H, H4’) 2.21 (s, 3H, NCH_3). ^{13}C NMR (100 MHz, $CDCl_3$) δ 147.4 & 147.0 (C6 & C7), 143.9 (Ph), 130.4 & 126.6 (C5a & C8a), 129.5, 128.3 & 127.3 (Ph), 111.5 & 110.7 (C5 & C8), 71.1 (C1), 55.8 & 55.8 (2 x OCH_3), 52.2 (C3), 44.3 (NCH_3), 29.0 (C4). ESI HRMS mass calculated for $C_{18}H_{22}NO_2^+$ 284.1645; found 284.1636.

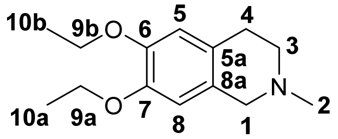

14a (48 %**)**

***N*-methyl-6,7-diethoxy-1,2,3,4-tetrahydroisoquinoline (14a)** was prepared, following 24 hrs incubation period as described above. The product **14a** was obtained as a yellow solid (7 mg, 48%). ^{1}H NMR (400 MHz, CD_3CN/D_2O (1:1)) δ 6.70 & 6.62 (2 x s, 2H, H5 & H8), 3.96 (m, 4H, H9a & H9b) 3.71 (s, 2H, H1), 2.91 (t, J = 6.0 Hz, 2H, H3), 2.81 (t, J = 6.0 Hz, 1H, H4), 2.52 (s, 3H, NCH_3) 1.29 (m, 6H, 10a & 10b). ^{13}C NMR (100 MHz, 1:1 CD_3CN/D_2O) 147.3 & 146.7 (C6 & C7), 124.8, 123.3 (C5a & C8a), 113.2 & 111.2 (C5 & C8), 64.6 & 64.6 (C9a & C9b), 55.5 (C1) 51.9 (C3), 43.7 (NCH_3), 26.4 (C4), 14.1 (C10a & C10b). ESI HRMS mass calculated for $C_{14}H_{21}NO_2^+$ 236.1645; found 236.1639.

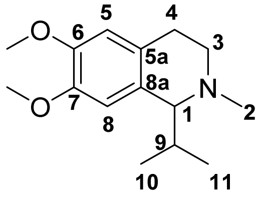

15a (32 %)

***N*-methyl-1-isopropyl-6,7-dimethoxy-1,2,3,4-tetrahydroisoquinoline (15a)** was prepared, following 72 hr incubation period as described above. The product **15a** was obtained as a yellow solid (5 mg, 32%) ^{1}H NMR (400 MHz, 1:1 CD_3CN/D_2O) δ 6.70 & 6.61 (2 x s, 2H, H5, H8) 3.72 & 3.71 (2 x s, OCH_3), 3.39 (d, J = 6.1, 1H, H1), 3.22 (m, 1H, H3a), 2.80-2.65 (m, 3H, H4 & H3b), 2.41 (s, 3H, H2), 1.91 (1H, m, H9), 0.95 & 0.76 (2 x d, J = 7.0 3H, $C(C\underline{H}_3)_2$). ^{13}C NMR (100 MHz, 1:1 CD_3CN/D_2O) δ 147.7 & 146.6 (C6 & C7), 112.2 & 111.7 (C5 & C8), 69.5 (C1), 55.7 & 55.6 (2 x OCH_3), 46.8 (C3), 42.5 (NCH_3), 33.3 (C9), 23.4 (C4), 19.8 & 19.4 (C10 & C11). ESI HRMS mass calculated for $C_{18}H_{22}NO_2^+$ 250.1802; found 250.1795.

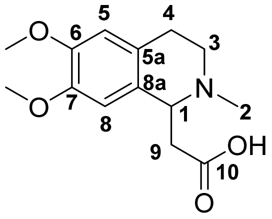

16a (29 %**)**

***N*-methyl-2-(6,7-dimethoxy-1,2,3,4-tetrahydroisoquinolin-1-yl)acetic acid (16a)** was prepared, following 72 hr incubation period as described above, except after removal of the CNMT-CLEA the resulting solution was lyphophilised overnight. The residue was then dissolved in 3 mL of acetonitrile and water (1:1) and purified by HPLC following method I described above, to give the product **16a** as a yellow solid (6.9 mg, 29%) ^{1}H NMR (400 MHz,) δ 6.83 & 6.75 (2 x s, 2H, H5 & H8), 4.68 (m, 1H, H1), 3.76 & 3.74 (2 x s, 3H, OMe), 3.63 (m, 1H, H3a) 3.34 (m, 1H, H3b), 3.08 (m, 2H, H9), 3.02 (m, 2H, H4), 2.94 (s, 3H, NCH_3); ^{13}C NMR (100 MHz) δ 174.1 (C10), 148.2 & 147.6 (C6 &

C7) 123.1 (C5a) 121.3 (C8a), 111.6 & 109.7 (C5 & C8), 60.5 (C1), 55.7, 55.6 (2 x OCH_3), 46.5 (C3), 40.1 (NCH_3), 37.7 (C9), 21.5 (C4); ESI HRMS mass calculated for $C_{14}H_{20}NO_2^+$ 266.1387; found 266.1383.

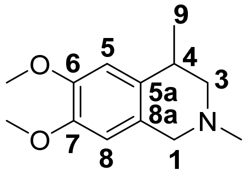

17a (45 %**)**

***N*-methyl-6,7-dimethoxy-4-methyl-1,2,3,4-tetrahydroisoquinoline (17a)** was prepared, following 24 hr incubation period as described above with 1.3 mM substrate, after removal of the CNMT-CLEA the resulting solution was evaporated. The residue was then dissolved in 2 mL of methanol and water (1:1) and purified by HPLC following method III described above, to give the product **17a** as an off white solid (4.0 mg, 45%) ^{1}H NMR (400 MHz, $CDCl_3$) δ 6.72 (s, 1H, H5), 6.50 (s, 1H, H8), 3.86 & 3.84 (2 x s, 3H, OCH_3), 3.59 (s, 2H, H1), 3.05 (m, 1H, H4), 2.88 (m, 1H, H3), 2.50 (m, 3H, H2), 2.39 (m, 1H, H3'), 1.30 (d, *J* = 7.0 Hz, 3H, H9). ^{13}C NMR (100 MHz, $CDCl_3$) δ 148.4, 147.8 (C6 & C7), 131.0 (C5a), 126.0 (C8a), 110.1 (C5), 109.0 (C8), 60.3, (C3), 57.6 (C1), 55.8 (2 x OCH_3), 45.5 (N$\underline{C}H_3$), 32.1 (C4), 20.4 (C9). Carbon data from HMBC and HSQC. HRMS (ESI+) calculated for $C_{13}H_{20}NO_2^+$ 222.1489 found 222.1488.

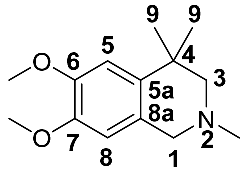

18a (28 %**)**

***N*-methyl-6,7-dimethoxy-4,4-dimethyl-1,2,3,4-tetrahydroisoquinoline (18a)** was prepared, following 24 hr incubation period as described above, except after removal of the CNMT-CLEA the resulting solution was evaporated. The residue was then dissolved in 2 mL of methanol and water (1:1) and purified by HPLC following method IV described above, to give the product **18a** as a yellow solid (4.9 mg, 28%). ^{1}H NMR (400 MHz, $CDCl_3$) δ 6.77 (s, 1H, H5), 6.50 (s, 1H, H8), 3.96 (s, 1H, H1), 3.87 (s, 3H, OCH_3), 3.83 (s, 3H, OCH_3), 2.86 (s, 1H, H3), 2.74 (s, 3H, H2), 1.38 (s, 6H, H9); ^{13}C NMR (100 MHz, $CDCl_3$) δ 149.0 (C6/7), 148.0 (C6/7), 133.4 (C5a), 121.2 (C8a), 108.8 (C5), 108.7 (C8), 65.1 (C3), 56.3 (C1), 56.3 (OCH_3), 56.0 (OCH_3), 45.0 (N$\underline{C}H_3$), 34.7 (C4), 30.0 (C9); ESI-HRMS mass calculated for $C_{14}H_{22}NO_2^+$ 236.1645, found 236.1642.

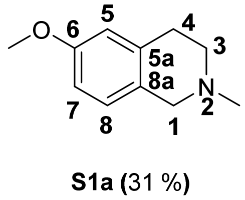

S1a (31 %**)**

6-methoxy-*N*-methyl-1,2,3,4-tetrahydroisoquinoline (S1a) was prepared, following 24 hr incubation period as described above, except after removal of the CNMT-CLEA the resulting solution was evaporated. The residue was then dissolved in 2 mL of methanol and water (1:1) and purified by HPLC following method IV described above, to give the product **S1a**. (5.0 mg, 31%). ^{1}H NMR (400 MHz, $CDCl_3$) δ 6.99 (d, *J* = 8.5 Hz, 1H, H8), 6.78 (dd, *J* = 8.5, 2.6 Hz, 1H, H7), 6.68 (d, *J* = 2.6 Hz, 1H, H5), 4.07 (s, 2H, H1), 3.78 (s, 3H, OCH_3), 3.25 (t, *J* = 6.3 Hz, 2H, H3), 3.12 (t, *J* = 6.3 Hz, 2H, H3); ^{13}C NMR (100 MHz, $CDCl_3$) 159.3 (C6), 132.4 (C5a), 128.0 (C8), 120.6 (C8a), 113.7 C5/7), 113.5 (C5/7), 55.5 (OCH_3), 54.3 (C1), 50.8 (C3), 42.4 (NCH_3), 25.8 (C4); ESI-HRMS mass calculated for $C_{11}H_{16}NO^+$ 178.1226, found 178.1223.

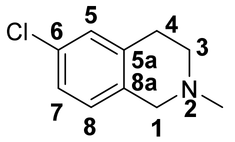

S2a (44 %)

6-chloro-*N*-methyl-1,2,3,4-tetrahydroisoquinoline (S2a) was prepared, following 48 hr incubation period as described above, except after removal of the CNMT-CLEA the resulting solution was evaporated. The residue was then dissolved in 2 mL of methanol and water (1:1) and purified by HPLC following method IV described above, to give the product **S2a**. (7.2 mg, 44 %). ^{1}H NMR (400 MHz, $CDCl_3$) δ 7.17 (m, 2H, H5 & H7), 7.00 (d, *J* = 8.9 Hz, 1H, H8), 3.82 (s, 2H, H1), 2.99 (m, 4H, H3 & H4), 2.63 (s, 3H, NCH_3); Due to limited solubility of the compound we were unable to obtain ^{13}C NMR; ESI-HRMS mass calculated for $C_{10}H_{13}NCl^+$ 182.0731, found 182.0731.

Synthesis of 3 & 4 substituted substrates

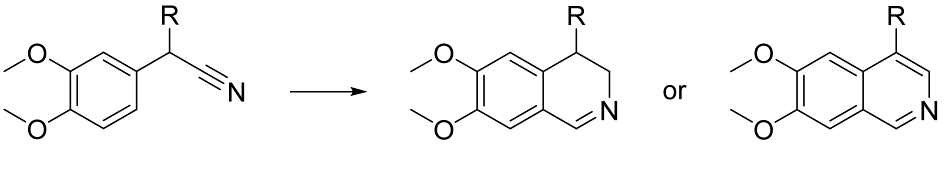

Synthetic procedure 1: Reduction and cyclisation of substituted nitriles. Under a nitrogen atmosphere, lithium aluminium hydride (2M solution in diethyl ether or 1M solution in tetrahydrofuran (THF) (1.6 mmol, 1.01 eq) was added to a flame-dried round bottom flask and cooled to 0 °C using an ice bath. Anhydrous THF was added dropwise to a total volume of 5 mL. A solution of the appropriately substituted nitrile (1.58 mmol, 1 eq) in anhydrous THF (3 mL) was added dropwise, after which the solution was heated to reflux (90 °C) for 1 hour. The solution was cooled in an ice bath and quenched by the sequential, dropwise addition of water (200 µL), sodium hydroxide solution (0.5 M, 150 µL) and water (700 µL).[S3] The supernatant was decanted and filtered, and the solid residue was washed with tetrahydrofuran (3 x 50 mL). The THF washes were combined and solvent removed under reduced pressure to afford a yellow oil, which was used without further purification.

The amine prepared above (approx. 1 mmol) was transferred to a vial fitted with a septum and cooled to 0 °C using an ice bath. Formic acid (1.4 eq) was added dropwise and the mixture was heated to 190 °C for 2 hours. After cooling to room temperature, toluene (1 mL) and phosphorous pentachloride (1.2 eq) were added and the mixture was heated to 120 °C for 1 hour. The solution was cooled in an ice bath, quenched by dropwise addition of water (5 mL), and washed with diethyl ether (3 x 5 mL). The pH of the aqueous layer was adjusted to 12 using sodium bicarbonate and sodium hydroxide solution (10% w/v), after which the basic solution was washed with diethyl ether (3 x 10 mL). The combined organic phases were dried over anhydrous sodium sulfate and solvent removed under reduced pressure to yield 3,4-dihydroisoquinolines, which were reduced according to synthetic procedure 2. In some cases, exposure of ethanolic solutions of 3,4-dihydroisoquinolines to air resulted in spontaneous oxidation to the isoquinolines,[S4] which were reduced according to synthetic procedure 3.

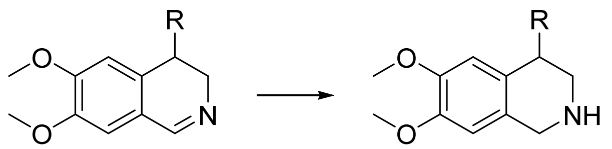

Synthetic procedure 2: Reduction of 3,4-dihydroisoquinolines using sodium borohydride. Substituted 3,4-dihydroisoquinolines (approx. 1 mmol), prepared according to synthetic procedure 1, was dissolved in ethanol (40 mL) and treated with sodium borohydride (4 eq).[S5] The mixture was stirred at room temperature overnight, after which solvent was removed under reduced pressure. The residue was dissolved in water (5 mL) and extracted with diethyl ether (3 x 5 mL) The combined organic phases were dried over anhydrous magnesium sulfate and the solvent removed under reduced pressure. The residue was purified by silica gel chromatography (1:9 methanol:dichloromethane) or preparative RP-HPLC to afford the 1,2,3,4-tetrahydroisoquinolines.

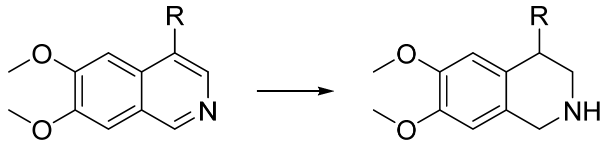

Synthetic procedure 3: Reduction of isoquinolines using lithium triethylborohydride was achieved following the method reported by Blough and Carroll,[S6] as follows. Substituted 3,4-dihydroisoquinolines (approx. 0.015 mmol), prepared according to synthetic procedure 1, were dissolved in THF (10 mL) under an atmosphere of nitrogen. A solution of lithium triethylborohydride (1 M in THF, 33 µL, 0.033 mmol, 2.2 eq) was added and the mixture was stirred at room temperature for 30 minutes. The reaction was quenched by addition of methanol (10 mL) before solvent was removed under reduced pressure. The residue was suspended in water (10 mL) and acidified to pH 2 using hydrochloric acid (1 M). The mixture was washed with diethyl ether (3 x 10 mL) before the aqueous phase was basified to pH 8 using a saturated solution of sodium bicarbonate and extracted with dichloromethane (3 x 10 mL). The combined organic fractions were dried over anhydrous sodium sulfate and the solvent removed under reduced pressure to afford the 1,2,3,4-tetrahydroisoquinolines.

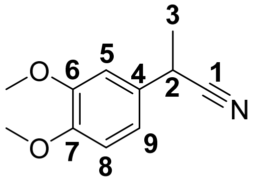

S16 (25%)

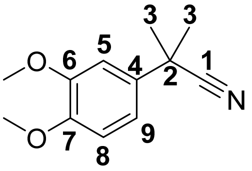

S17 (18%)

2-(3,4-Dimethoxyphenyl)propionitrile (S16) & **2-(3,4-dimethoxyphenyl)-2-methylpropionitrile (S17).** Alkylation of 6,7-dimethoxyphenylacetonitrile **S15** was carried following standard procedures as described previously.[S7] **S15** (2.660 g, 15 mmol) in THF (2 mL) was added dropwise to a solution of lithium diisopropylamide (2M solution in THF/heptane/ethylbenzene, 12 mL, 24 mmol, 1.6 eq) in THF (30 mL) at –78 ˚C under N_2. After 1 hour, methyl iodide (780 µL, 12.5 mmol) was added dropwise. After 30 minutes, the solution was warmed to room temperature. After 1 hour, the reaction was quenched by dropwise addition of ammonium chloride solution (10% w/v, 30 mL) and extracted with hexane (3 x 50 mL). The combined organic phases were washed with brine and dried over anhydrous magnesium sulfate. Solvent was removed under reduced pressure and the residue purified by silica gel column chromatography (3:1 hexane:ethyl acetate) to give 2-(3,4-Dimethoxyphenyl)propionitrile (**S16**) as an off-white solid (750 mg, 3.7 mmol, 25%). ^{1}H NMR ($CDCl_3$, 400 MHz) δ 6.86 (m, 3H, H5, H8 & H9), 3.89 (m, 7H, 2x OMe & H2), 1.64 (d, *J* = 7.2 Hz, 3H, H3); ^{13}C NMR ($CDCl_3$, 100 MHz) δ 149.4 (C6/7), 148.8 (C6/7), 129.5 (C4), 121.8 (C1), 118.9 (C9), 111.5 (C5), 109.8 (C8), 56.0 (OCH_3), 30.9 (C2), 21.5 (C3); HRMS (ESI+) calculated for $[M\text{-}HCN]^+$ $C_{10}H_{13}O_2^+$ 165.0910, found 165.0916. The dialkylated product **S17** was also isolated as a yellow oil (570 mg, 2.8 mmol, 18%). ^{1}H NMR ($CDCl_3$, 400 MHz) δ 6.92 (m, 2H, H5 & H8), 6.79 (d, *J* = 8.1 Hz, 1H, H9), 3.84 (s, 3H, OMe), 3.81 (s, 2H, OMe), 1.64 (s, 6H, H3); ^{13}C NMR ($CDCl_3$, 100 MHz) δ 149.1 (C6/7), 148.6 (C6/7), 134.0 (C4), 124.7 (C1), 117.1 (C9), 111.2 (C5), 108.8 (C8), 56.0 (OCH_3), 56.0 (OCH_3), 36.7 (C2), 29.3 (C3); HRMS (ESI+) calculated for $[M\text{-}HCN]^+$ $C_{11}H_{15}O_2^+$ 179.1067, found 179.1069

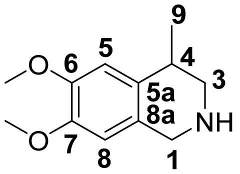

17 (12%)

6,7-dimethoxy-4-methyl-1,2,3,4-tetrahydroisoquinoline (**17**) was prepared from 2-(3,4-dimethoxyphenyl)propionitrile (**S16**) according to synthetic procedures 1 and 3, and isolated as a pale yellow oil (36 mg, 0.18 mmol, 12%). ^{1}H NMR (400 MHz, $CDCl_3$) δ 6.69 (s, 1H, H5), 6.49 (s, 1H, H8), 3.93 (s, 2H, H1), 3.86 (s, CH_3, 3H, OCH_3), 3.83 (s, 3H, OCH_3), 3.18 (m, 1H, H4), 2.78 (m, 2H, H3) 1.26 (d, *J* = 6.6 Hz, 3H, H9); ^{13}C NMR (100 MHz, $CDCl_3$) δ 147.7 (C6/7), 147.4 (C6/7), 132.2 (C5a), 127.8 (C8a), 111.2 (C5), 108.9 (C8), 56.1 (OCH_3), 56.0 (OCH_3), 51.3 (C1), 48.6 (C3), 31.7 (C4), 20.8 (C9). HRMS (ESI+) calculated for $C_{12}H_{18}NO_2^+$ 208.1332, found 208.1333.

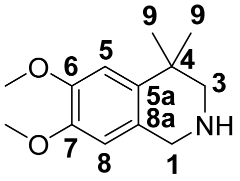

18 (26%)

6,7-dimethoxy-4,4-dimethyl-1,2,3,4-tetrahydroisoquinonline (**18**) was prepared from 2-(3,4-dimethoxyphenyl)-2-methylpropionitrile (**S17**) according to synthetic procedures 1 and 2, and isolated as am off-white oil (179 mg, 0.81 mmol, 26%). ^{1}H NMR (400 MHz, $CDCl_3$) δ 6.79 (s, 1H, H5/8), 6.46 (s, 1H, H5/8), 3.95 (s, 1H, H1), 3.86 (s, 3H OCH_3), 3.82 (s, 3H, OCH_3), 2.85 (s, 1H, H3), 1.26 (s, 6H, H9); ^{13}C NMR (100 MHz, $CDCl_3$) δ 147.9 (C6/7), 147.3 (C6/C7), 136.0 (C5a), 126.8 (C8a), 109.5 (C5), 108.6 (C8), 57.8 (C1), 56.2 (OCH_3), 56.0 (OCH_3), 48.9 (C3), 32.8 (C4), 29.3 (C9); HRMS (ESI+) calculated for $C_{13}H_{20}NO_2^+$ 222.1489, found 222.1491.

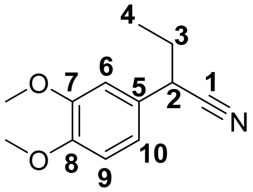

S18 (41%)

2-(3,4-Dimethoxyphenyl)butyronitrile (S18). Was prepared by adapting the procedure of Thiyagarajan *et al.*[8] as follows. Under a nitrogen atmosphere, Ru-MACHO® (274 mg, 0.45 mmol, 0.02 eq), potassium *tert*-butoxide (101 mg, 0.90 mmol, 0.04 eq), 2-(3,4-dimethoxyphenyl)acetonitrile (4 g, 22.6 mmol, 1 eq), ethanol (6.6 ml, 113 mmol, 5 eq) and toluene (34 mL) were sequentially added to a flame-dried round bottom flask and the mixture heated to reflux (110 °C) overnight. As TLC indicated the reaction was incomplete, further portions of potassium *tert*-butoxide (138 mg, 1.23 mmol, 0.05 eq) and ethanol (6.6 ml, 113 mmol, 5 eq) were added and the solution was heated to reflux (110 °C) for 3 hours. The reaction was cooled to room temperature and solvent removed under reduced pressure. The residue was dissolved in ethyl acetate and passed through a silica gel plug to remove the ruthenium catalyst, before being further purified by silica gel column chromatography (3:1 hexane:ethyl acetate). 2-(3,4-Dimethoxyphenyl)butyronitrile (**S18**) was isolated as a pale yellow oil (1.91 g, 9.3 mmol, 41%). ^{1}H NMR (400 MHz, $CDCl_3$) δ 6.92-6.72 (m, 3H, H9, H10 & H6), 3.90 (s, 3H, OCH_3), 3.88 (s, 3H, OCH_3), 3.68 (t, J = 7.2 Hz, 1H, H2), 1.92 (m, J = 7.3, 3.2 Hz, 2H, H3), 1.07 (t, J = 7.3 Hz, 3H, H4); ^{13}C NMR (100 MHz, $CDCl_3$) δ 149.4 (C7/8), 148.9 (C7/8), 128.3 (C5), 121.1 (C1), 119.7 (C10), 111.4 (C6), 110.3 (C9), 56.1 (OCH_3), 56.1 (OCH_3), 38.7 (C2), 29.4 (C3), 11.7 (C4); HRMS (ESI+) calculated for $[M-HCN]^+$ $C_{11}H_{15}O_2^+$ 179.1067, found 179.1067.

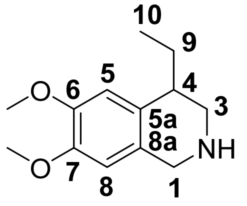

S3 (15%)

6,7-Dimethoxy-4-ethyl-1,2,3,4-tetrahydroisoquinoline (**S3**) was prepared from 2-(3,4-dimethoxyphenyl)butyronitrile (**S18**) according to synthetic procedures 1 and 2, and isolated as a pale yellow oil (15 %). ^{1}H NMR (400 MHz, $CDCl_3$) δ 6.70 (s, 1H, H5/8), 6.53 (s, 1H, H5/8), 4.18 (s, 1H, H1), 3.87 (s, 3H, OCH_3), 3.84 (s, 3H, OCH_3), 3.41 (dd, *J* = 12.9, 5.2 Hz, 1H, H3'), 3.15 (dd, *J* = 12.7, 5.2 Hz, 1H, H3"), 3.05-2.90 (m, 1H, H4 1.90 (m, 1H, H9'), 1.73 (m, 1H, H9"), 1.00 (t, *J* = 7.3 Hz, 3H, H10); ^{13}C NMR (100 MHz, $CDCl_3$) δ 148.8 (C6/7), 148.3 (C6/7), 128.1 (C5a), 120.3 (C8a), 110.5 (C5), 109.0 (C8), 56.1 (OCH_3), 56.1 (OCH_3), 44.5 (C1), 44.3 (C3), 35.9 (C4), 26.9 (C9), 11.3 (C10); HRMS (ESI+) calculated for $C_{13}H_{20}NO_2^+$ 222.1489, found 222.1491.

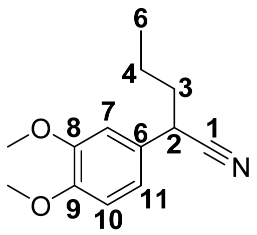

S19 (37%)

2-(3,4-Dimethoxyphenyl)pentanenitrile (S19). Under a nitrogen atmosphere, Ru-MACHO® (77 mg, 0.13 mmol, 0.006 eq), potassium *tert*-butoxide (27 mg, 0.24 mmol, 0.01 eq), 2-(3,4-dimethoxyphenyl)acetonitrile (4 g, 22.6 mmol, 1 eq), propan-1-ol (3.4 ml, 45 mmol,25 eq) and toluene (34 mL) were sequentially added to a flame-dried round bottom flask and the mixture heated to reflux (110 °C) overnight. As TLC indicated the reaction was incomplete, further portions of Ru-MACHO® (199 mg, 0.33 mmol, 0.015 eq), potassium *tert*-butoxide (80 mg, 0.72 mmol, 0.03 eq) and propan-1-ol (5.1 ml, 68 mmol, 3 eq) were added and the solution was heated to reflux (110 °C) for 3 hours. The reaction was cooled to room temperature and solvent removed under reduced pressure. The residue was dissolved in ethyl acetate and passed through a silica gel plug to remove the ruthenium catalyst, before being further purified by silica gel column chromatography (3:1 hexane:ethyl acetate). 2-(3,4-Dimethoxyphenyl)pentanenitrile (**S19**) was isolated as a pale yellow oil (1.83 g, 8.36 mmol, 37%). ^{1}H NMR (500 MHz, $CDCl_3$) δ 6.86-6.84 (m, 2H, H7 & H11), 6.81 (d, *J* = 1.7 Hz, 1H, H10), 3.90 (s. 3H OCH_3), 3.88 (s, 3H, OCH_3), 3.72 (dd, *J* = 8.6, 6.3 Hz, 1H, H2), 1.98 – 1.76 (m, 2H, H3), 1.58 – 1.39 (m, 2H, H4), 0.96 (t, J = 7.4 Hz, 3H, H5); ^{13}C NMR (125 MHz, $CDCl_3$) δ 149.4 (C8/9), 148.8 (C8/9), 128.5 (C6), 121.3 (C1), 119.7 (C11), 111.4 (C7), 110.3 (C10), 56.1 (OCH_3), 56.1 (OCH_3), 38.1 (C2), 36.9 (C3), 20.5 (C4), 13.6 (C5); HRMS (ESI+) calculated for $[M-HCN]^+$ 193.1223, found 193.1218

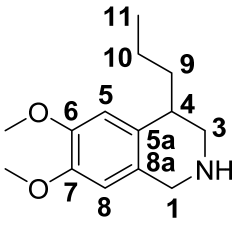

S4 (23%)

6,7-Dimethoxy-4-propyl-1,2,3,4-tetrahydroisoquinoline (**S4**) was prepared from 2-(3,4-dimethoxyphenyl)pentanenitrile (**S19**) according to synthetic procedures 1 and 2, and isolated as a pale yellow oil (72 mg, 0.30 mmol, 23%). ^{1}H NMR (400 MHz, $CDCl_3$) δ 6.69 (s, 1H, H5/8), 6.53 (s, 1H, H5/8), 4.16 (s, 1H, H1), 3.87 (s, 3H, OCH_3), 3.84 (s, 3H, OCH_3), 3.39 (dd, *J* = 12.6, 5.1 Hz, 1H, H3'), 3.11 (dd, *J* = 12.5, 6.8 Hz, 1H, H3"), 3.03 (m, J = 5.0 Hz, 1H, H4), 1.79 (m, 1H, H9'), 1.66 (m, 1H, H9"), 1.56 – 1.31 (m, 2H, H10), 0.97 (t, J = 7.3 Hz, 3H, H11); ^{13}C NMR (100 MHz, $CDCl_3$) δ 148.8 (C6/7), 148.2 (C6/7), 128.6 (C5a), 120.5 (C8a), 110.5 (C5), 109.0 (C8), 56.1 (OCH_3), 56.1 (OCH_3), 44.9 (C1), 44.3 (C3), 36.4 (C4), 34.4 (C9), 20.1 (C10), 14.3 (C11); HRMS (ESI+) calculated for $C_{14}H_{22}NO_2^+$ 236.1645, found 236.1642.

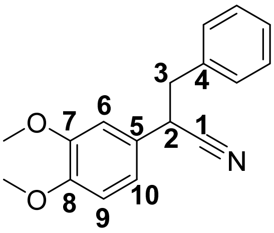

S20 (29%)

2-(3,4-dimethoxyphenyl)-3-phenylpropanenitrile (S20). Under a nitrogen atmosphere, Ru-MACHO® (72 mg, 0.12 mmol, 0.005 eq), potassium *tert*-butoxide (25 mg, 0.22 mmol, 0.14 eq), 2-(3,4-dimethoxyphenyl)acetonitrile (4 g, 22.6 mmol, 1 eq), benzyl alcohol (5.0 ml, 48 mmol, 2 eq) and toluene (34 mL) were sequentially added to a flame-dried round bottom flask and the mixture heated to reflux (110 °C) overnight. As TLC indicated the reaction was incomplete, further portions of Ru-MACHO® (187 mg, 0.31 mmol, 0.014 eq), potassium *tert*-butoxide (145 mg, 1.29 mmol, 0.06 eq) and benzyl alcohol (7.0 ml, 68 mmol, 3 eq) were added and the solution was heated to reflux (110 °C) for 3 hours. The reaction was cooled to room temperature and solvent removed under reduced pressure. The residue was dissolved in ethyl acetate and passed through a silica gel plug to remove the ruthenium catalyst, before being further purified by silica gel column chromatography (4:1 hexane:ethyl acetate). 2-(3,4-Dimethoxyphenyl)3-phenylpropanenitrile (**S20**) was isolated as a pale yellow oil that slowly crystallised on standing (1.77 g, 6.6 mmol, 29%). ^{1}H NMR (400 MHz, $CDCl_3$) δ 7.33-7.21 (m, 3H, Ph), 7.12 (dd, *J* = 7.7, 1.8 Hz, 2H, Ph), 6.83-6.80 (m, 2H, H9 & H10), 6.64 (d, *J* = 1.8 Hz, 1H, H6), 3.95 (d, *J* = 12.9 Hz, 1H, H2), 3.88 (s, 3H, OCH_3), 3.80 (s, 3H, OCH_3), 3.26-3.06 (m, 2H, H3); ^{13}C NMR (100 MHz, $CDCl_3$) δ 149.3 (C7/8), 149.0 (C7/8), 136.4 (C4), 129.5 (Ph), 128.7 (Ph), 127.6 (C5), 127.5 (Ph), 120.7 (C1), 119.9 (C9), 111.4 (C10), 110.7 (C6), 56.1 (OCH_3), 56.1 (OCH_3), 42.4 (C3), 39.4 (C2); HRMS (ESI$^+$) calculated for $[M-HCN]^+$ $C_{16}H_{17}O_2^+$ 241.1223 found 241.1224.

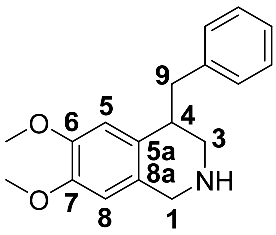

S5 (50%)

4-benzyl-6,7-dimethoxy-1,2,3,4-tetrahydroisoquinoline (**S5**) was prepared from 2-(3,4-dimethoxyphenyl)-3-phenylpropanenitrile (**S20**) according to synthetic procedures 1 and 3, and isolated as a pale yellow oil that slowly crystallised on standing (50 %). ^{1}H NMR (400 MHz, $CDCl_3$) δ 7.36-7.13 (m, 5H, Ph) 6.51 (s, 1H, H5/8), 6.45 (s, 1H, H5/8), 3.94 (s, 2H, H1), 3.84 (s, 3H, OCH_3), 3.73 (2 x s, 3H, OCH_3), 3.09-2.82 (m, 5H, H3, H4 & H9); ^{13}C NMR (100 MHz, $CDCl_3$) δ 147.4 (C6/7), 147.1 (C6/7), 140.4 (Ph), 130.0a (C5a), 129.4, 128.4 (Ph), 128.3 (C8a), 126.1 (Ph), 111.9 (C5), 108.7 (C8), 55.9 (OCH_3), 55.8 (OCH_3), 48.2 (C1), 47.6 (C3), 42.3 (C9), 38.7 (C4); HRMS (ESI+) calculated for $C_{18}H_{22}NO_2^+$ 284.1645; found 284.1633.

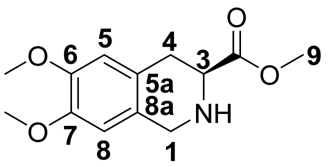

S7 (86%)

Methyl (S)-6,7-dimethoxy-1,2,3,4-tetrahydroisoquinoline-3-carboxylate (S7).[S9] Under a nitrogen atmosphere, the hydrochloride salt of (*S*)-6,7-dimethoxy-1,2,3,4-tetrahydroisoquinoline-3-carboxylic acid (**S6**, 2.74 g, 10 mmol) was dissolved in anhydrous methanol (100 mL) and cooled to 0 °C using an ice bath. Thionyl chloride (1.45 mL, 20 mmol, 2 eq) was added dropwise, after which the reaction was heated to reflux for 24 h. Toluene (25 mL) was added and solvent was removed under reduced pressure. The residue was redissolved in dichloromethane (150 mL) and washed with a saturated solution of sodium bicarbonate (2 x 100 mL) and brine (100 mL). The organic phase was dried over magnesium sulfate and solvent removed under reduced pressure. The residue was redissolved in ethyl acetate (50 mL) and passed through a plug of silica gel. Solvent was removed under reduced pressure to yield methyl (*S*)-6,7-dimethoxy-1,2,3,4-tetrahydroisoquinoline-3-carboxylate (**S7**) as a pale yellow solid (2.17 g, 8.6 mmol, 86%). A sample of material was treated with hydrochloric acid (0.1 M) and solvent removed under reduced pressure to afford the hydrochloride salt for analysis. ^{1}H NMR (400 MHz, D_2O) δ 6.79 (s, 1H, H5/8), 6.75 (s, 1H, H5/8), 4.33 (m, 3H, H1 & H3), 3.83 (s, 3H, H9), 3.73 (s, 3H, OCH_3), 3.72 (s, 3H, OCH_3), 3.27 (dd, J = 17.3, 5.5 Hz, 1H, H4), 3.11 (dd, J = 17.3, 11.0 Hz, 1H, H4'); ^{13}C NMR (100 MHz, D_2O) δ 169.7 (C=O), 148.0 (C6/7), 147.5 (C6/7), 122.3 (C5a), 119.1 (C8a), 111.5 (C5), 109.2 (C8), 55.7 (OCH_3), 55.6 (OCH_3), 54.2 (C9), 48.3 (C3), 27.3 (C1), 23.8 (C4); HRMS (ESI+) calculated for $C_{13}H_{18}NO_4^+$ 252.1230, found 252.1236.

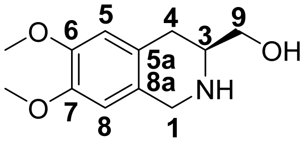

S8 (52%)

(*S*)-6,7-Dimethoxy-3-hydroxymethyl-1,2,3,4-tetrahydroisoquinoline (S8).[S10] Under a nitrogen atmosphere, lithium aluminium hydride (1 M solution in THF, 4.4 mL, 4.4 mmol, 1.1 eq) was added to a flame-dried flask and cooled to 0 °C using an ice bath. A solution of methyl (*S*)-6,7-dimethoxy-1,2,3,4-tetrahydroisoquinoline-3-carboxylate (**S7**, 1005 mg, 4 mmol) in THF (5.6 mL) was added dropwise, after which the solution was warmed to room temperature overnight. The solution was cooled in an ice bath and quenched by the sequential, dropwise addition of water (180 µL), sodium hydroxide solution (0.5 M, 130 µL) and water (620 µL).[S3] The supernatant was decanted and filtered, and the solid residue was washed with tetrahydrofuran (3 x 50 mL). The THF washes were combined and solvent removed under reduced pressure. The residue was redissolved in dichloromethane (200 mL) and washed with a saturated solution of sodium bicarbonate (2 x 100 mL) and brine (100 mL). The combined aqueous phases were adjusted to pH >12 using sodium hydroxide, saturated with sodium chloride, and extracted with dichloromethane (4 x 100 mL). Solvent was removed from the combined organic phases to yield (*S*)-6,7-dimethoxy-3-hydroxymethyl-1,2,3,4-tetrahydroisoquinoline (**S8**) as an off-white solid (347 mg, 1.6 mmol, 52%). ^{1}H NMR (400 MHz, $CDCl_3$) δ 6.57 (s, 1H, H5/8), 6.52 (s, 1H, H5/8), 3.98 (s, 2H, H1), 3.84 (s, 3H, OCH_3), 3.84 (s, 3H, OCH_3), 3.77 (dd, *J* = 10.8, 3.9 Hz, 1H, H9), 3.50 (dd, *J* = 10.8, 8.3, 1H, H9’), 3.05 (m, 1H, H3), 2.62 (dd, *J* = 16.1, 4.4 Hz, 1H, H4), 2.48 (dd, *J* = 16.1, 10.6 Hz, 1H, H4’); ^{13}C NMR (100 MHz, $CDCl_3$) δ 147.6 (C6/7), 147.4 (C6/7), 127.7 (C5a), 125.7 (C8a), 112.0 (C5), 108.9 (C8), 66.0 (C9), 55.9 (OCH_3), 55.9 (OCH_3), 55.2 (C3), 47.6 (C1), 30.5 (C4); HRMS (ESI+) calculated for $C_{12}H_{18}NO_3^+$ 224.1281, found 224.1283.

Supplementary references

1 S. Panjikar, V. Parthasarathy, V. S. Lamzin, M. S. Weiss and P. A. Tucker, *Acta Crystallogr. Sect. D Biol. Crystallogr.*, 2009, **65**, 1089–1097.

2 T. C. Terwilliger, *Acta Crystallogr. Sect. D Biol. Crystallogr.*, 2000, **56**, 965–972.

3 L. H. Amundsen and L. S. Nelson, *J. Am. Chem. Soc.*, 1951, **73**, 242–244.

4 J. S. Buck, R. D. Haworth and W. H. Perkin, *J. Chem. Soc., Trans.*, 1924, **125**, 2176–2185.

5 A. Aditya, D. E. Nichols and G. M. Loudon, *J. Chem. Educ.*, 2008, **85**, 1535.

6 B. E. Blough and F. I. Carroll, *Tetrahedron Lett.*, 1993, **34**, 7239–7242.

7 J. Guin, G. Varseev and B. List, *J. Am. Chem. Soc.*, 2013, **135**, 2100–2103.

8 S. Thiyagarajan and C. Gunanathan, *ACS Catal.*, 2017, **7**, 5483–5490.

9 S. Aubry, S. Pellet-Rostaing, R. Faure and M. Lemaire, *J. Heterocycl. Chem.*, 2006, **43**, 139–148.

10 C. Kang, Z. Bian, Y. He, F. Han, X. Qiu and L. Gao, *Chem. Commun.*, 2011, **47**, 10746.

11 J. L. Vicario, D. Badía, E. Domínguez and L. Carrillo, *Tetrahedron Asymmetry*, 2000, **11**, 3779–3788.

12 A. S. Kumar, S. Ghosh, R. Soundararajan and G. N. Mehta, *Arkivoc*, 2009, **2009**, 173–180.

13 S. Venugopal, J. Ramantham, N. Devanna, A. S. Kumar and S. Ghosh, *Asian J. Chem.*, 2010, **22**, 1835–1840.

14 D. Seebach, V. Ehrig, H. F. Leitz and R. Henning, *Chem. Ber.*, 1975, **108**, 1946–1960.

15 R. J. Worthington, N. M. Bell, R. Wong and J. Micklefield, *Org. Biomol. Chem.*, 2008, **6**, 92–103.

16 J. E. Nordlander, M. J. Payne, F. G. Njoroge, M. A. Balk, G. D. Laikos and V. M. Vishwanath, *J. Org. Chem.*, 1984, **49**, 4107–4111.

17 F. Werner, N. Blank and T. Opatz, *European J. Org. Chem.*, 2007, 3911–3915.

A

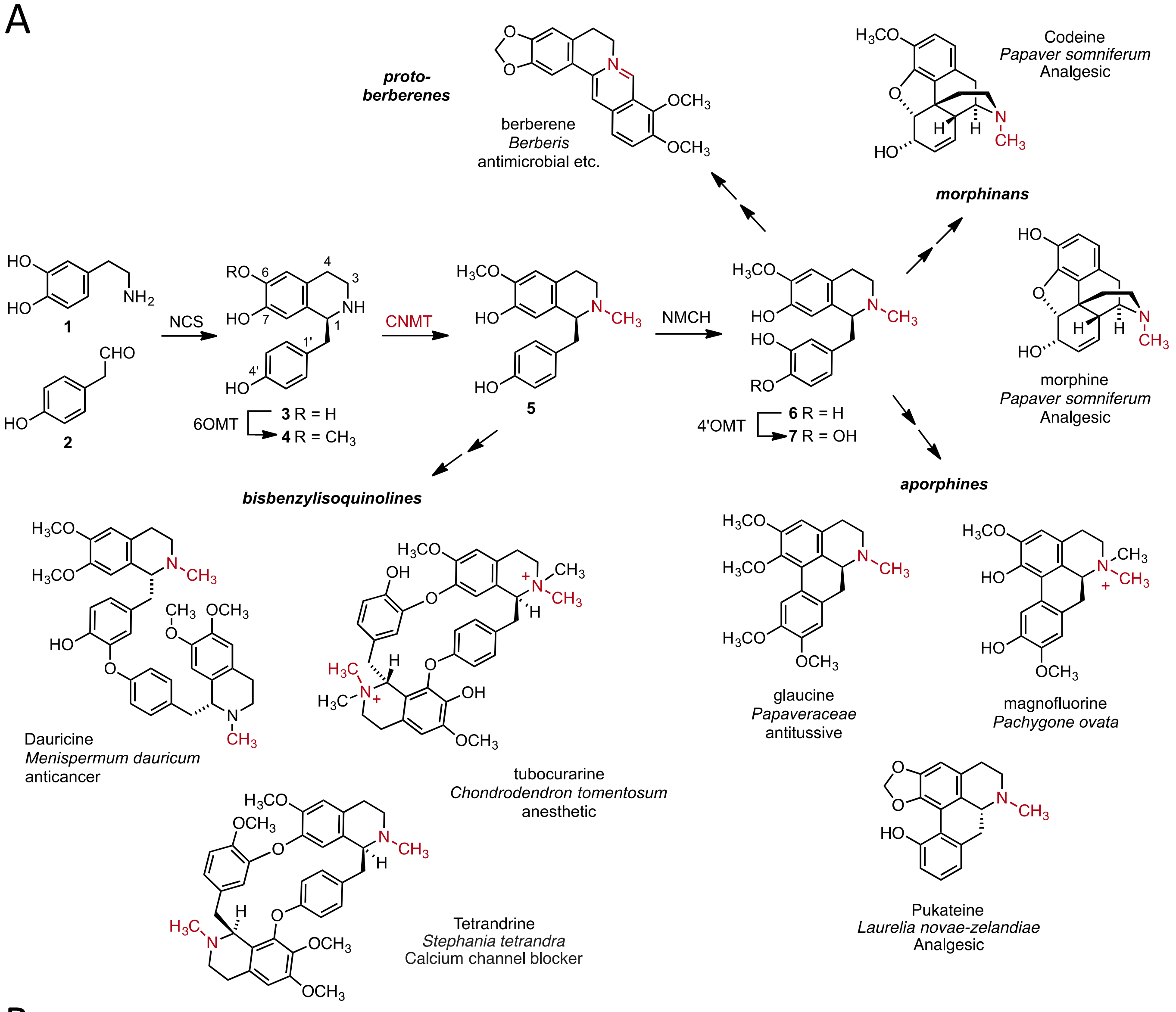

B

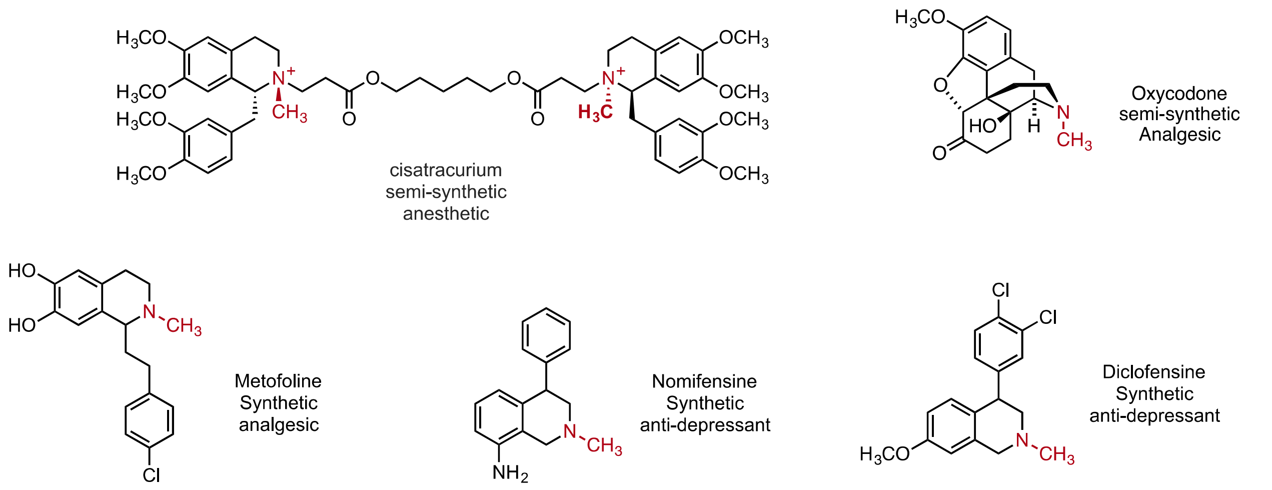

Figure S1. Examples of *N*-methylated tetrahydroisoquinolines that have been developed as drugs or that possess important biological activity including (**A**) plant derived BIA natural products and (**B**) Semi-synthetic and synthetic derivatives.

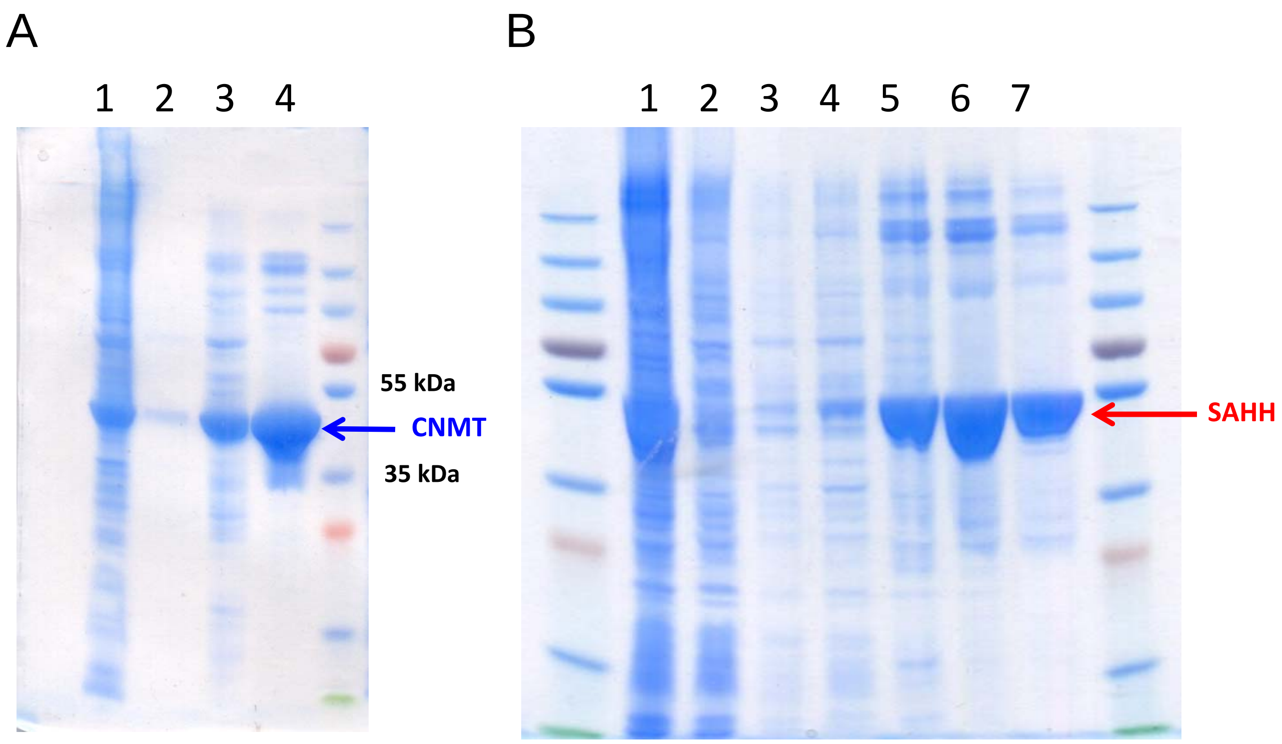

Figure S2. (A) SDS PAGE of coclaurine-*N*-methyltransferase (CNMT from *Coptis Japonica*) expression and purification. Lane 1 Soluble cell extract, Lane 2 Flow through, Lane 3 60 mM imidazole wash, Lane 4 500 mM imidazole elution. **(B)** SDS PAGE of *S*-adenosylhomocysteine hydrolase (from *Rattus norvegicus*) Ladder, Lane 1 Soluble cell extract, Lane 2 Flow through, Lane 3 10 mM imidazole wash, Lane 4 60 mM imidazole wash, Lane 5 90 mM imidazole wash, Lane 6 250 mM imidazole elution, Lane 7 500 mM imidazole elution, Ladder.

	CNMT, SeMeT, AdoHcy (PDB xxx)	CNMT, AdoHcy, **8a** (PDB xxx)	CNMT, AdoHcy, **9** (PDB xxx)
Data collection	SAD		
Wavelength (λ)	0.9795	1.00	1.00
Space group	$P2_12_12_1$	$P2_12_12_1$	$P2_12_12$
Unit cells			
a, b, c (Å)	141.75, 203.43, 54.54	48.47, 96.05, 154.6	48.42, 95.98, 154.52
a, b, g (°)	90.0, 90.0, 90.0	90.0, 90.0, 90.0	90.0, 90.0, 90.0
Res. (Å)	2.43-66.9 (67.8-2.4)	2.35-46.2 (2.41-2.35)	2.85-50.0 (3.02-2.85)
R_{merge}	0.06 (1.157)	0.12 (1.11)	0.09 (0.74)
I / sI	17.7 (4.3)	13.5 (1.5)	14.5 (2.08)
$CC_{1/2}$	0.9	0.6	0.88
Completeness	99.7 (99)	99.9 (99.8)	99.2 (98.5)
Anomalous Completeness	99.7 (99.1)		
Redundancy	18.4	6.4	4.9
Anomalous Redundancy	9.6 (9.8)		
Anomalous Slope	1.261		
dF/F	0.084		
dls(dl)	1.118		
Refinement			
Res. (Å)	2.4	2.35	2.85
No. reflections	60299	30922	17366
R_{work} / R_{free}	0.16/0.24	0.20/0.26	0.22/0.29
No. atoms	11596	5848	5869
B-factors	39.5	65.6	59.7
R.m.s.d.			
Bond lengths (Å)	0.008	0.008	0.009

Table S1 X-ray crystalography data and refinement statistics for the three CNMT structures (PDBxxx, PDBxxx & PDBxxx)

**Pocket feature**	**CNMT**	**pavNMT**
Volume	405.7 $Å^3$	934.9 $Å^3$
Area	387.8 $Å^2$	1081.2 $Å^2$
Hydrophobicity	0.7319	0.5093
Buriedness	0.8877	0.7245
DLID	0.8928	0.3613
Radius	4.592	6.065
Nonspericity	1.463	2.338

Table S2 Comparison of the active sites of CNMT and pavNMT (PDBxxx)

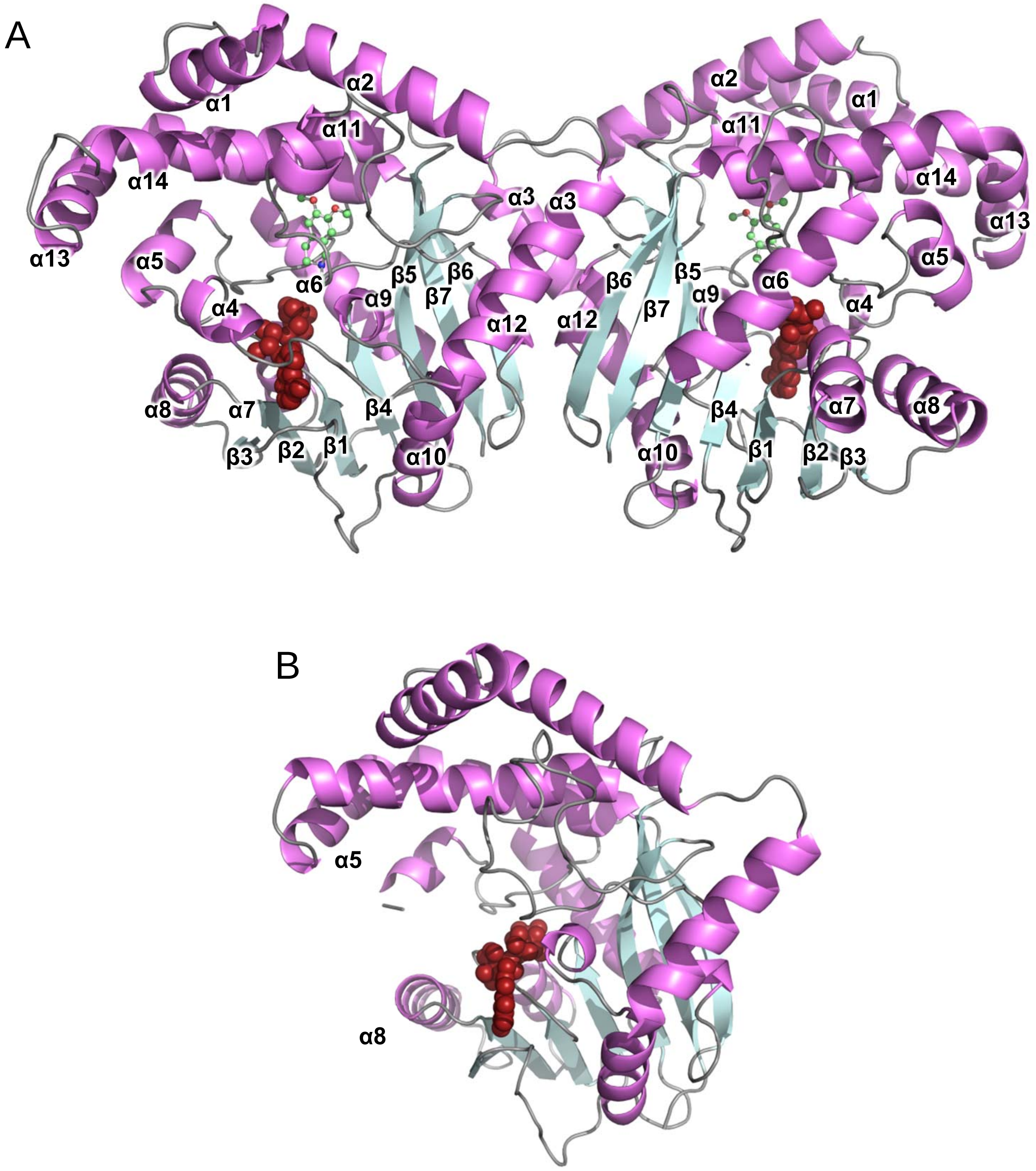

Figure S3. (A) The structure of the holo CNMT dimer (PDBxxx) in complex with AdoHyc (megenta space filling model) and the product *N*-methylheliamine (**8a**) (green ball and stick) showing the Helix α4, close to the substrate binding site, forming a closed cap over the substrate entrance. **(B)** The structure of the apo CNMT dimer (PDBxxx) with with AdoHyc, but no substrate, bound (only one sub-unit is show). The helix α4 is disordered (missing), which suggests that helix α4 is inherent flexibility, and controls substrate accessibility and/or substrate recognition by CNMT.

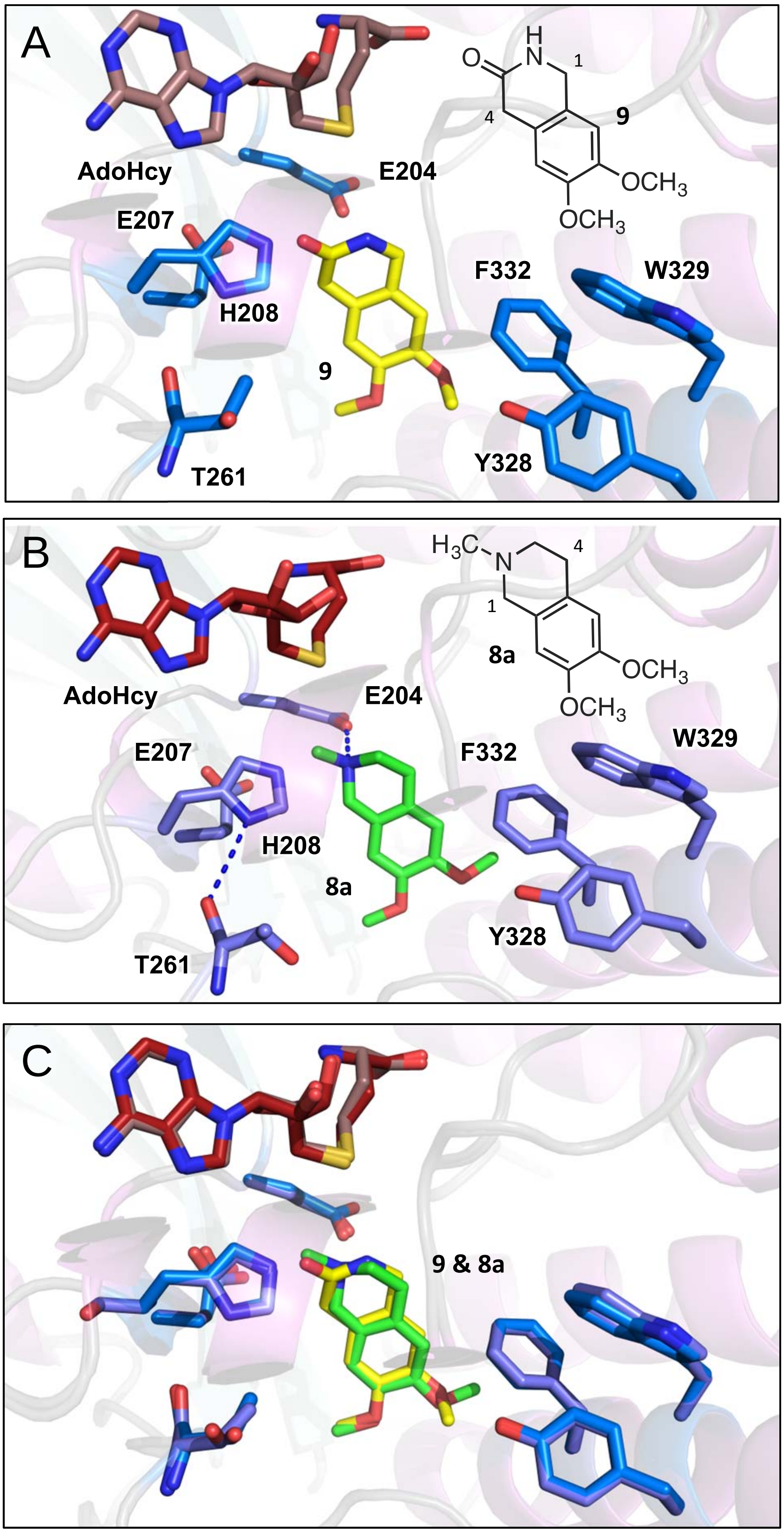

Figure S4. X-ray structures of CNMT in complex with (A) the substrate analog quinolinone **9** (B) the product heliamine **8a**. (C) Shows the overlay of the two structure. Note that **9** is flipped relative to **8a**

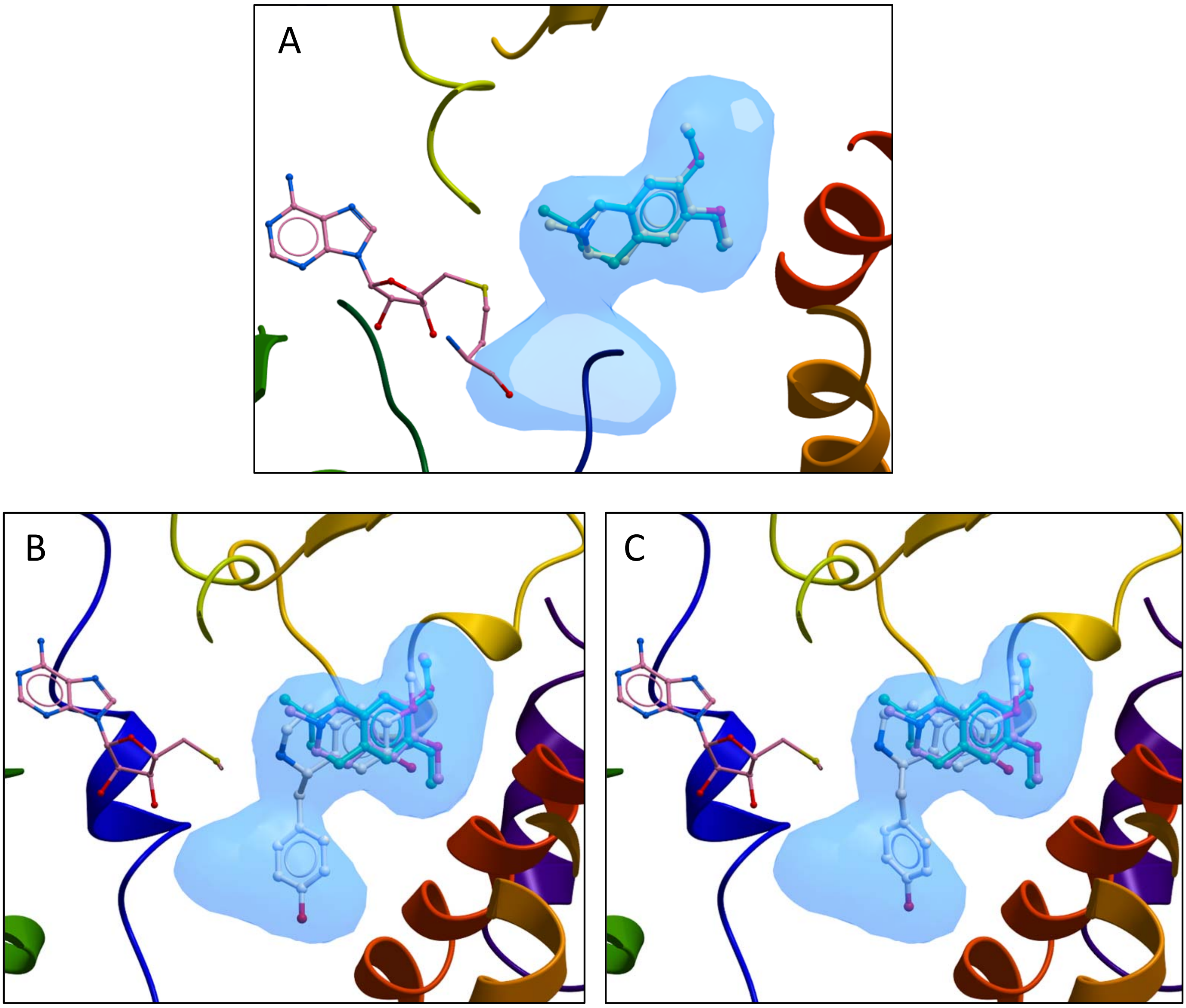

Figure S5. Docking of Coclaurine in the active site of cjCNMT. **(A)** To verify the modelling and obtain a relative docking score *N*-methylheliamine (**8a**) was removed from the structure prior to pocket calculations and re-docking. Docked ligand (Cyan) overlaid with the crystal structure (grey). Docking score e = –15.76 kcal/mol. **(B)** The lowest energy structure of (S)-coclaurine (**4**) docked in the active site has a docking score e = – 19.39 kcal/mol. **(C)** The lowest energy structure of (R)-enantiomer of coclaurine, which has a docking score of e = –9.98 kcal/mol.

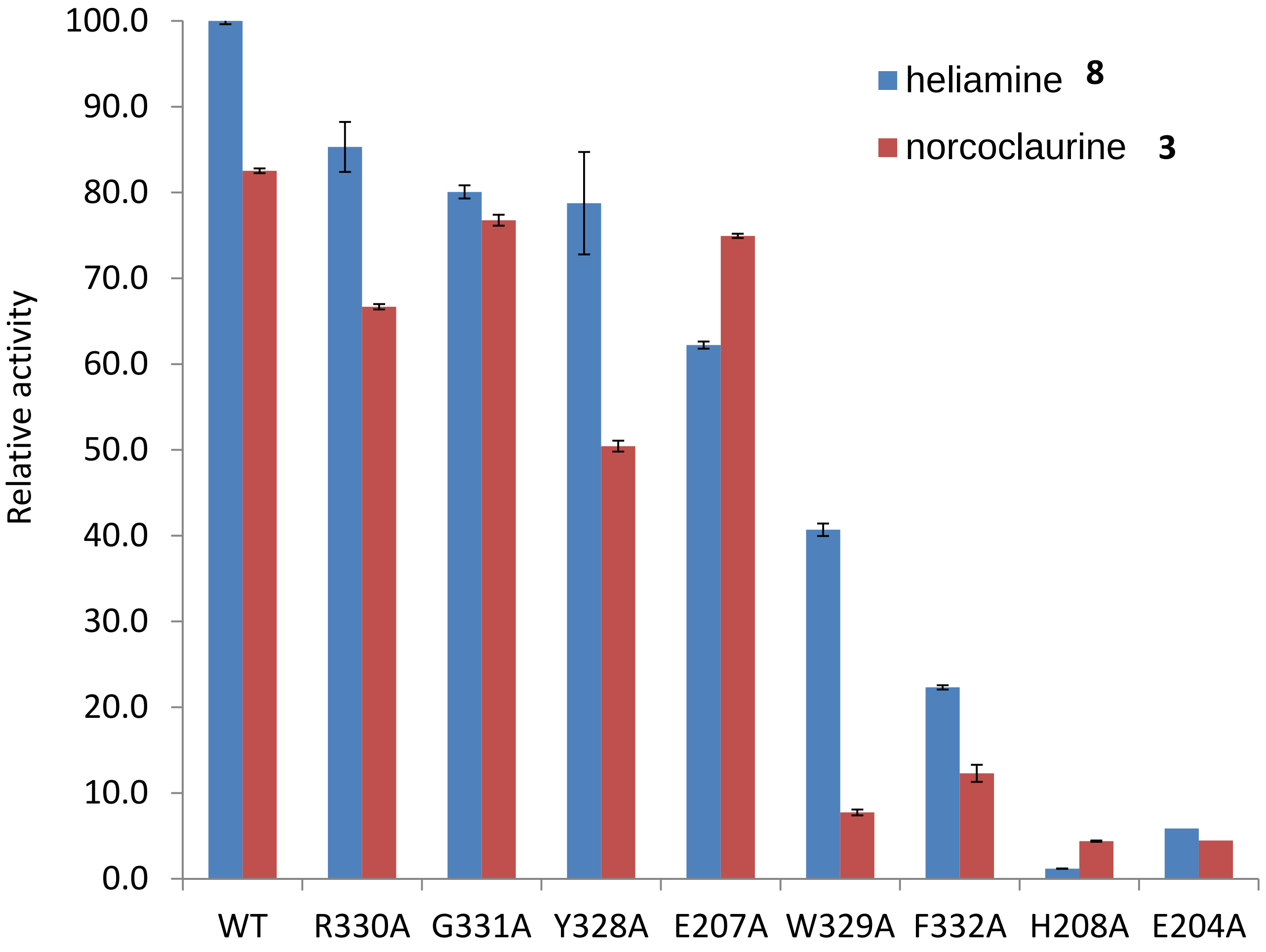

Figure S6. Relative activity of CNMT active site mutants.

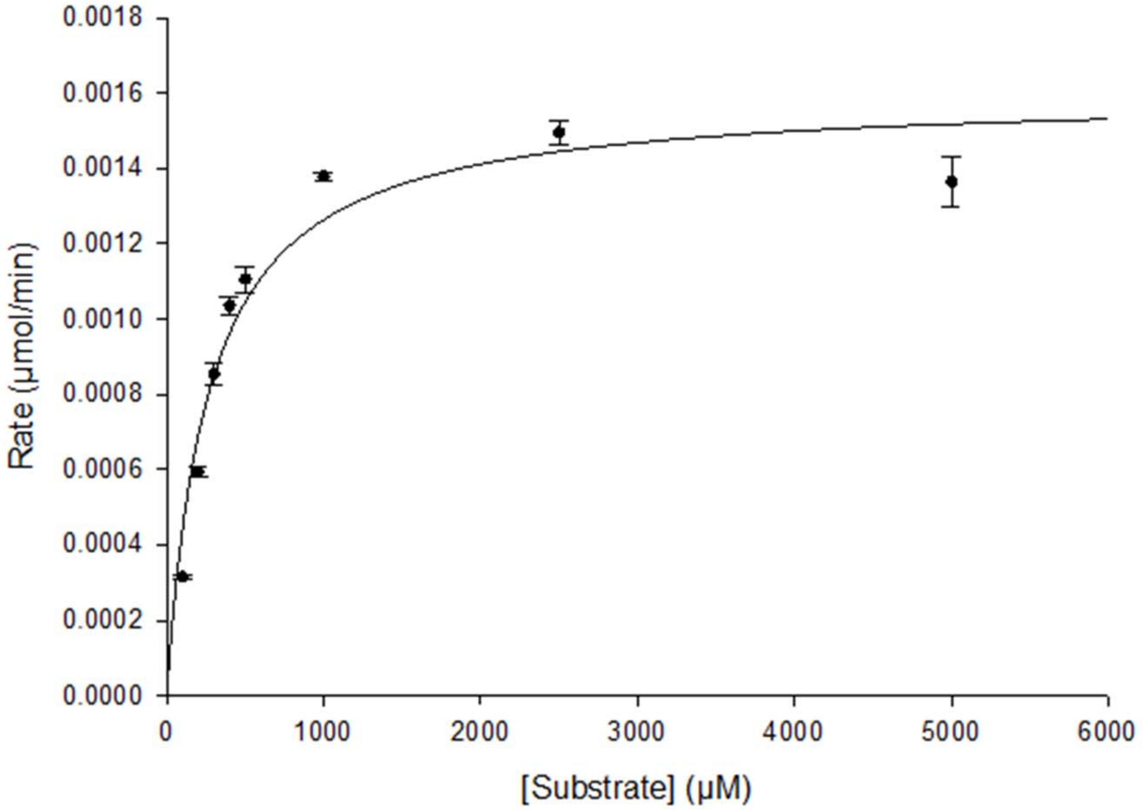

Figure S7. Michaelis-Menten curve for WT CNMT with norcoclaurine **3** as a substrate.

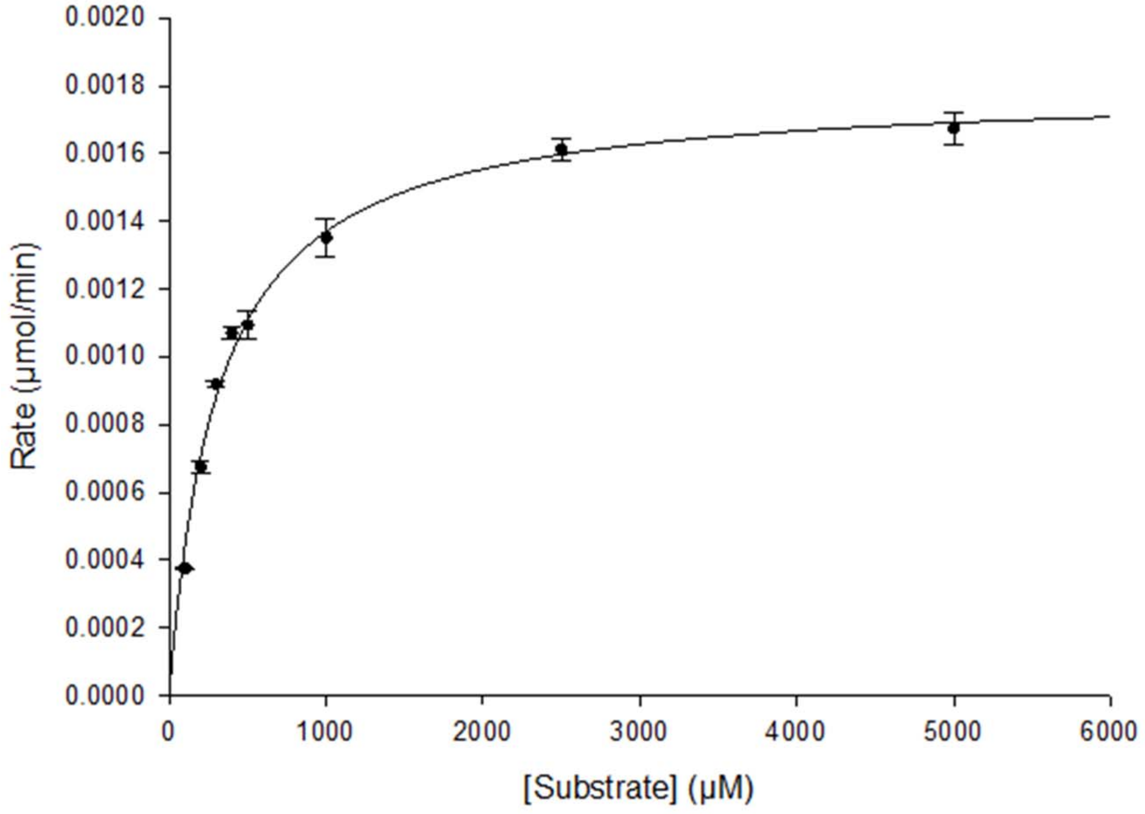

Figure S8. Michaelis-Menten curve for WT CNMT with heliamine **8** as a substrate.

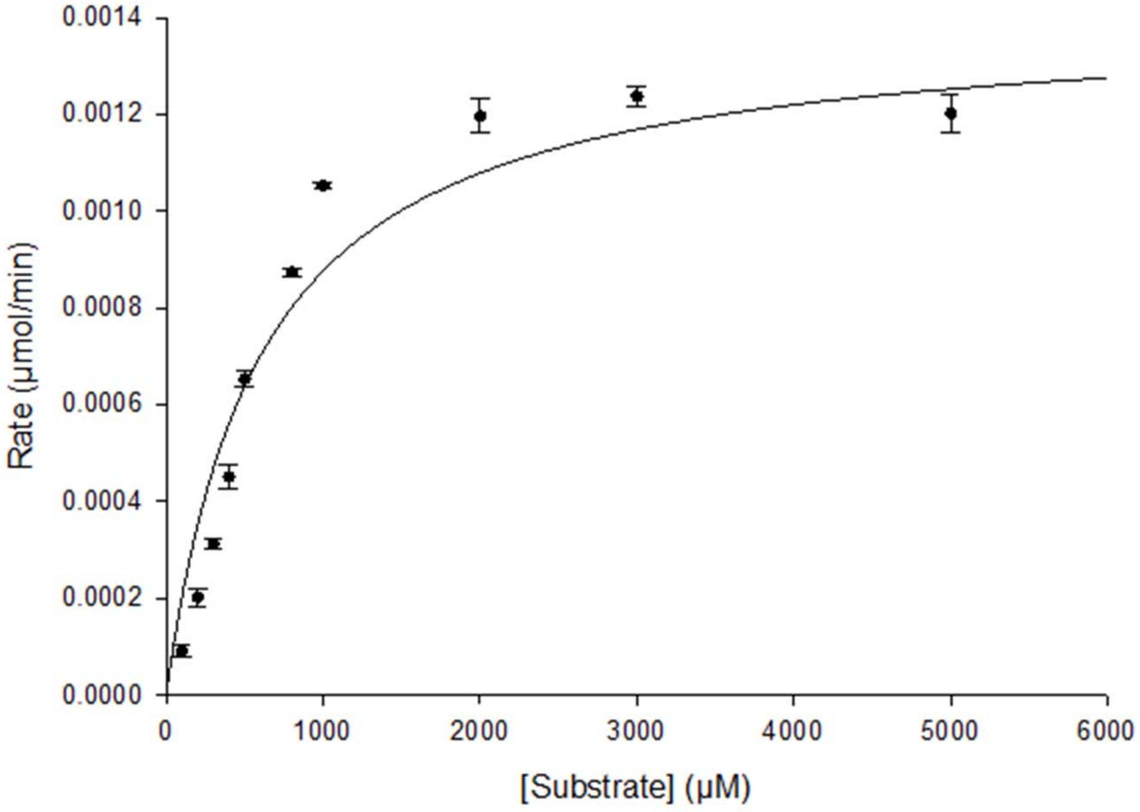

Figure S9. Michaelis-Menten curve for Y328A CNMT with norcoclaurine **3** as a substrate.

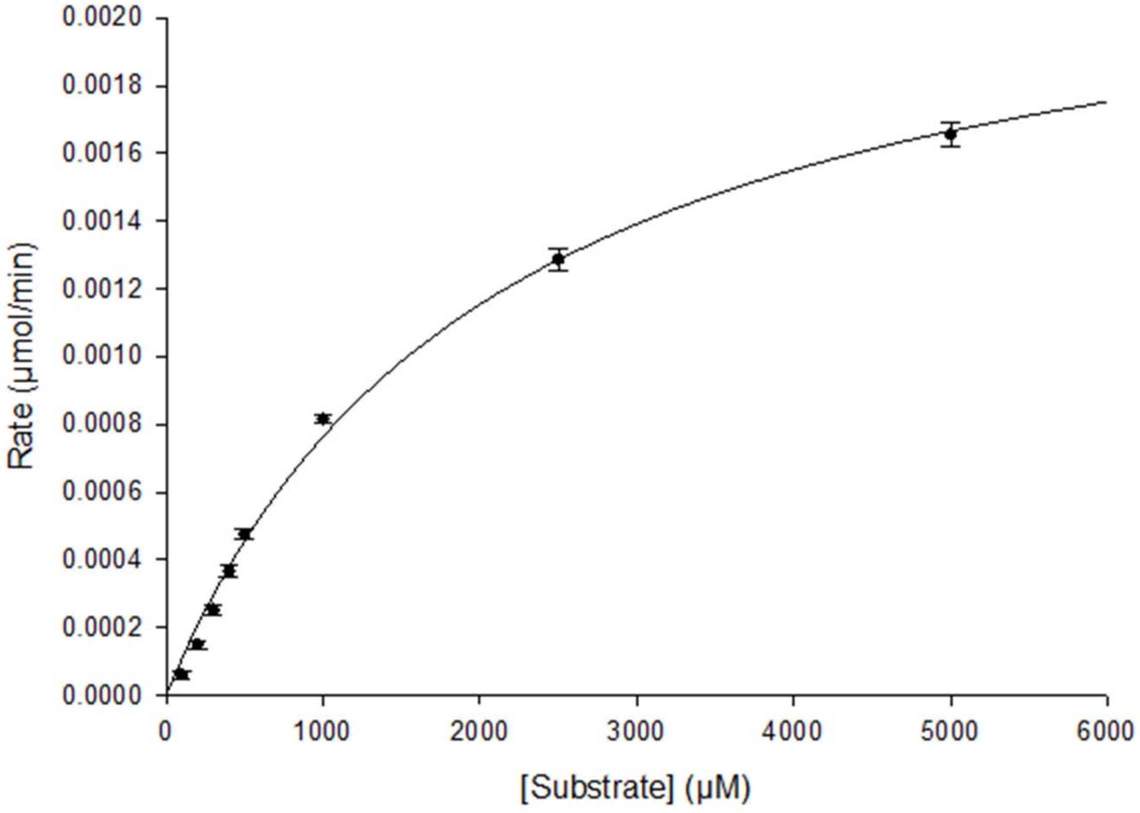

Figure S10. Michaelis-Menten curve for Y328A CNMT with heliamine **8** as a substrate.

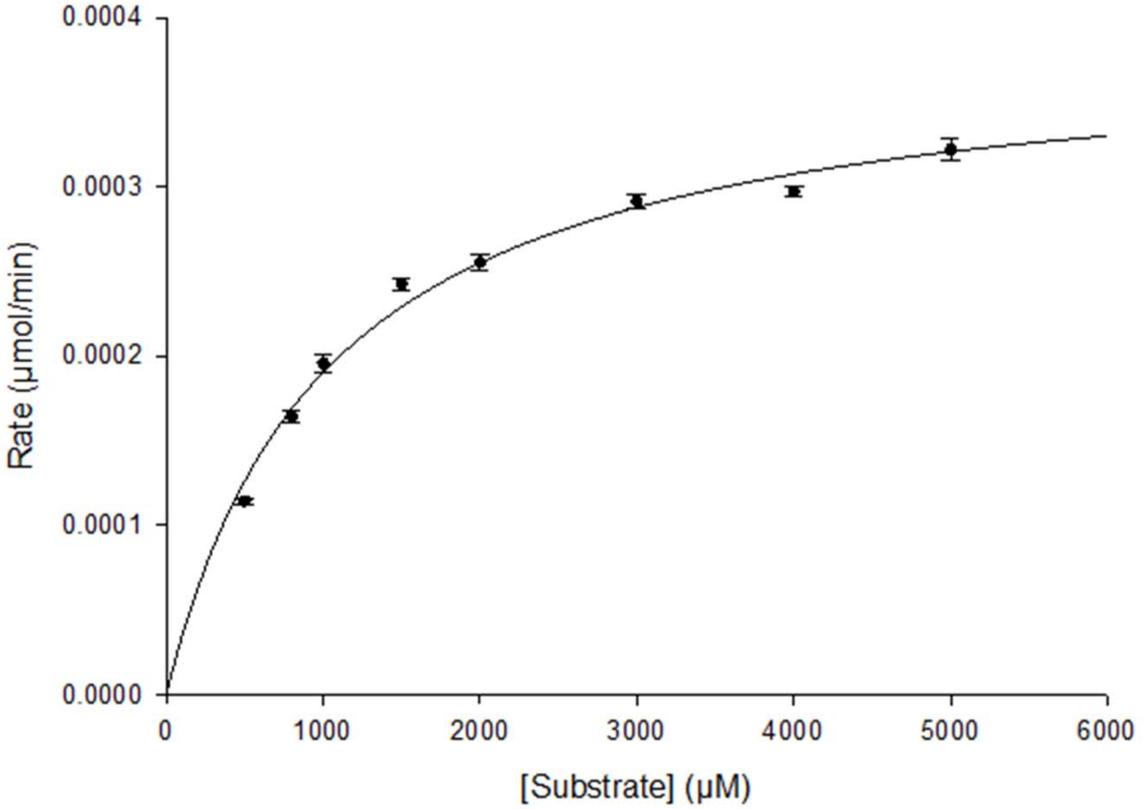

Figure S11. Michaelis-Menten curve for W329A CNMT with norcoclaurine **3** as a substrate.

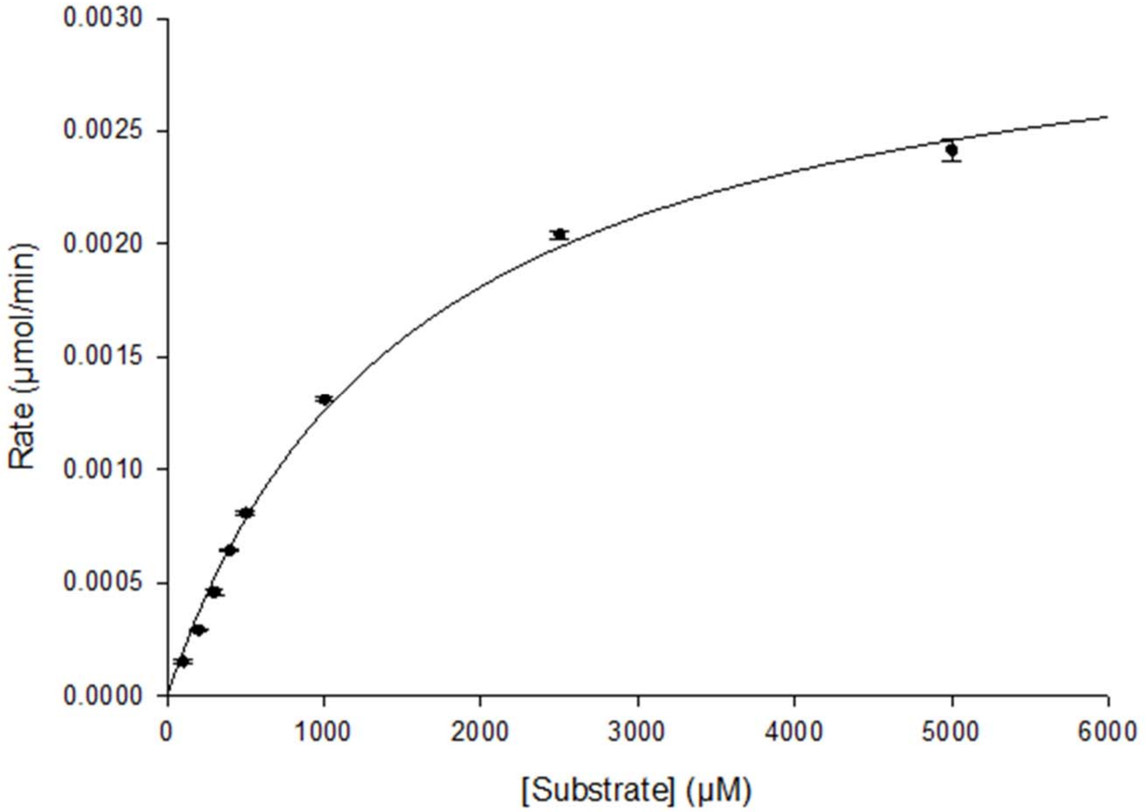

Figure S12. Michaelis-Menten curve for W329A CNMT with heliamine **8** as a substrate.

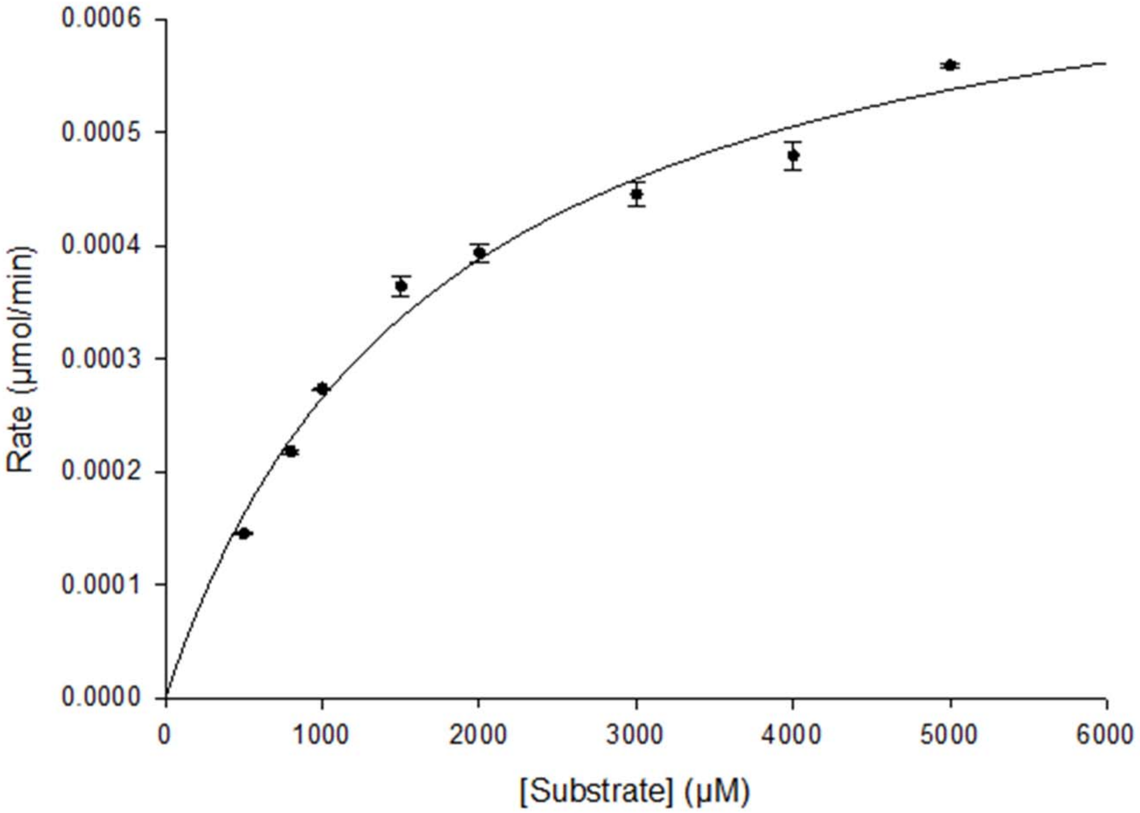

Figure S13. Michaelis-Menten curve for F332A CNMT with norcoclaurine **3** as a substrate.

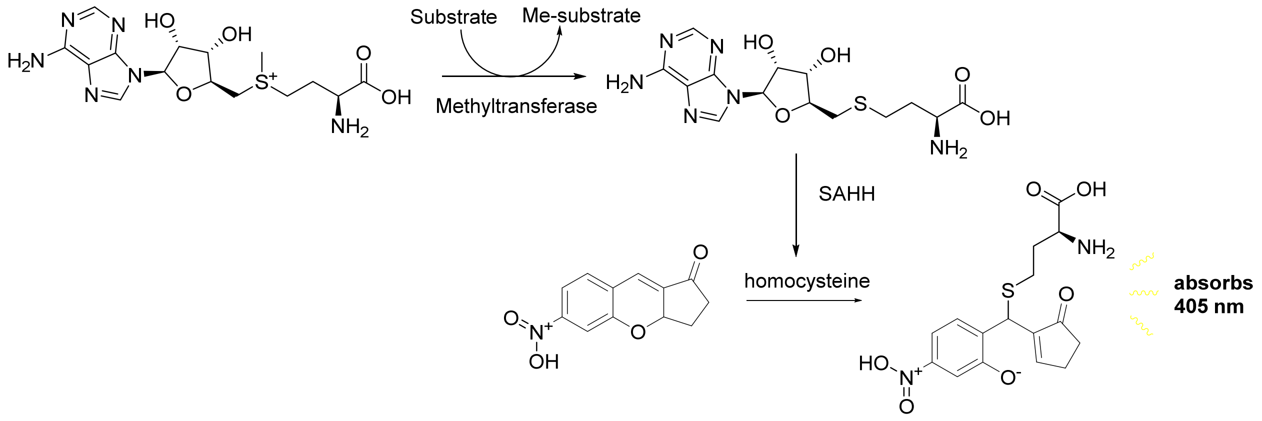

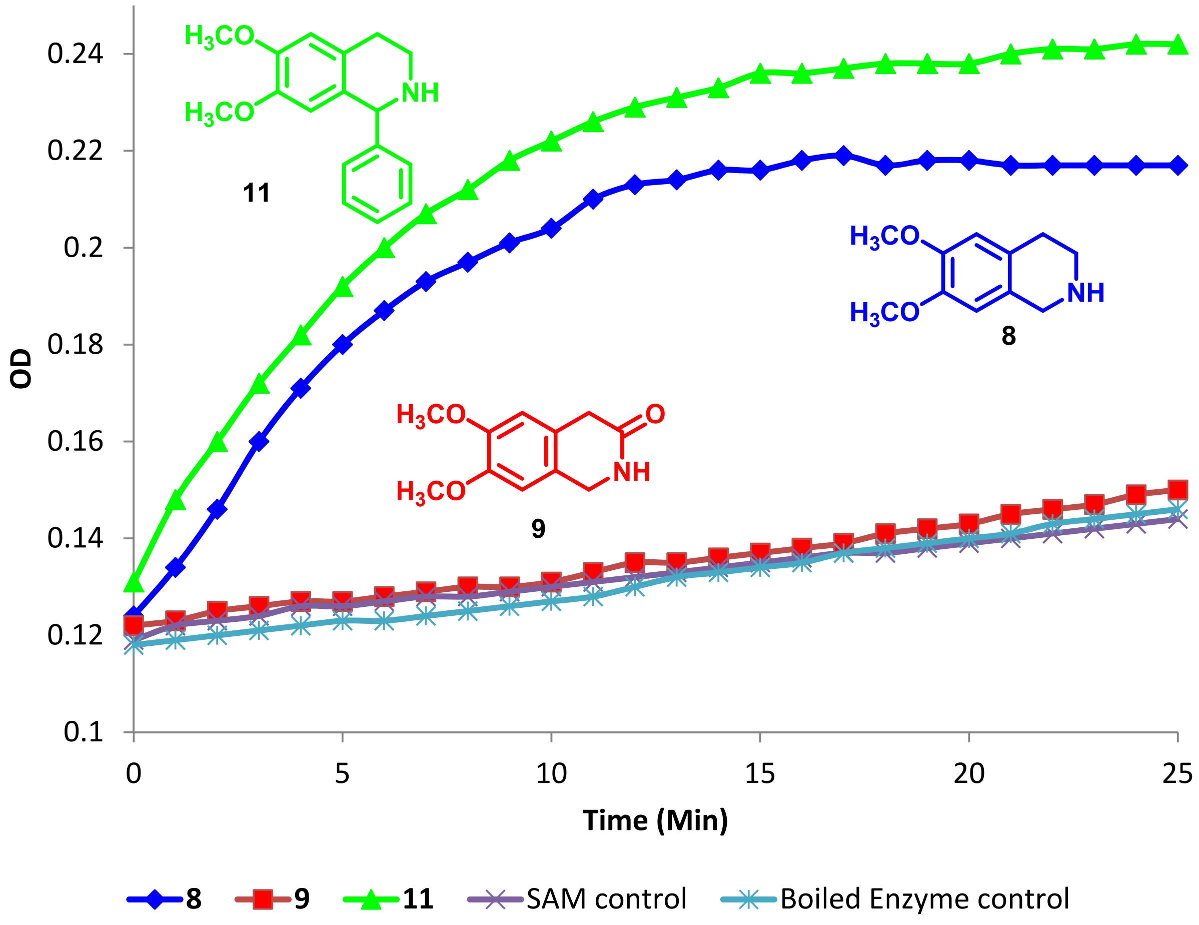

Figure S14. (A) Scheme showing compounds utilised during colorimetric assay for determination of Mtase activity. **(B)** Example of colorimetric assay raw data result (80 µM AdoMet, 80 µM substrate, 5 µM CNMT, 25 µM NDCC probe and 1 µM SAHH enzyme). This is aqualitative assay with active substrates showing a clear difference in absorbance between active substrates (heliamine **8** and 6,7-dimethoxy-1-phenyl-1,2,3,4-tetrahydroisoquinoline **11**), inactive compound **9**, confirmed by HPLC and crystallography) and controls (Boiled enzyme and –AdoMet controls). In this assay AdoHcy resulting from substrate methylation, is transformed by SAAH to homocysteine which reacted with the chromene NDCC leading to a color change that can be detected at 405 nm

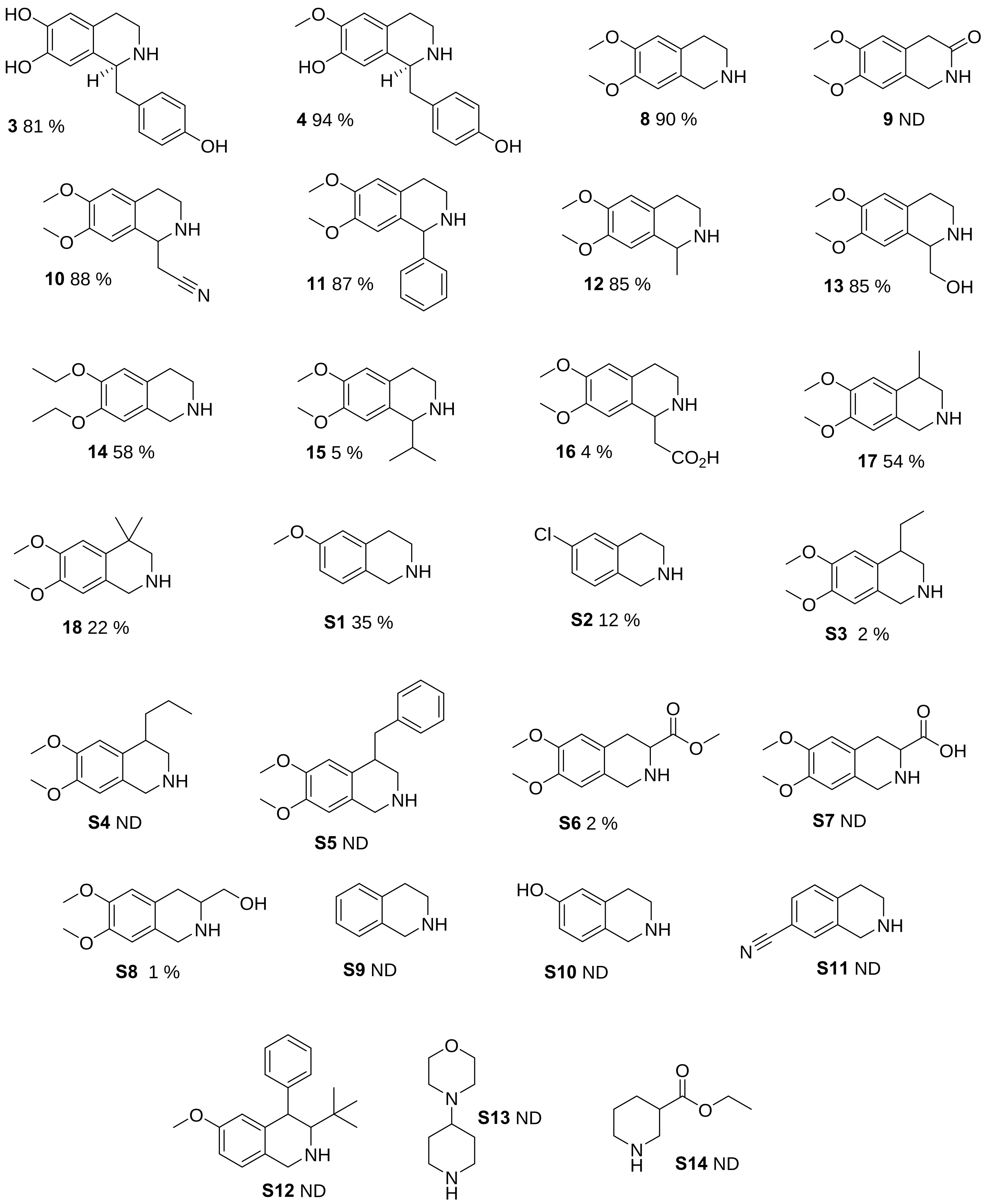

Figure S15 Substrates of CNMT with HPLC conversions after 45 minutes. ND – no products detected.

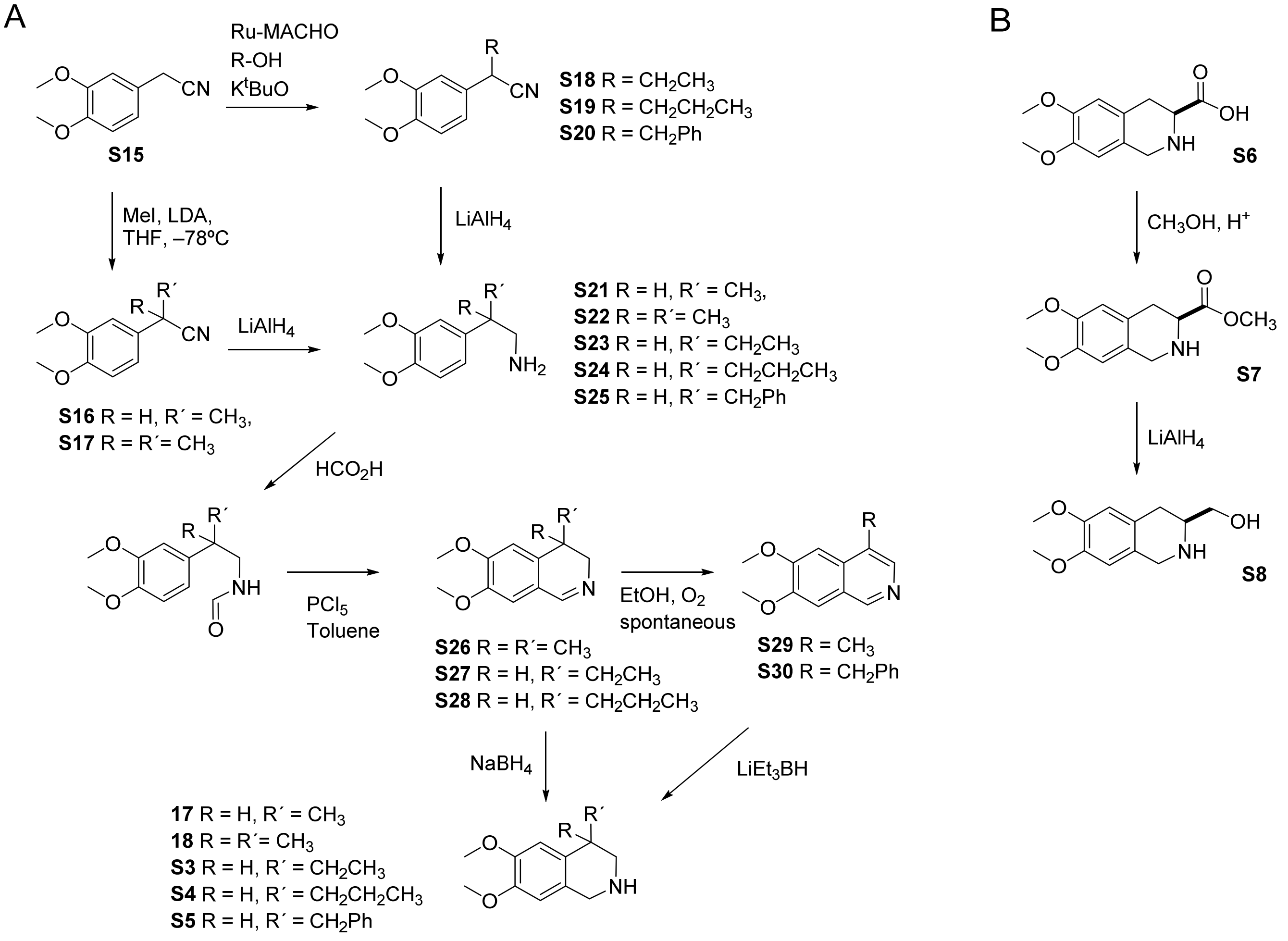

Figure S16 - (A) Synthesis of C4-substituted THIQs. There are a few reports describing synthesis of C4-substituted THIQs.[S11-14] but these do not include the derivatives we required. Consequently, it was necessary to develop/adapt a route to C4-substituted THIQs. Alkylation of nitrile **S15** by standard procedures, with LDA and alkyl halides in THF at –78˚C,[S7] resulted in a mixture of mono- and di-alkylated products (e.g. **S16 & S17**). As an alternative, Ru-catalysed alkylation of nitrile **S15** with alcohols[S8] was more reliable and gave higher yields of alkylated products **S18-20**. Attempts to reduce the α-alkyl nitriles **S16-S20** to amines with $CoCl_2$ and $NaBH_4$[S15] or using borane dimethylsulphide was unproductive. However, reduction with $LiAlH_4$ gave the required amines **S21-S25** in higher yields. Cyclisation of the crude amines **S21-S25** to the THIQs **17, 18, S3-S5** using paraformaldehyde and acid catalysis (Pictet–Spengler reaction),[S11-12, S16] failed, or gave very low yields of THIQs. In light of this, amines **S21-S25**, were *N*-formylated and then cyclised to the corresponding imines using PCl_5 (Bischler-Napieralski reaction).[S17] This resulted in imines **S26-S28,** which could then be reduced to the THIQs **17, 18 & S3** using $NaBH_4$. However formylation and cyclisation of amines **S21** and **S25** resulted in isoquinolines (**S29 & S30**), which required reduction with $LiEt_3BH$ to afford THIQs **18** and **S5.**[S6] **(B)** Synthesis of C3-substituted THIQs was achieved by methylation and reduction of the commercially available C3-carboxylic acid.[S9, 10]

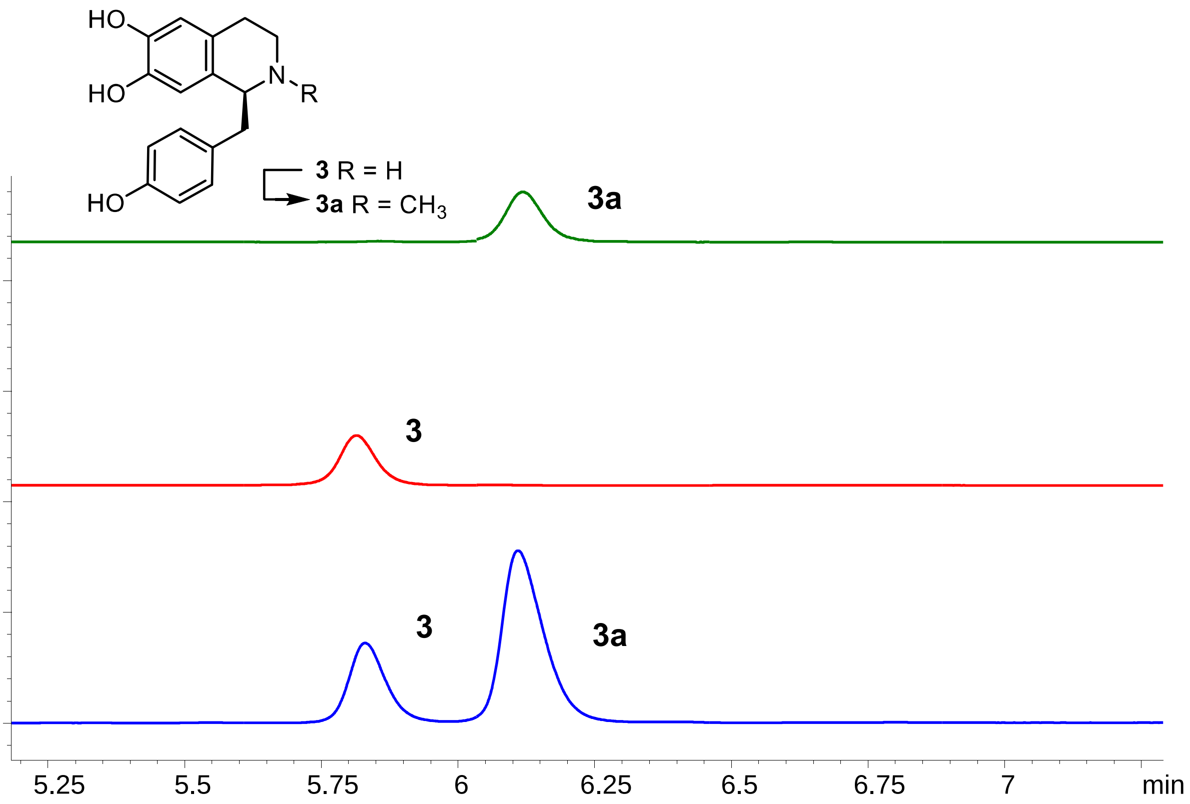

Figure S17. HPLC analysis from the *N*-methylation reaction of norcoclaurine **3** (0.5 mM) to give *N*-methyl norcoclaurine **3a** with CNMT (5 µM), AdoMet (3 mM) after 30 minutes incubation at 30°C (blue). HPLC analysis of **3** (red) and **3a** (green) standards are shown above.

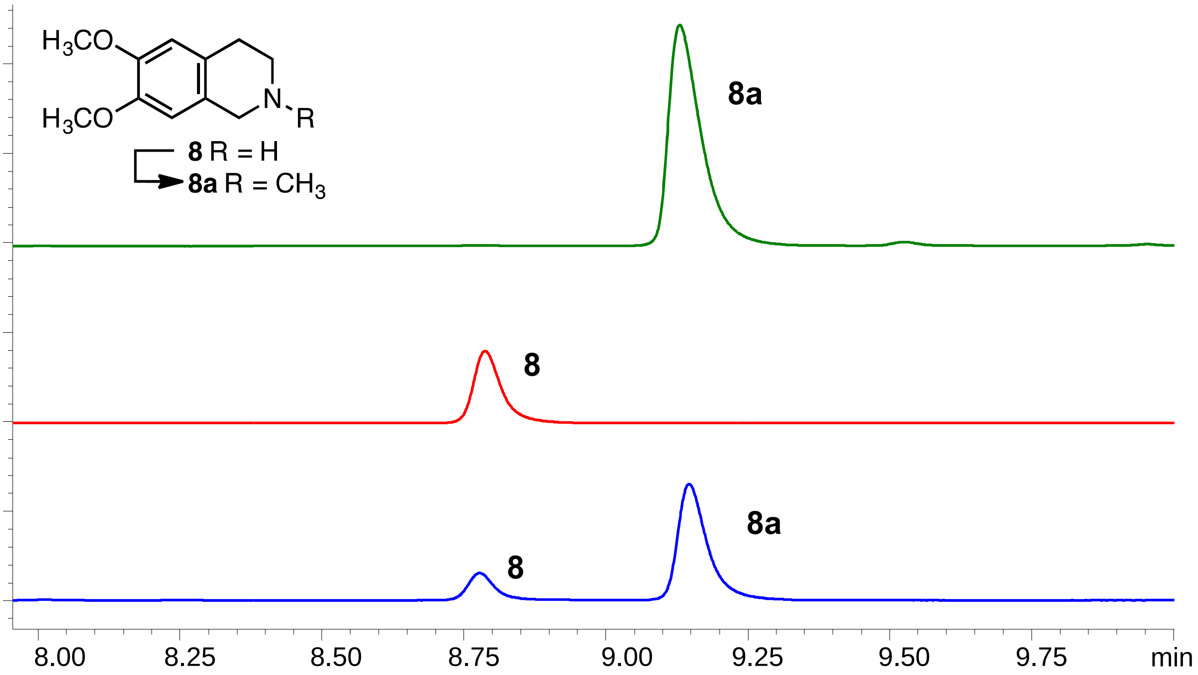

Figure S18. HPLC analysis from the *N*-methylation reaction of heliamine **8** (0.5 mM) to give *N*-methylheliamine **8a** with CNMT (5 µM), AdoMet (3 mM) after 30 minutes incubation at 30°C (blue). HPLC analysis of heliamine **8** (red) and *N*-methylheliamine **8a** (green) standards are shown above.

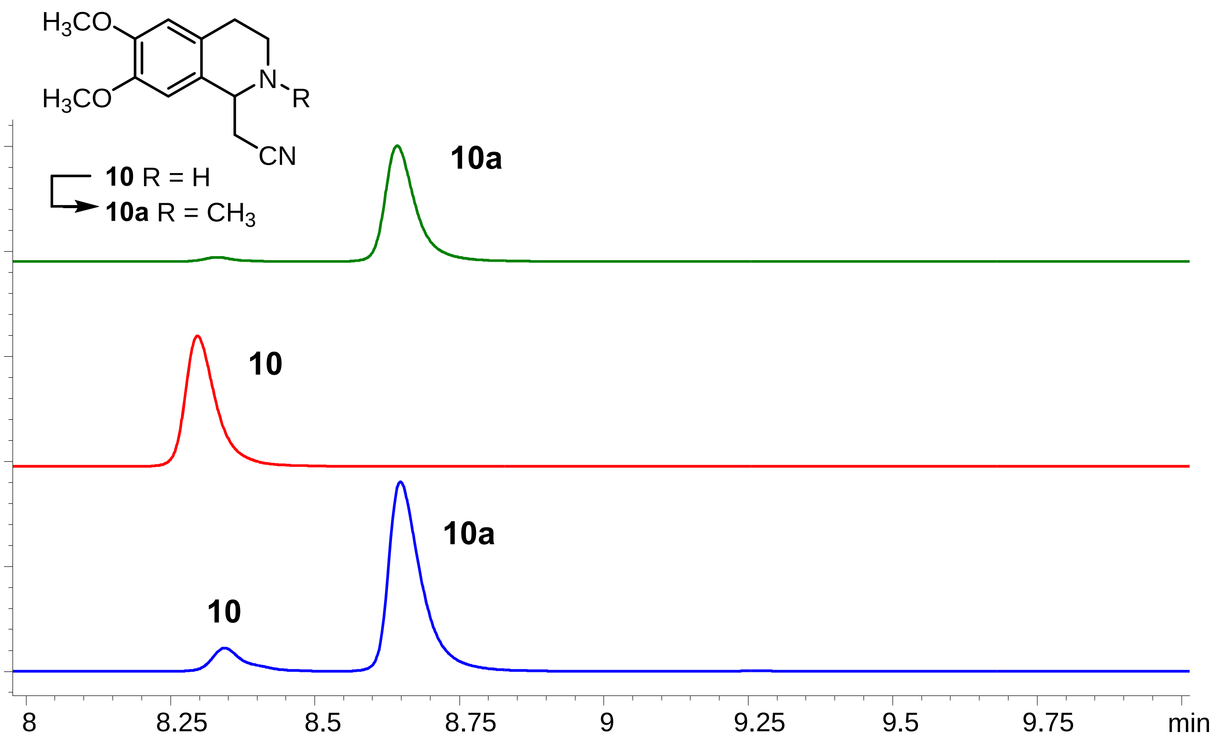

Figure S19. HPLC analysis from the *N*-methylation reaction of 2-(6,7-dimethoxy-1,2,3,4-tetrahydroisoquinolin-1-yl)acetonitrile **10** (0.5 mM) to give *N*-methyl-2-(6,7-dimethoxy-1,2,3,4-tetrahydroisoquinolin-1-yl)acetonitrile **10a** with CNMT (5 µM), AdoMet (3 mM) after 30 minutes incubation at 30°C (blue). HPLC analysis of **10** (red) and **10a** (green) standards are shown above.

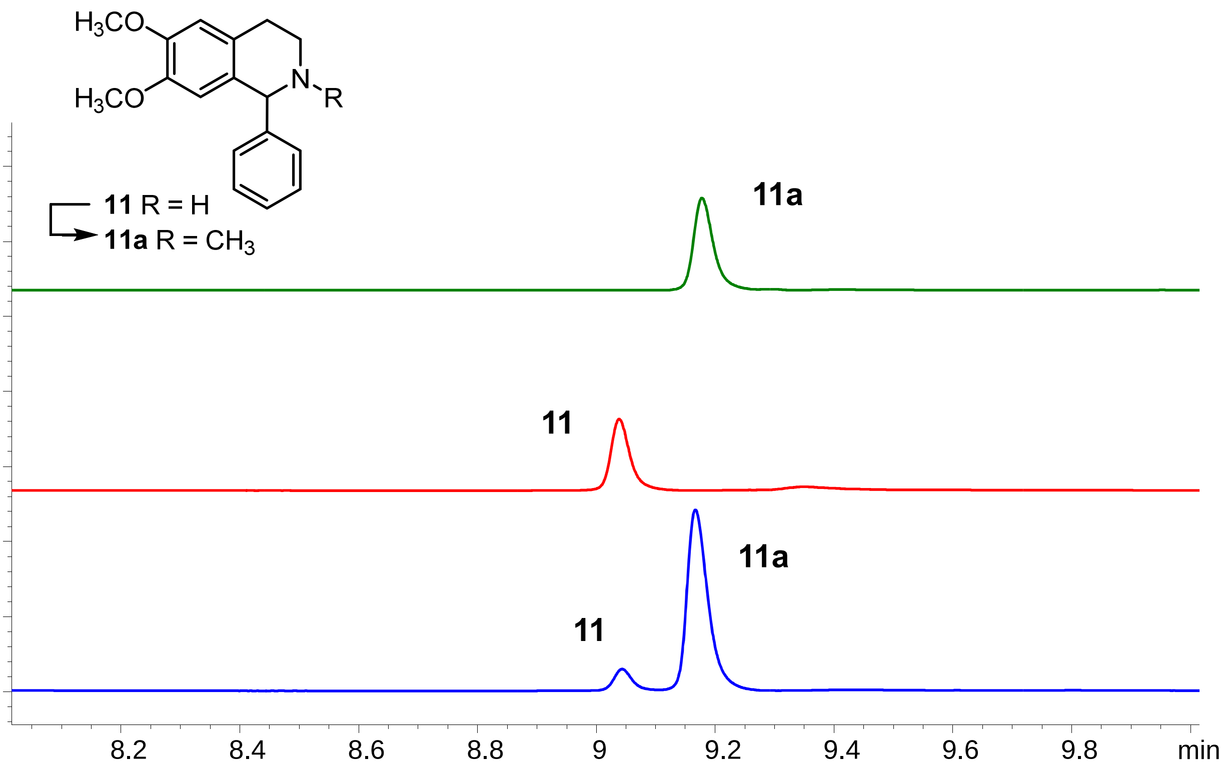

Figure S20. HPLC analysis from the *N*-methylation reaction of 6,7-dimethoxy-1-phenyl-1,2,3,4-tetrahydroisoquinoline **11** (0.5 mM) to give *N*-methyl-6,7-dimethoxy-1-phenyl-1,2,3,4-tetrahydroisoquinoline **11a** with CNMT (5 µM), AdoMet (3 mM) after 30 minutes incubation at 30°C (blue). HPLC analysis of **11** (red) and 11**a** (green) standards are shown above.

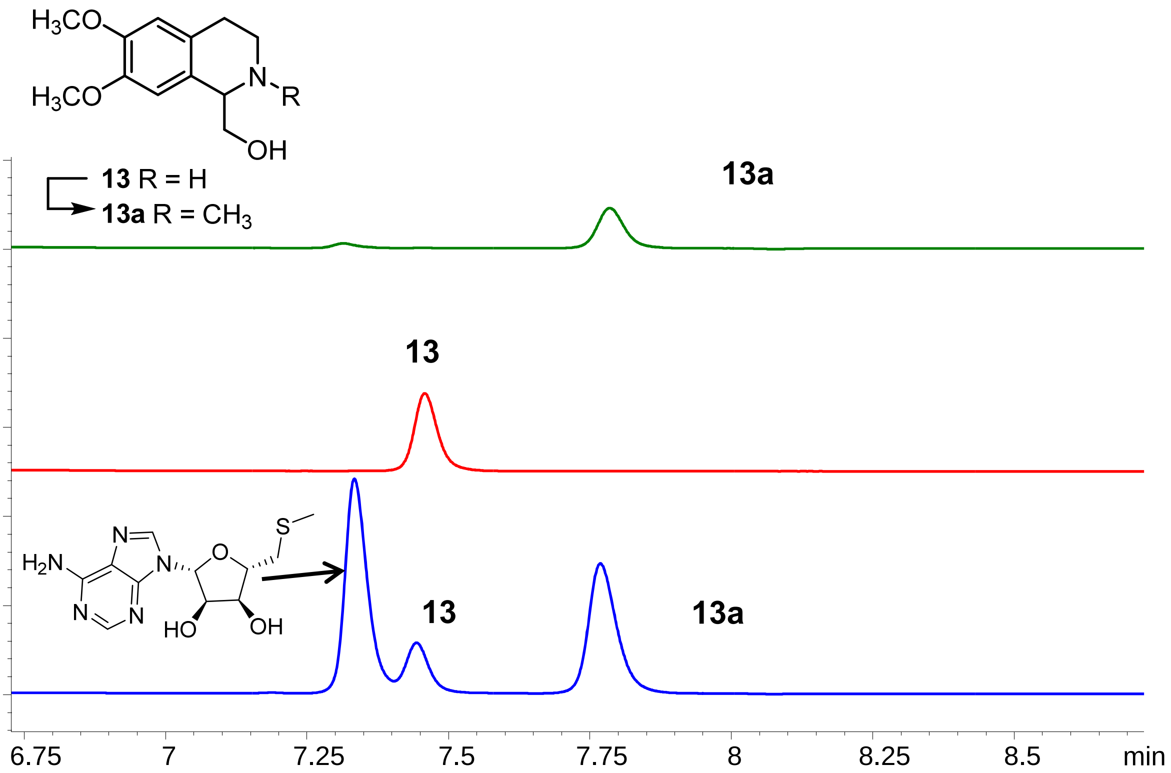

Figure S21. HPLC analysis from the *N*-methylation reaction of 6,7-dimethoxy-1,2,3,4-tetrahydroisoquinolin-1-yl)methanol **13** (0.5 mM) to give *N*-methyl-6,7-dimethoxy-1,2,3,4-tetrahydroisoquinolin-1-yl)methanol **13a** with CNMT (5 µM), AdoMet (3 mM) after 30 minutes incubation at 30°C (blue). HPLC analysis of **13** (red) and **13a** (green) standards are shown above. *S*-methyl-5'-thioadenosine is a common by-product from assay with AdoMet

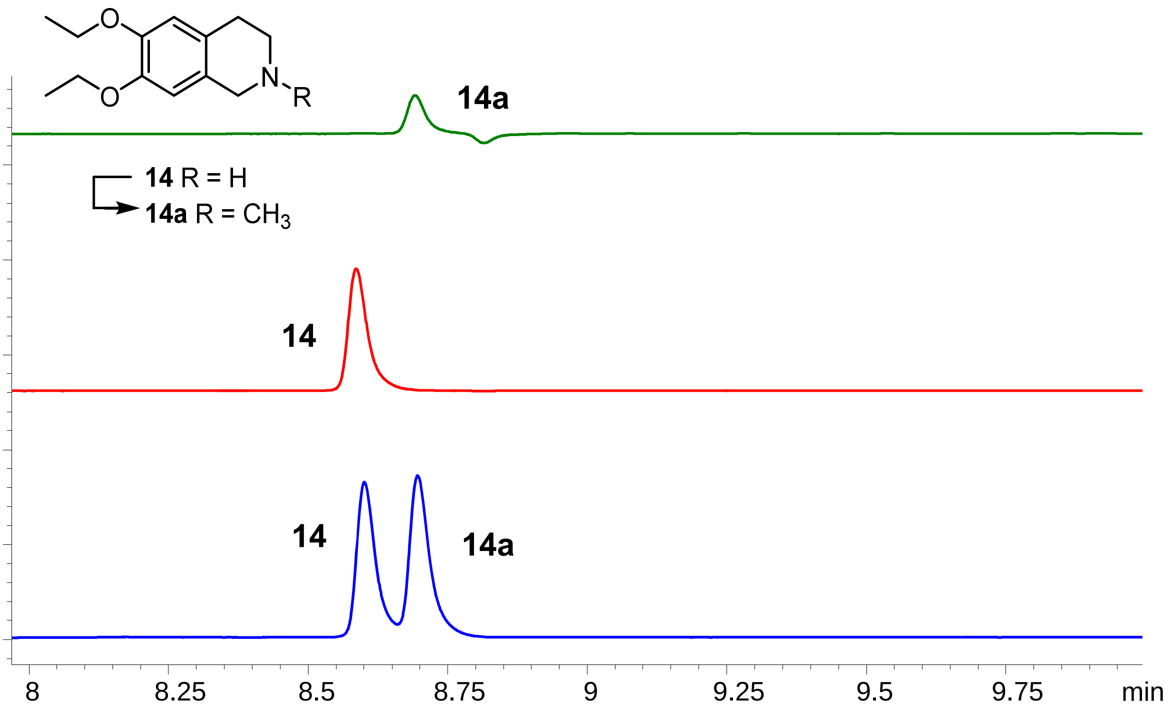

Figure S22. HPLC analysis from the *N*-methylation reaction of 6,7-diethoxy-1,2,3,4-tetrahydroisoquinoline **14** (0.5 mM) to give *N*-methyl-6,7-diethoxy-1,2,3,4-tetrahydroisoquinoline **14a** with CNMT (5 µM), AdoMet (3 mM) after 30 minutes incubation at 30°C (blue). HPLC analysis of **14** (red) and **14a** (green) standards are shown above.

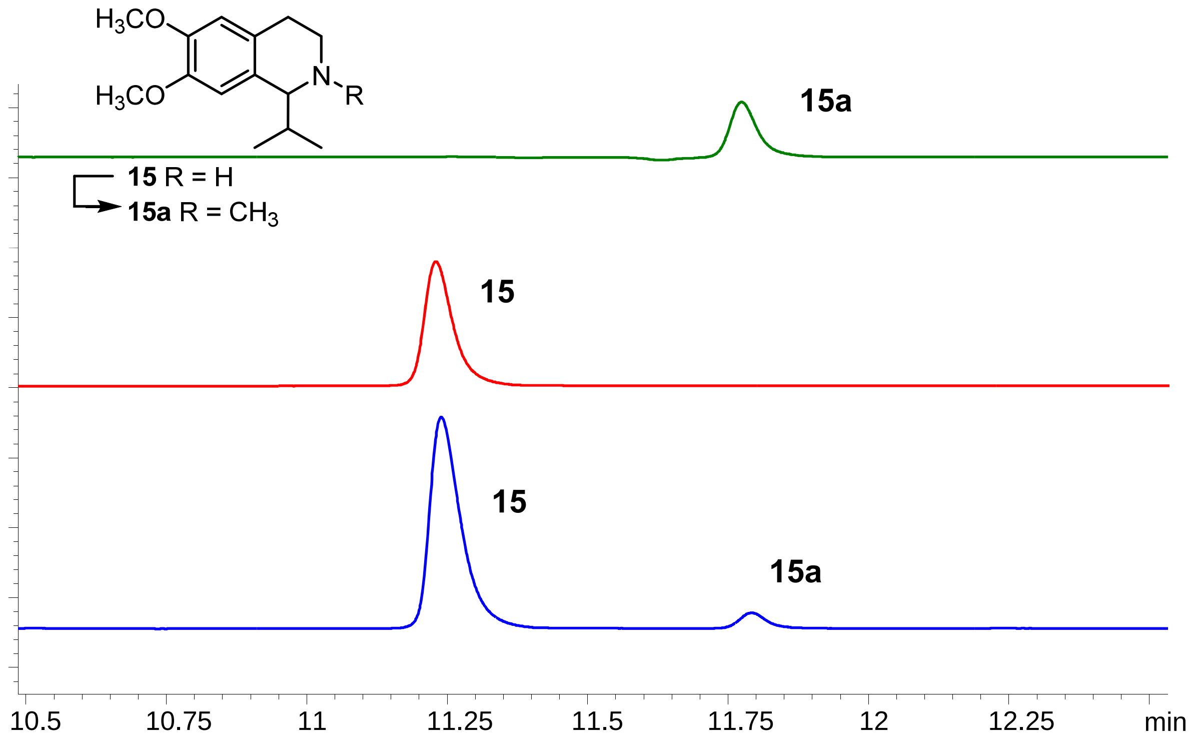

Figure S23. HPLC analysis from the *N*-methylation reaction of 1-isopropyl-6,7-dimethoxy-1,2,3,4-tetrahydroisoquinoline **15** (0.5 mM) to give *N*-methyl-1-isopropyl-6,7-dimethoxy-1,2,3,4-tetrahydroisoquinoline **15a** with CNMT (5 μM), AdoMet (3 mM) after 30 minutes incubation at 30°C (blue). HPLC analysis of **15** (red) and **15a** (green) standards are shown above.

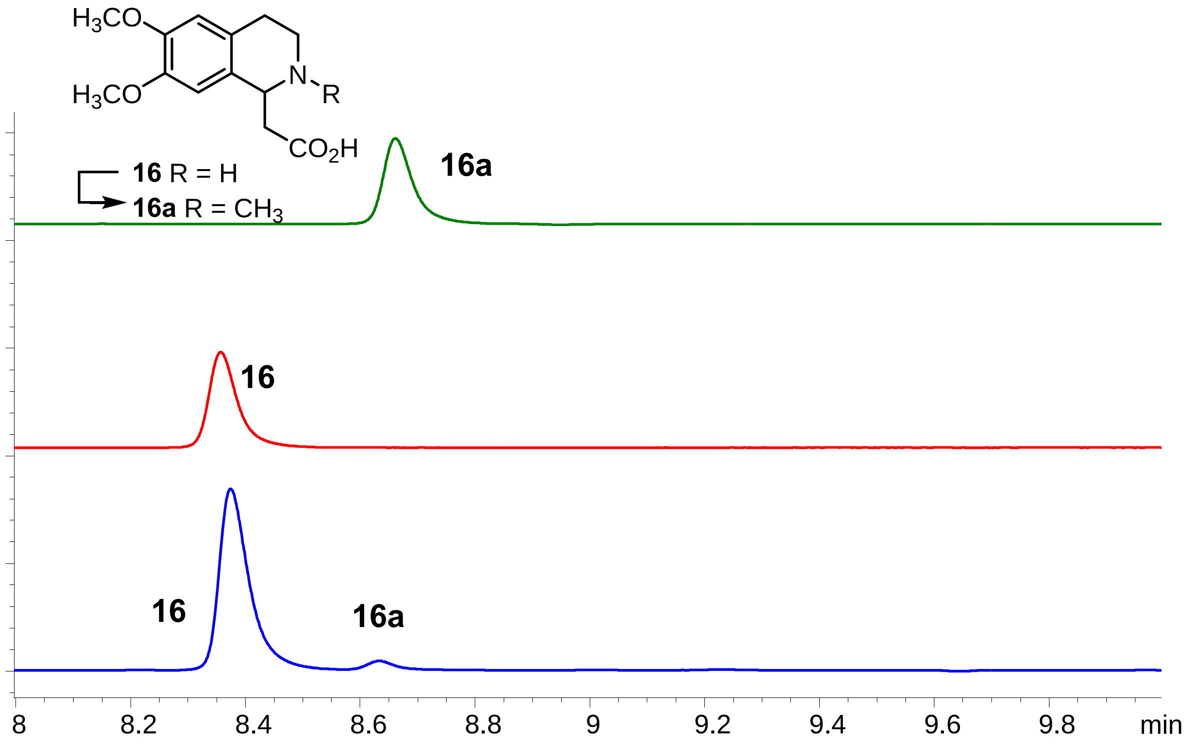

Figure S24. HPLC analysis from the *N*-methylation reaction of 2-(6,7-dimethoxy-1,2,3,4-tetrahydroisoquinolin-1-yl)acetic acid **16** (0.5 mM) to give 2-(6,7-dimethoxy-1,2,3,4-tetrahydroisoquinolin-1-yl)acetic acid **16a** with CNMT (5 μM), AdoMet (3 mM) after 30 minutes incubation at 30°C (blue). HPLC analysis of **16** (red) and **16a** (green) standards are shown above.

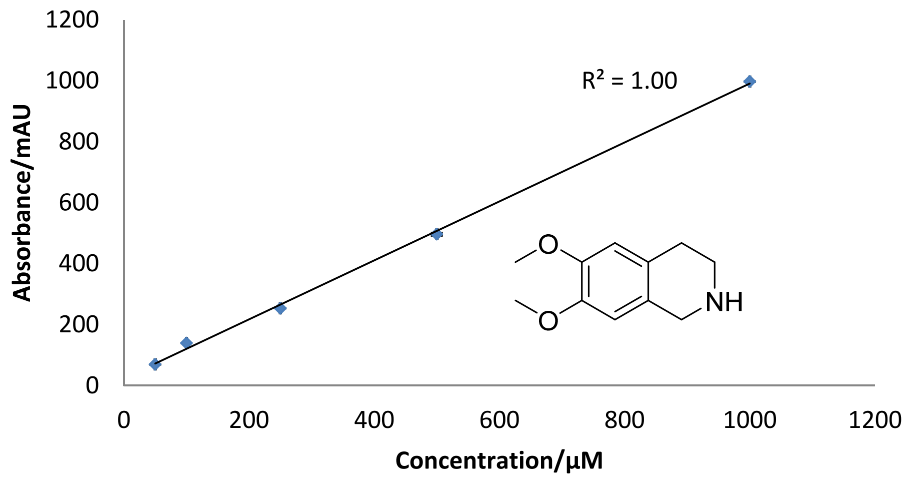

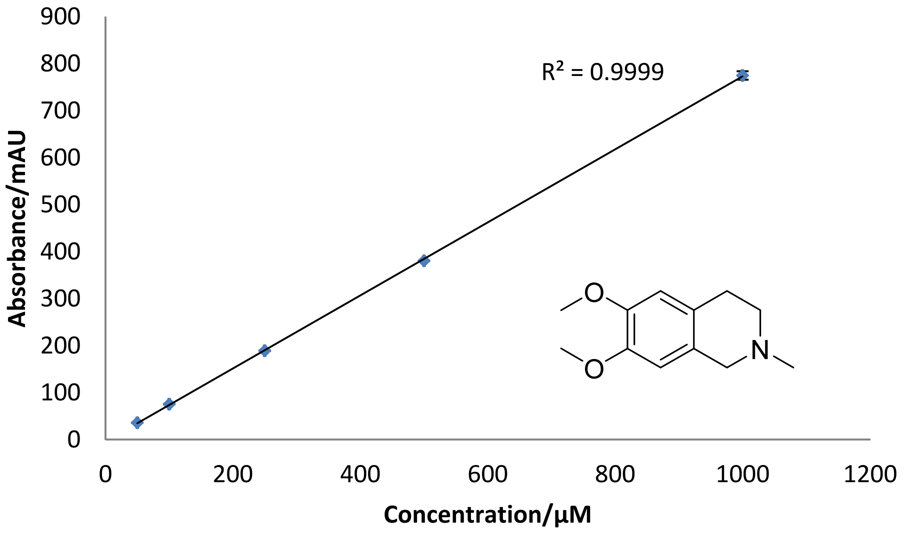

Figure S25. Example of HPLC calibrations (wavelength 283 nm) for tetrahydroisoquinoline substrates and products

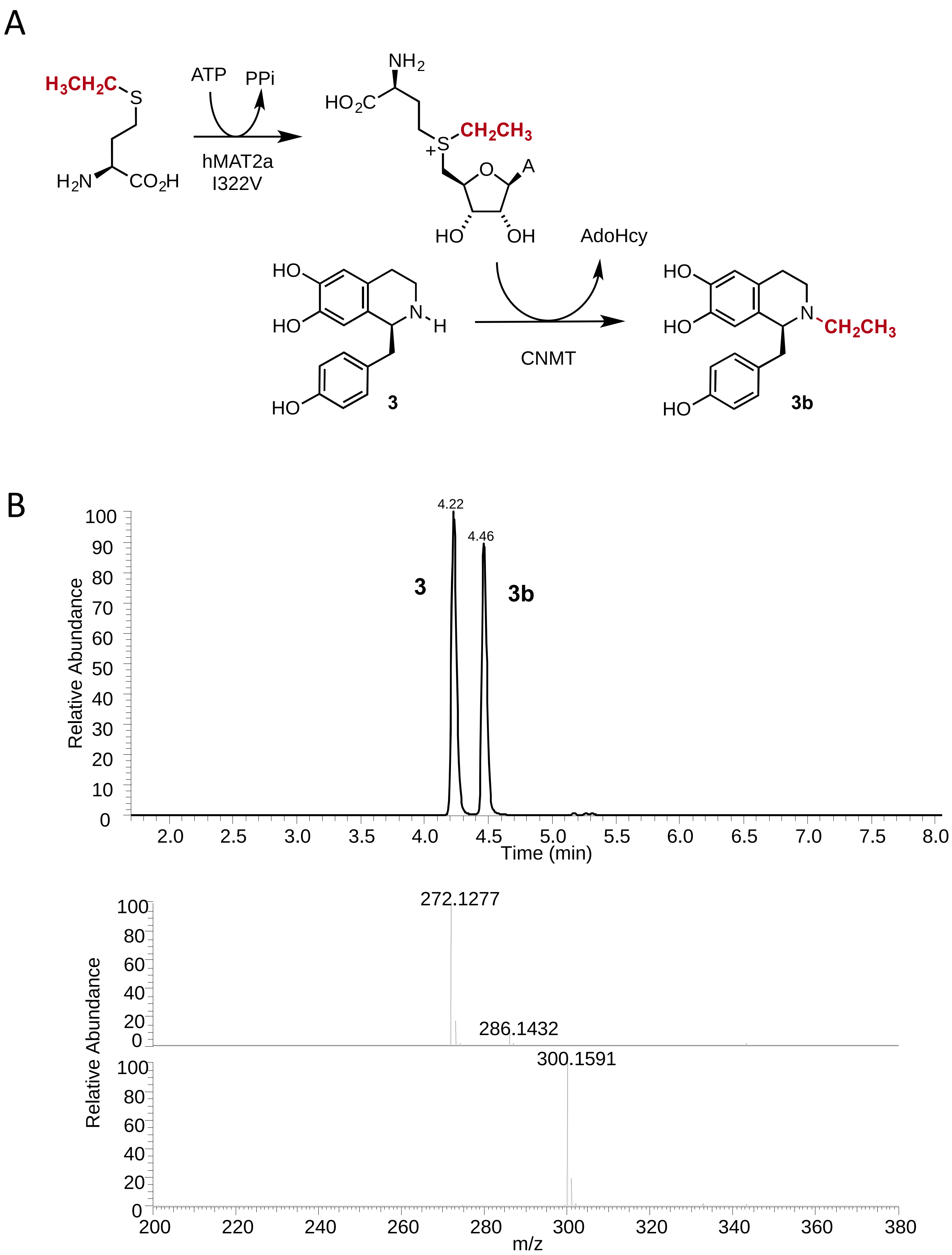

Figure S26 (**A**) CNMT catalysed ethylation of norcoclaurine **3** using ethyl-AdoMet generated *in situ* from L-ethionine and ATP using hMAT. (**B**) High resolution LCMS of norcoclaurine ethylation, norcoclaurine **3** (t_r = 4.22; m/z $[M+H]^+$ calc'd 272.1281 , observed 272.1277) and *N*-ethyl-norcoclaurine **3b** (t_r = 4.46; m/z $[M+H]^+$ calc'd 300.1594, observed 300.1591).

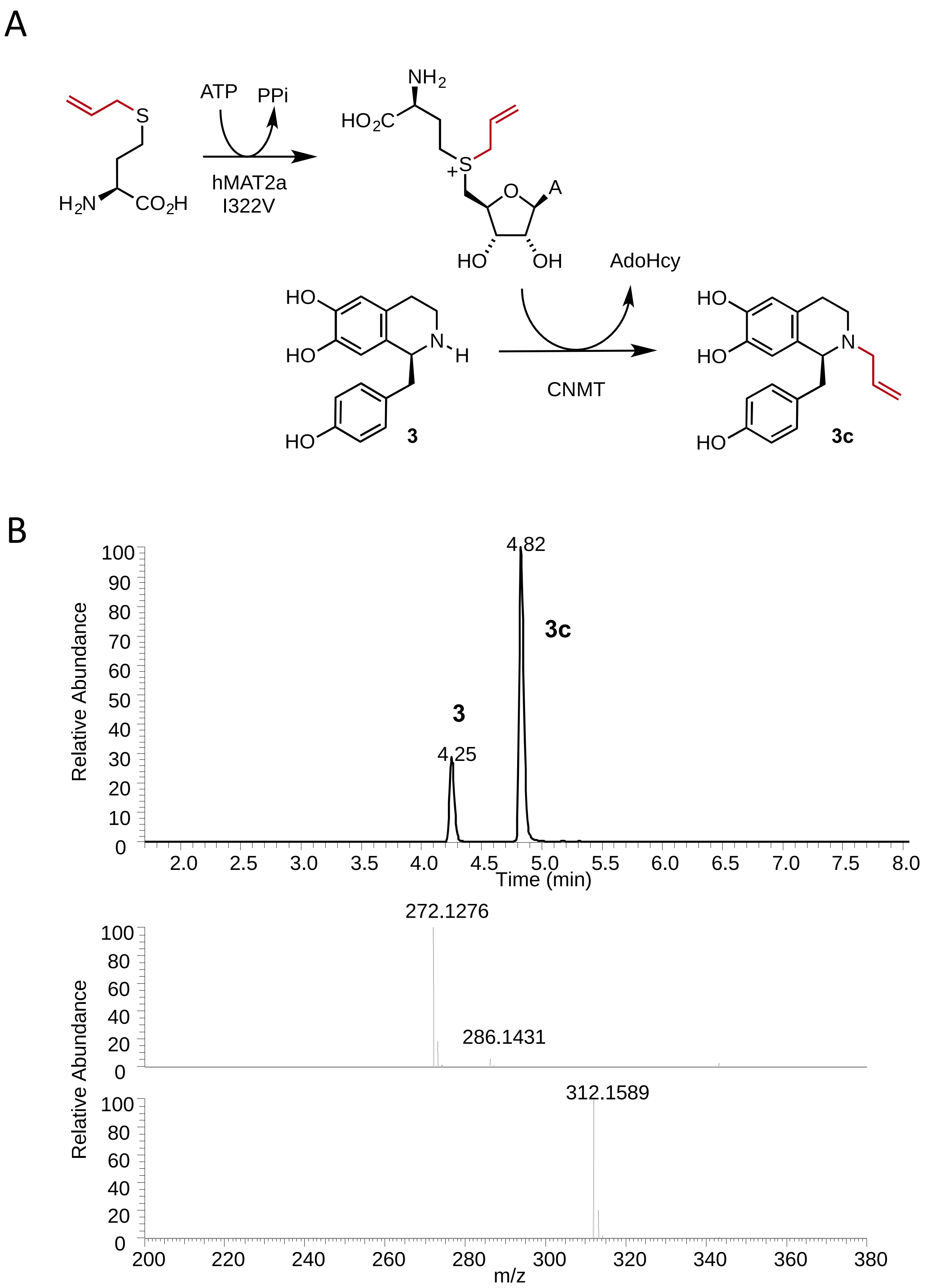

Figure S27 (**A**) CNMT catalysed allylation of norcoclaurine **3** using allyl-AdoMet generated *in situ* from S-allyl-Lhomocysteine and ATP using hMAT. (**B**) High resolution LCMS of norcoclaurine allylation, norcoclaurine **3** (t_r = 4.25; m/z $[M+H]^+$ calc'd 272.1281 , observed 272.127) and *N*-allyl-norcoclaurine **3c** (t_r = 4.82; m/z $[M+H]^+$ calc'd 312.1594, observed 312.1589).

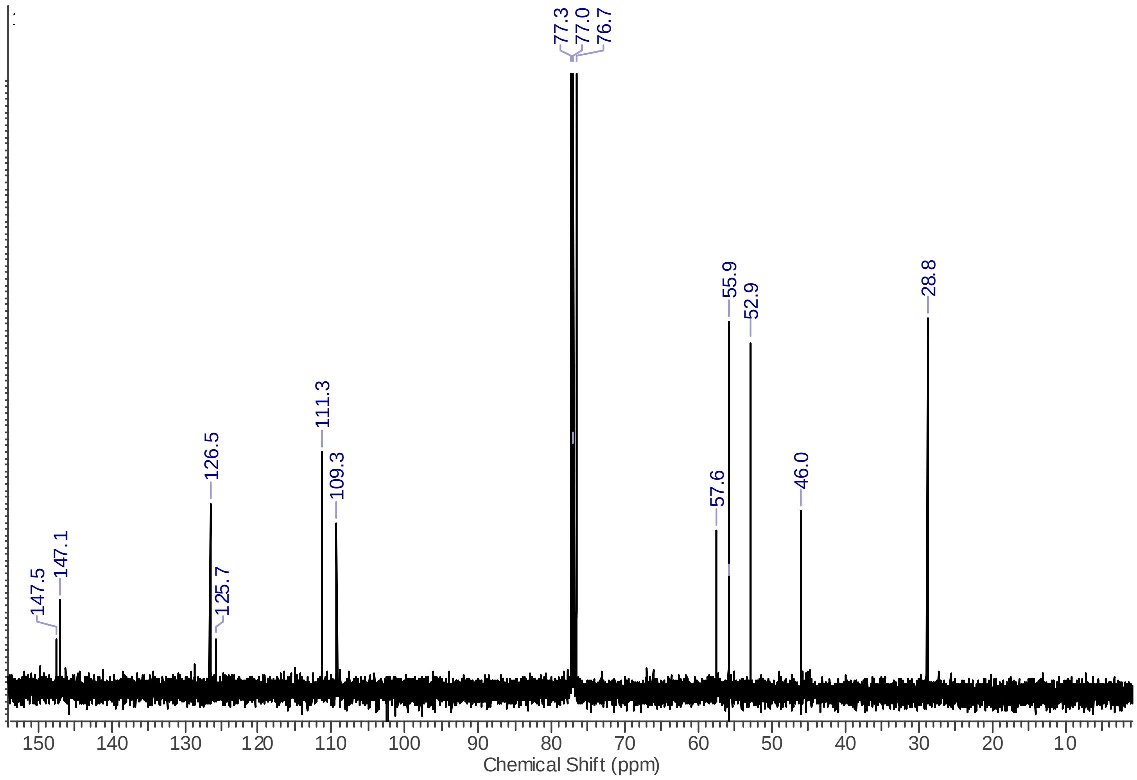

Figure S28:^{13}C of *N*-methylheliamine (**8a**) from CNMT CLEA.

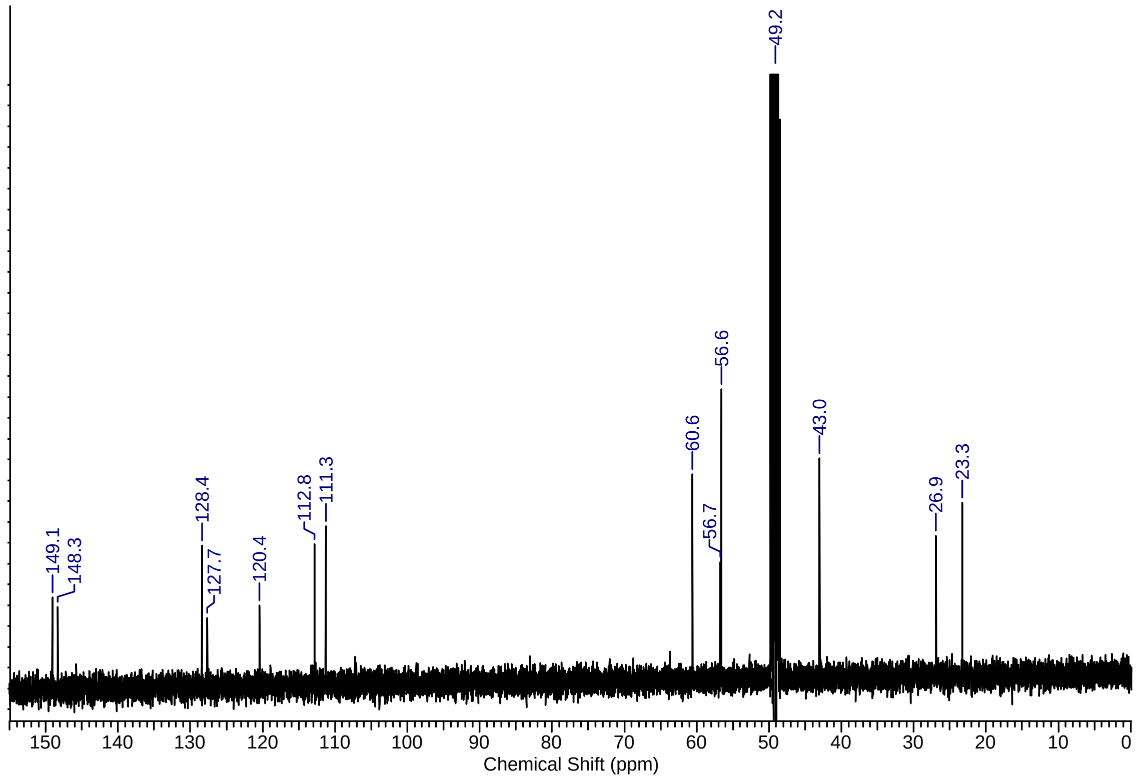

Figure S29: ^{13}C of *N*-methyl-2-(6,7-dimethoxy-1,2,3,4-tetrahydroisoquinolin-1-yl)acetonitrile (**10a**). From CNMT CLEA

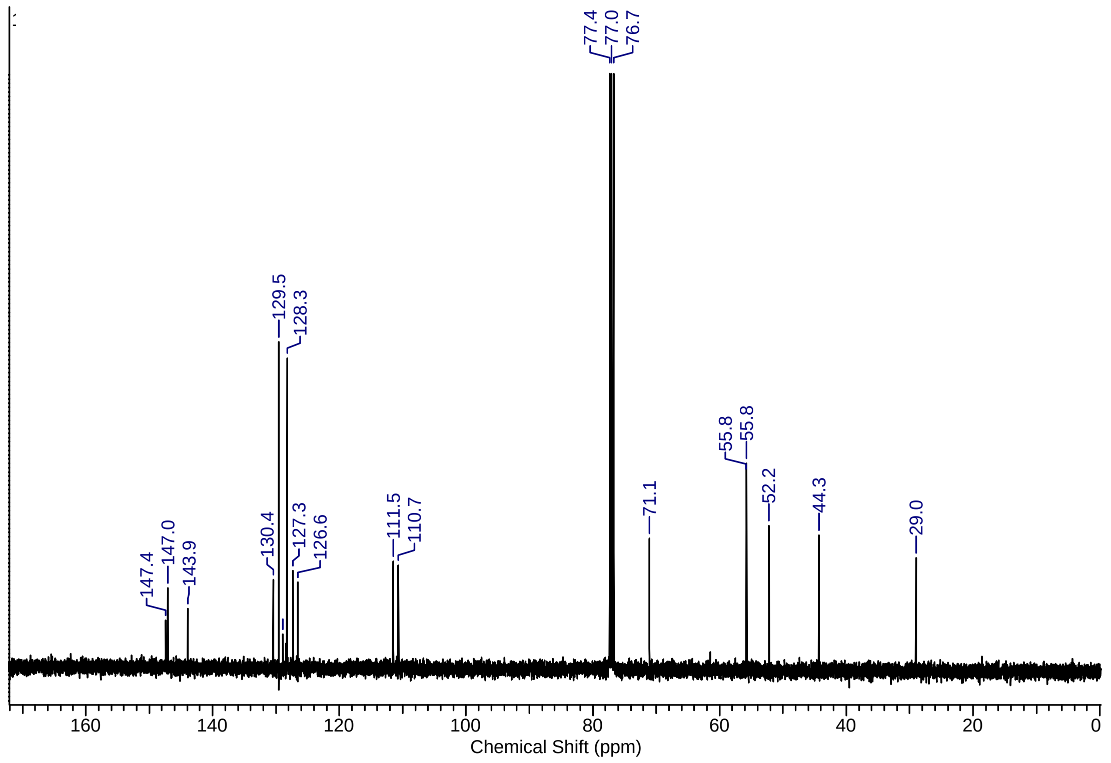

Figure S30: ^{13}C of *N*-methyl-6,7-dimethoxy-1-phenyl-1,2,3,4-tetrahydroisoquinoline (**11a**) from CNMT CLEA.

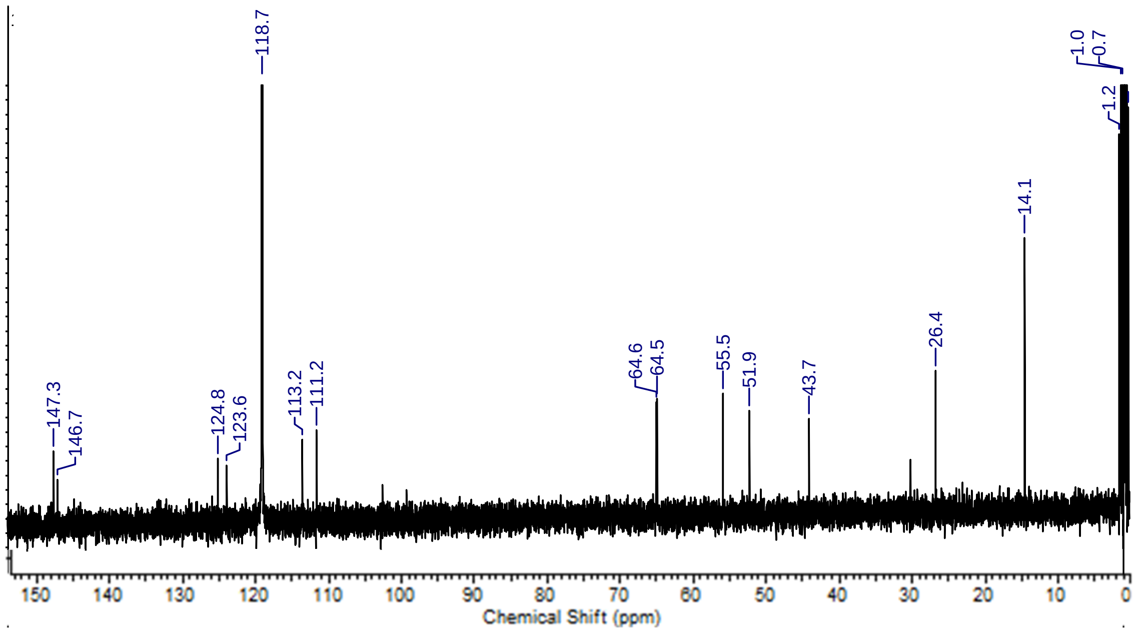

Figure S31: ^{13}C of *N*-methyl-1-6,7-diethoxy-1,2,3,4-tetrahydroisoquinoline **(14a)** from CNMT CLEA.

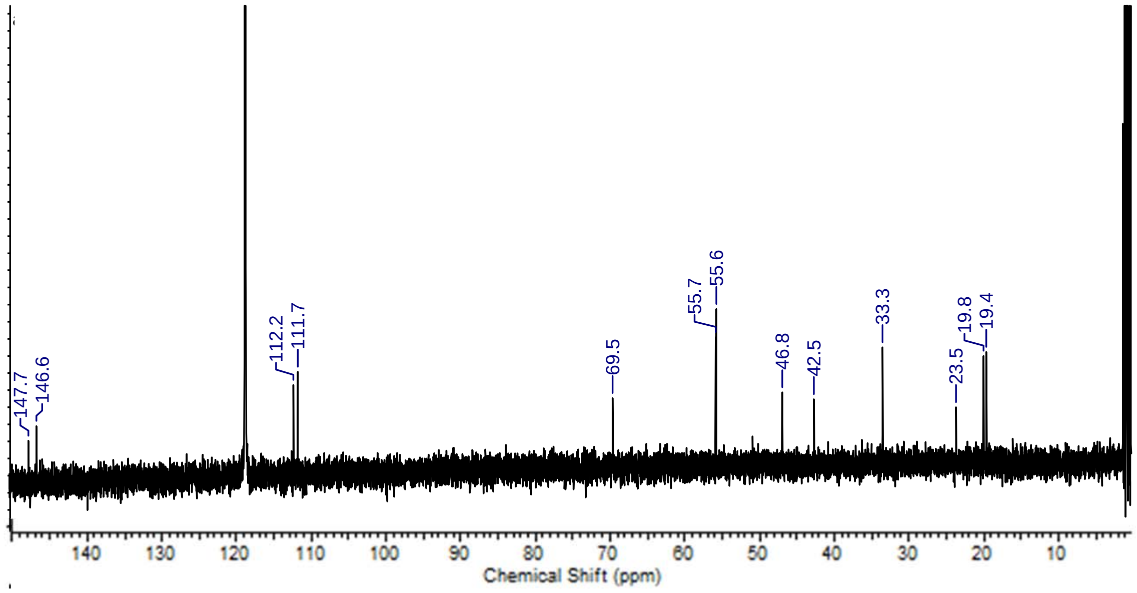

Figure S32: ^{13}C of *N*-methyl-1-isopropyl-6,7-dimethoxy-1,2,3,4-tetrahydroisoquinoline (**15a**) from CNMT CLEA.

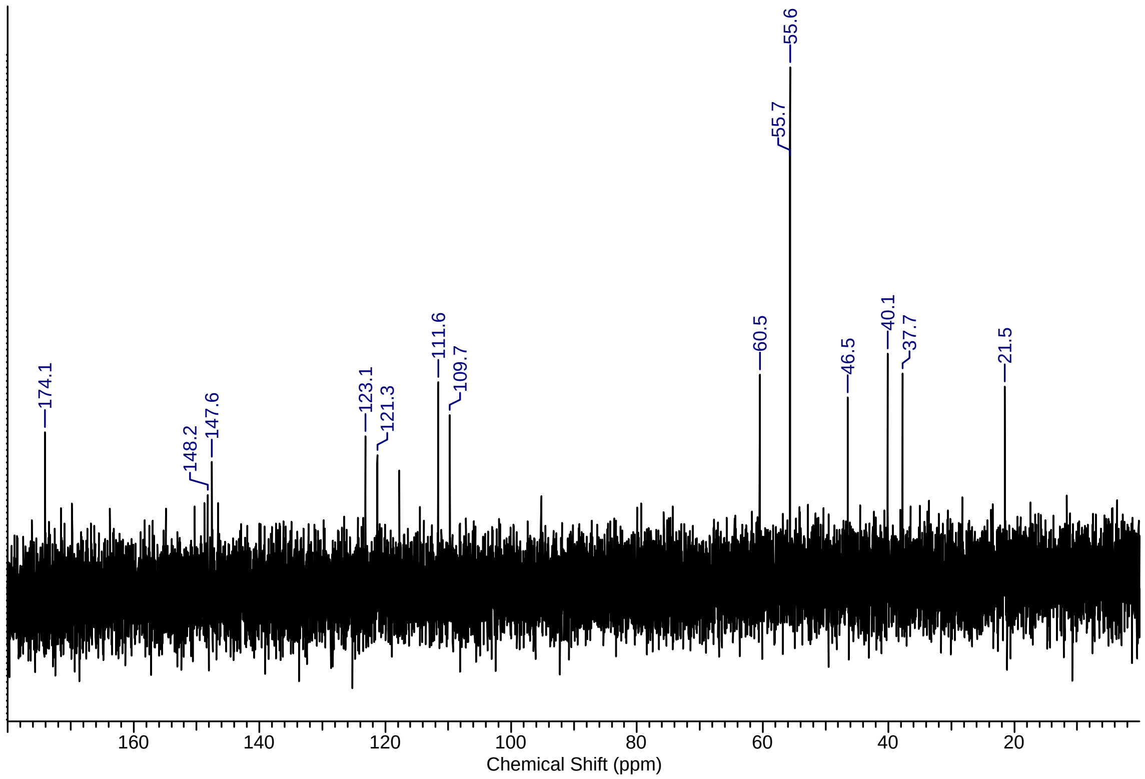

Figure S33: ^{13}C of *N*-methyl-2-(6,7-dimethoxy-1,2,3,4-tetrahydroisoquinolin-1-yl)acetic acid (**16a**) from CNMT CLEA.

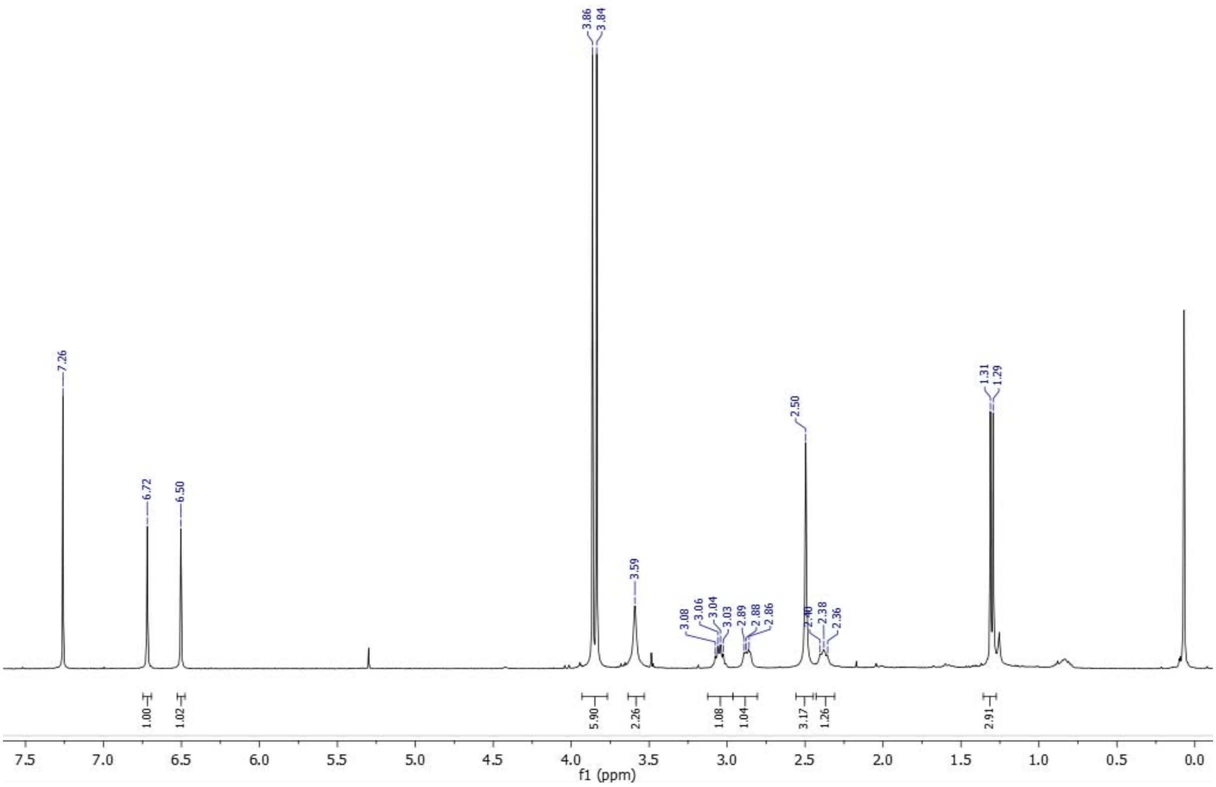

Figure S34: ^{1}H of (**17a**) from CNMT CLEA.

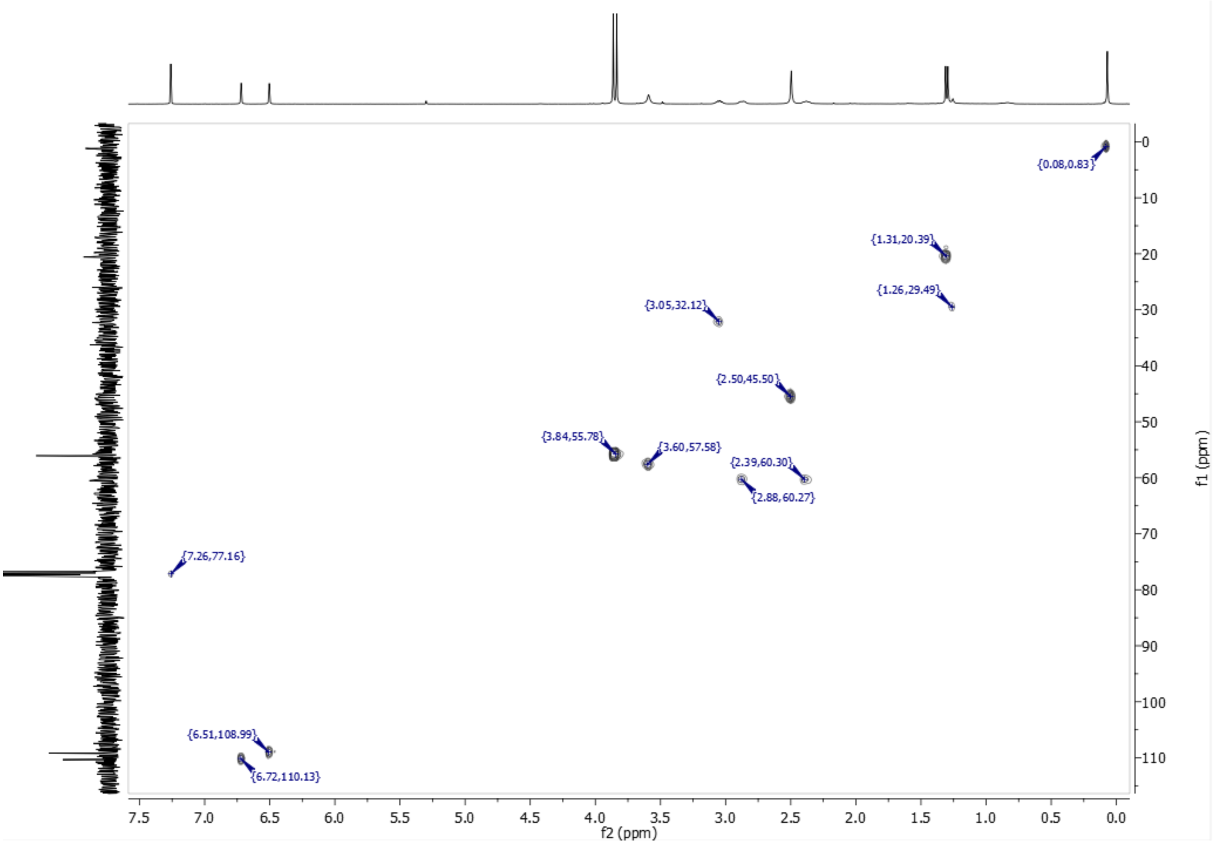

Figure S35: HSQC of (**17a**) from CNMT CLEA.

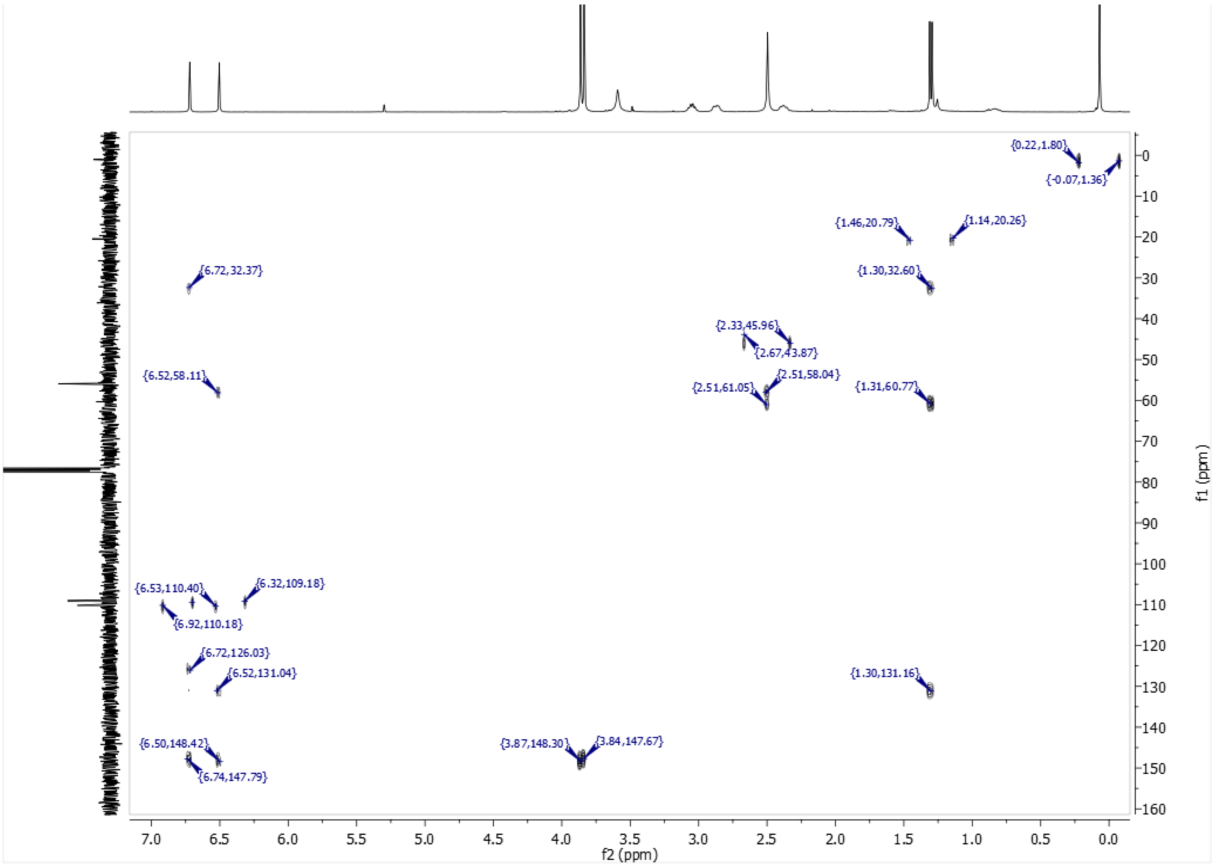

Figure S36: HMBC of (**17a**) from CNMT CLEA.

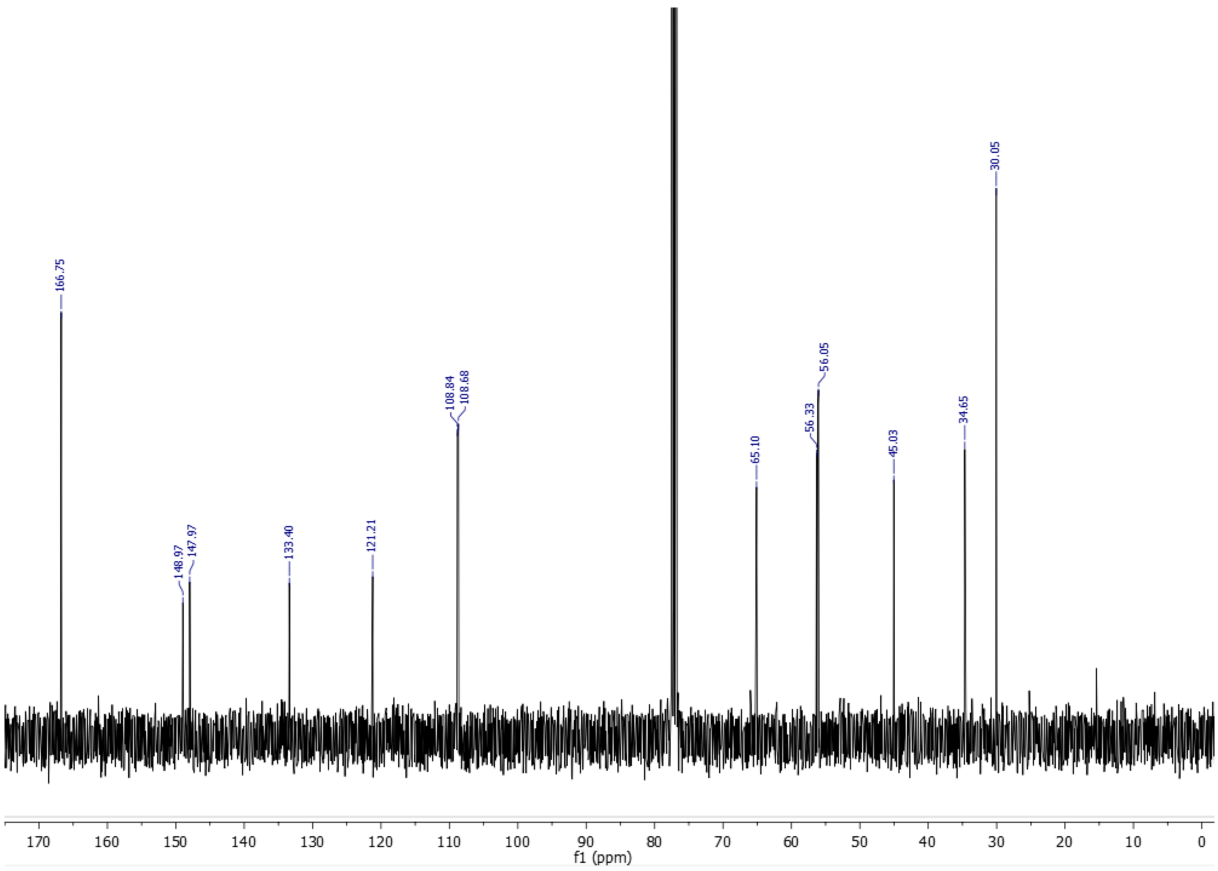

Figure S37: ^{13}C of *N*-methyl-6,7-dimethoxy-4,4-dimethyl-1,2,3,4-tetrahydroisoquinoline (**18a**) from CNMT CLEA.

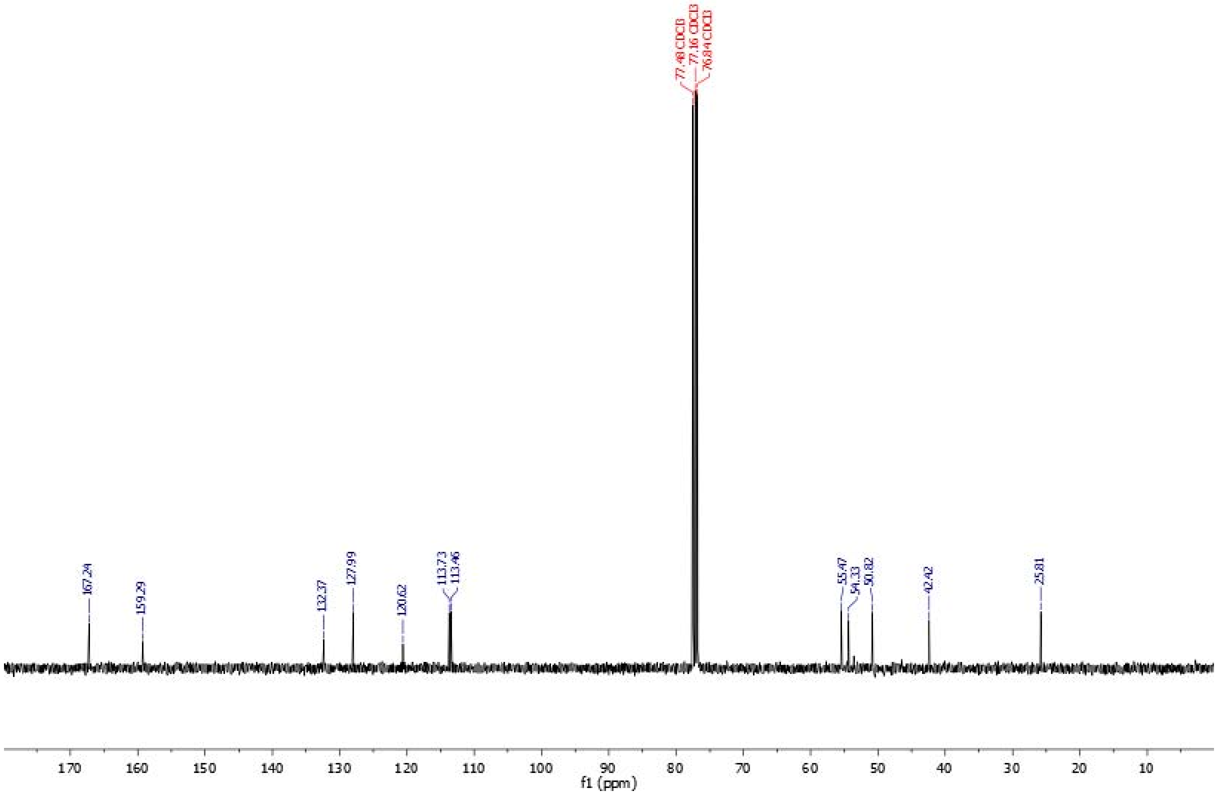

Figure S38: ^{13}C of 6-methoxy-*N*-methyl-1,2,3,4-tetrahydroisoquinoline **(S1a)** from CNMT CLEA.

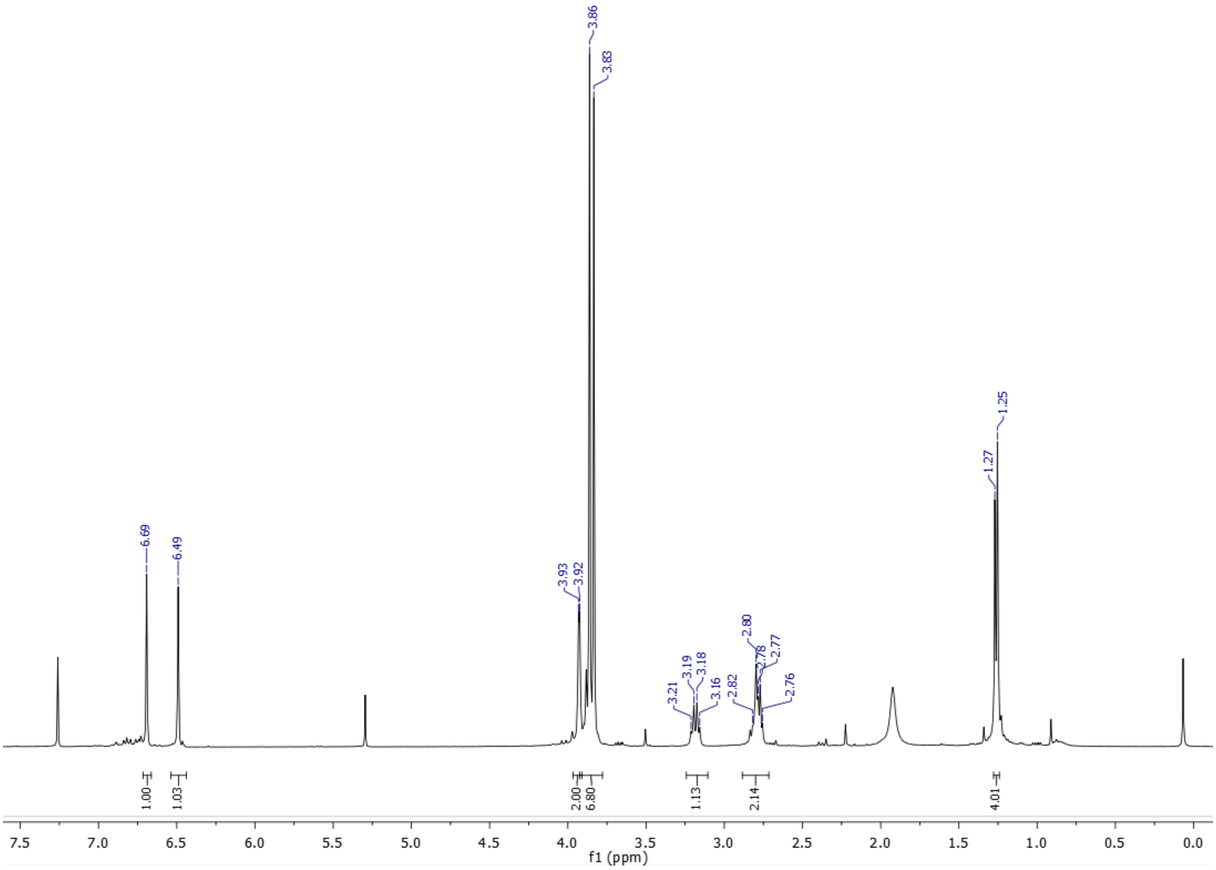

Figure S39: ^{1}H of 6,7-dimethoxy-4-methyl-1,2,3,4-tetrahydroisoquinoline (**17**)

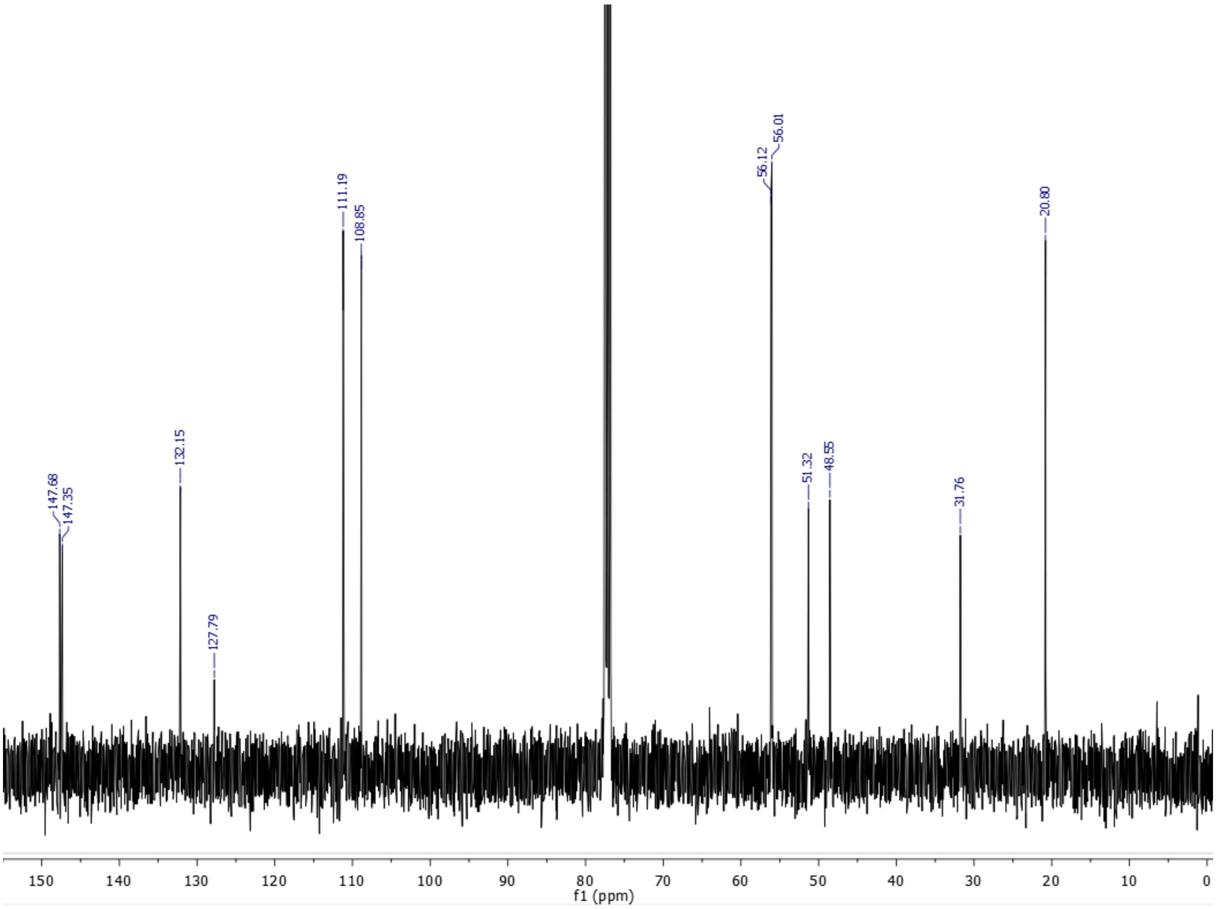

Figure S40: ^{13}C of 6,7-dimethoxy-4-methyl-1,2,3,4-tetrahydroisoquinoline (**17**)

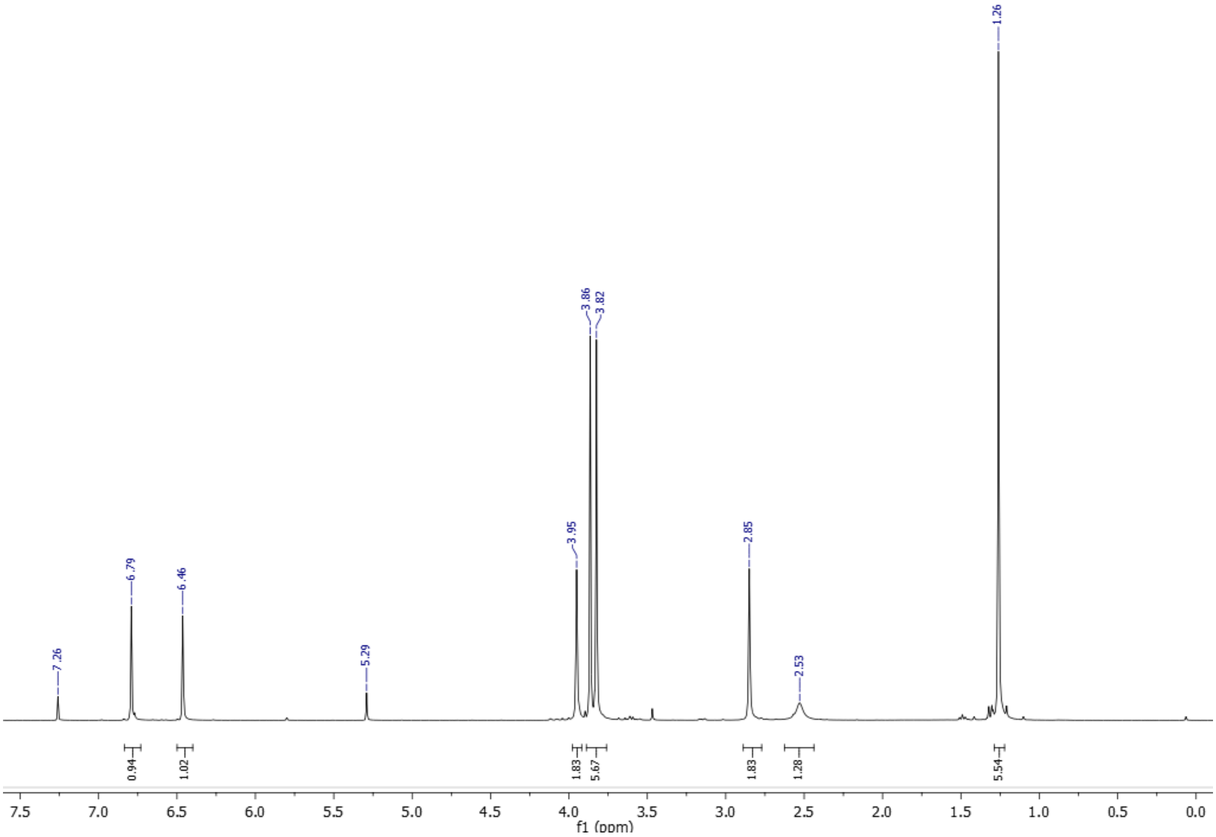

Figure S41: ^{1}H of 6,7-dimethoxy-4,4-dimethyl-1,2,3,4-tetrahydroisoquinonline (**18**)

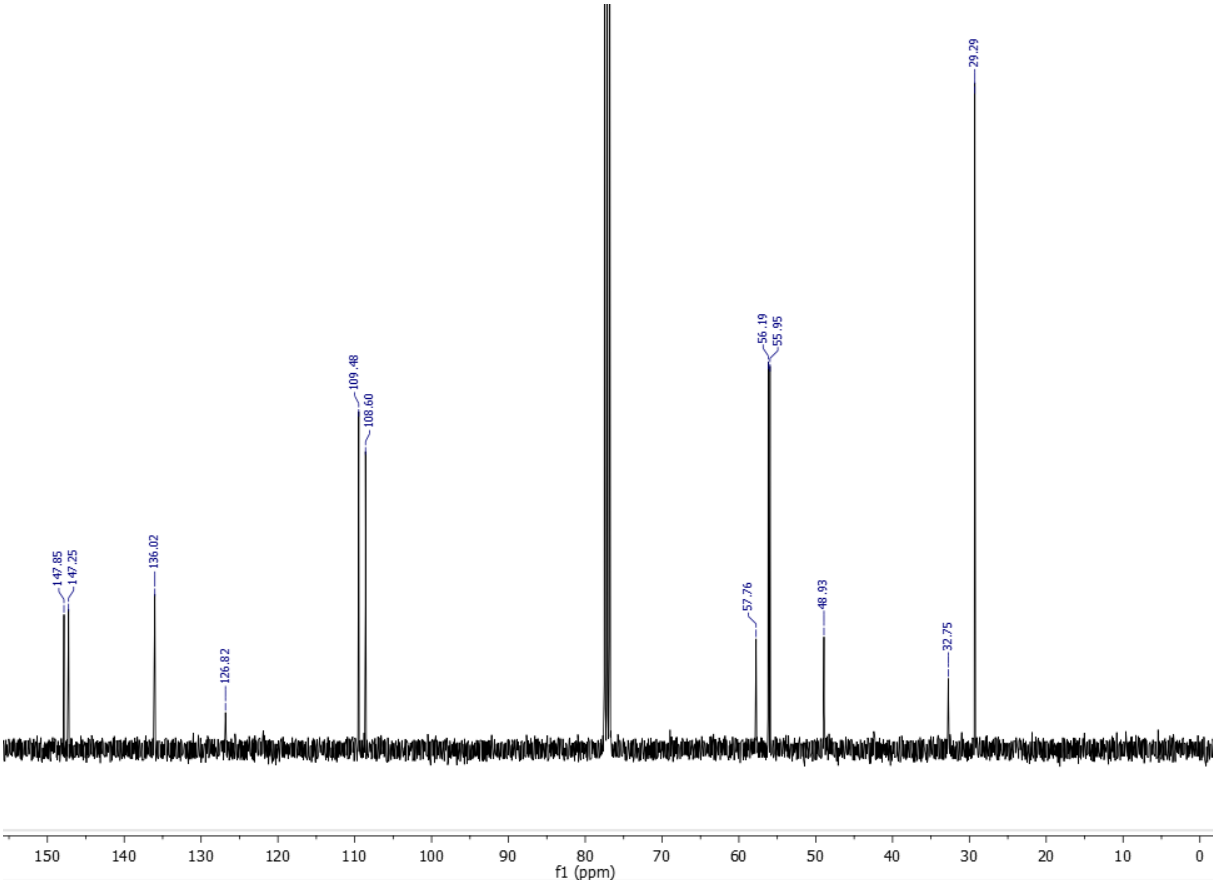

Figure S42: ^{13}C of 6,7-dimethoxy-4,4-dimethyl-1,2,3,4-tetrahydroisoquinonline (**18**)

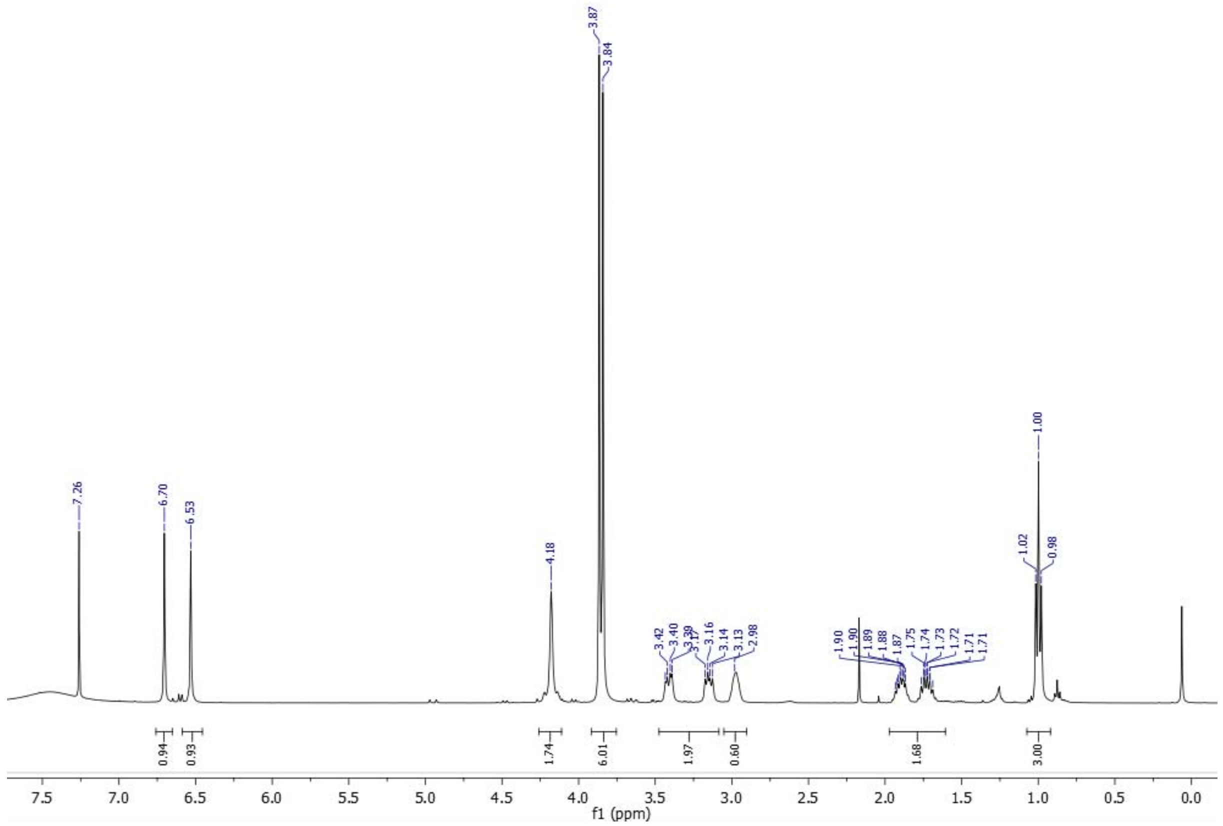

Figure S43: ^{1}H of 6,7-Dimethoxy-4-ethyl-1,2,3,4-tetrahydroisoquinoline (**S3**)

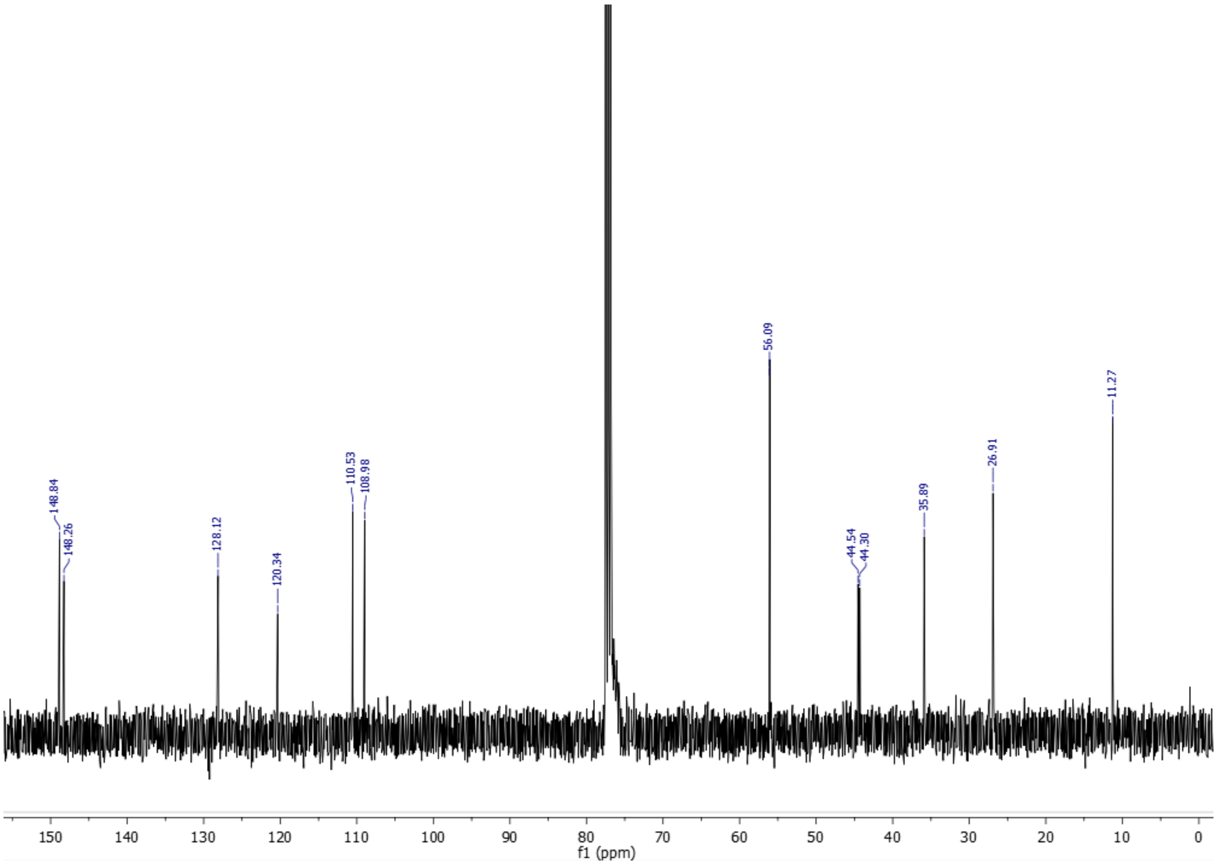

Figure S44: ^{13}C of 4-ethyl-6,7-dimethoxy-1,2,3,4-tetrahydroisoquinoline **(S3)**

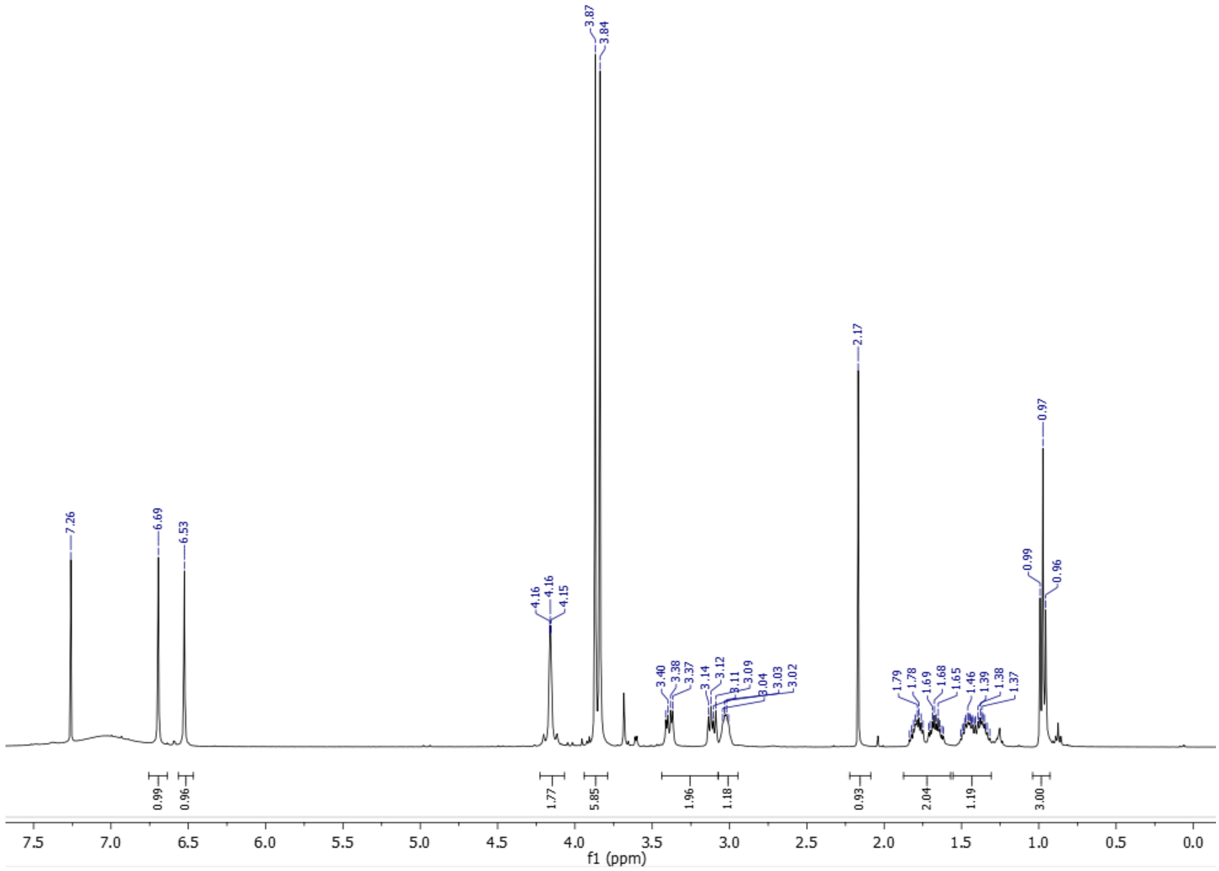

Figure S45: ^{1}H of 6,7-Dimethoxy-4-propyl-1,2,3,4-tetrahydroisoquinoline (**S4**)

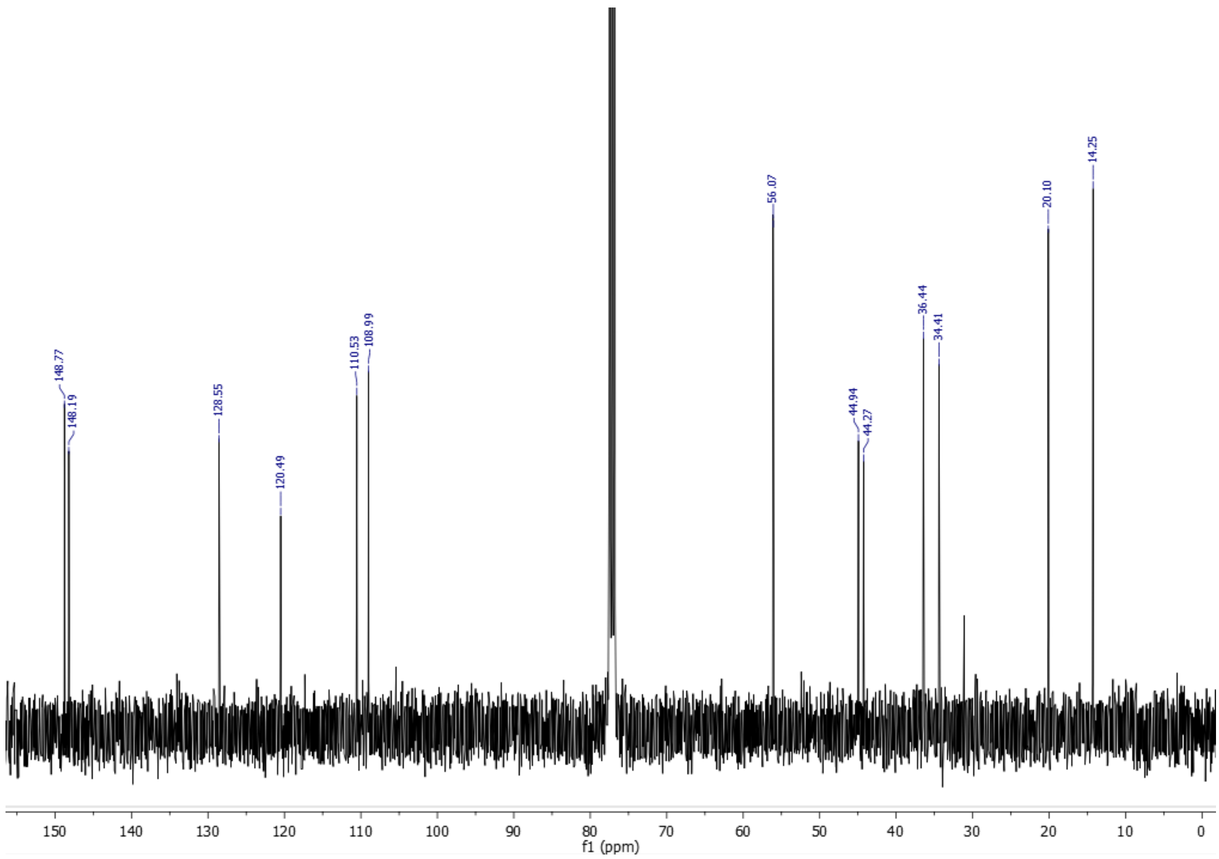

Figure S46: ^{13}C of 6,7-Dimethoxy-4-propyl-1,2,3,4-tetrahydroisoquinoline (**S4**)

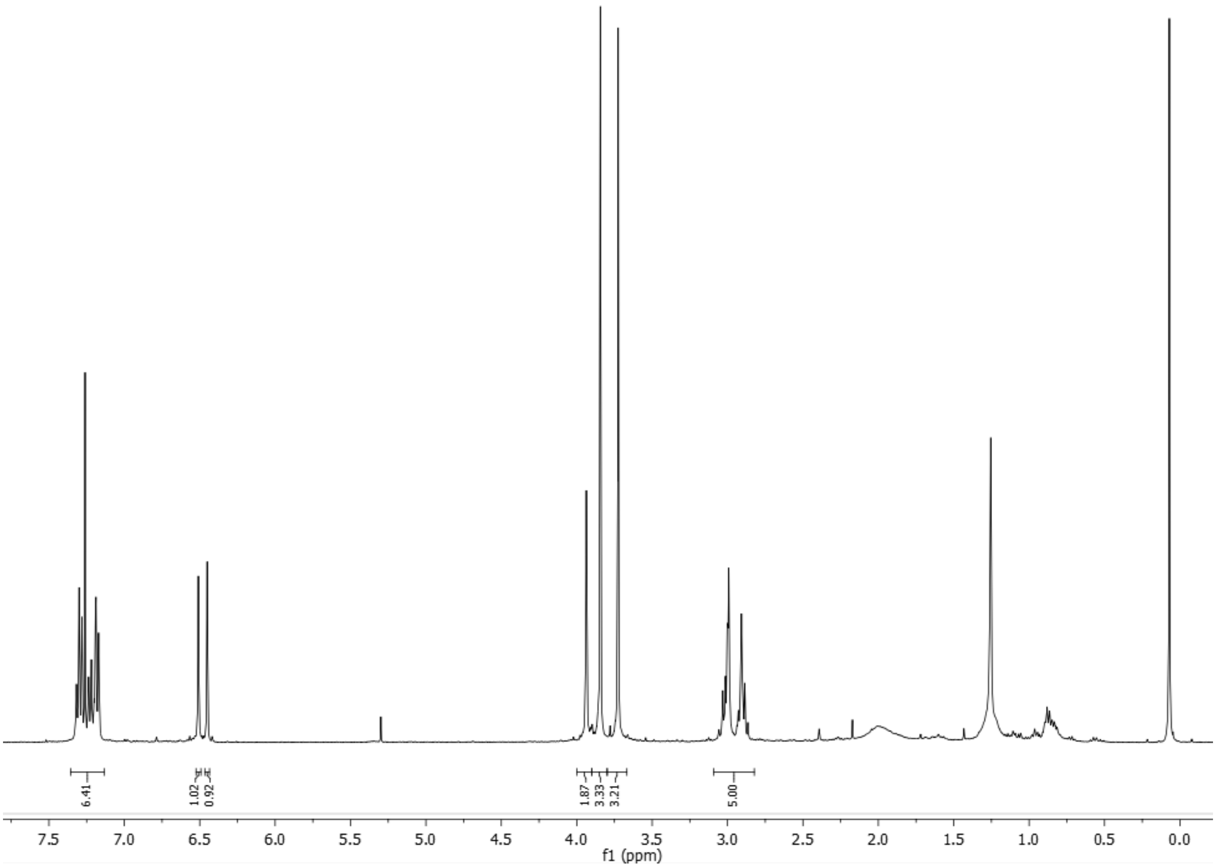

Figure S47: ^{1}H of 4-benzyl-6,7-dimethoxy-1,2,3,4-tetrahydroisoquinoline (**S5**)

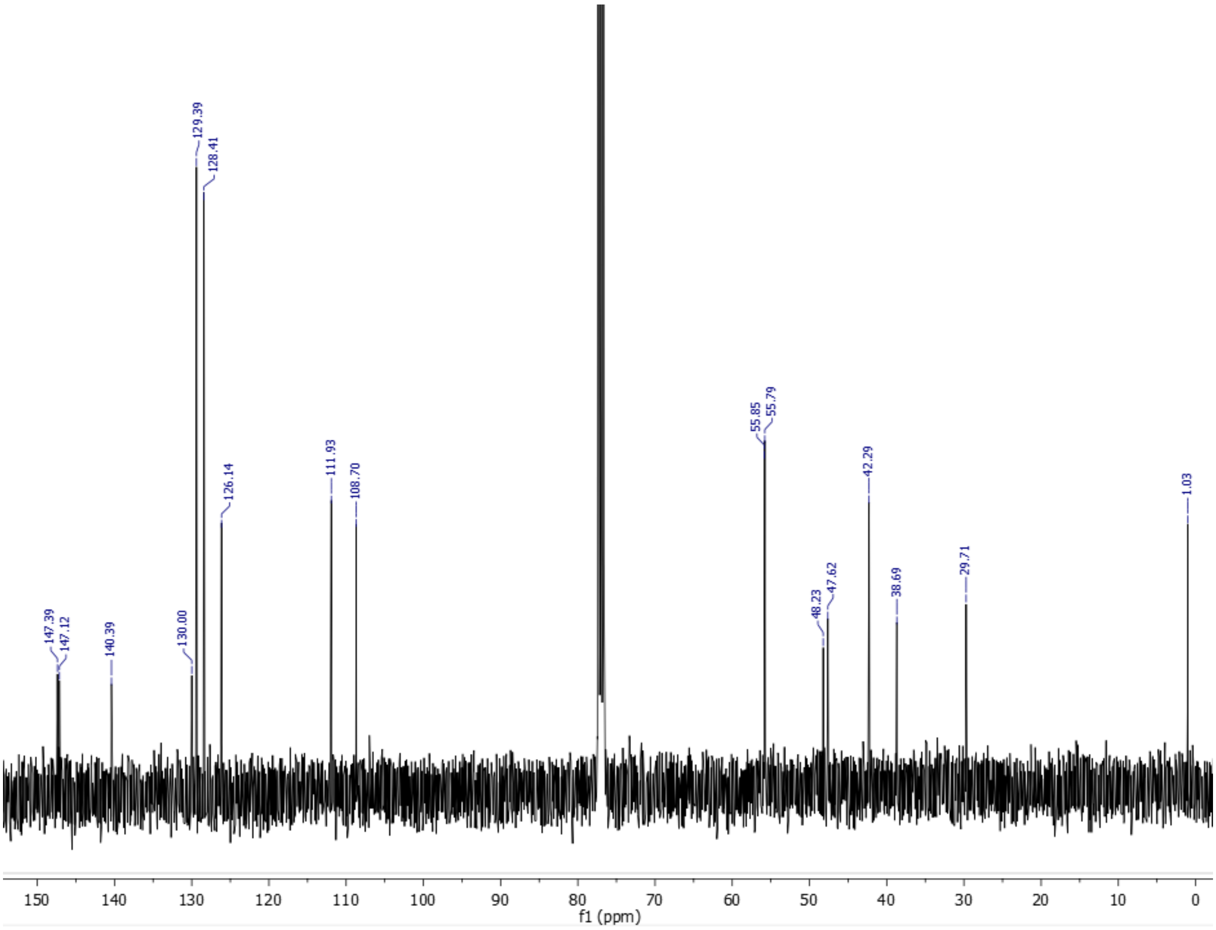

Figure S48: ^{13}C of 4-benzyl-6,7-dimethoxy-1,2,3,4-tetrahydroisoquinoline (**S5**)

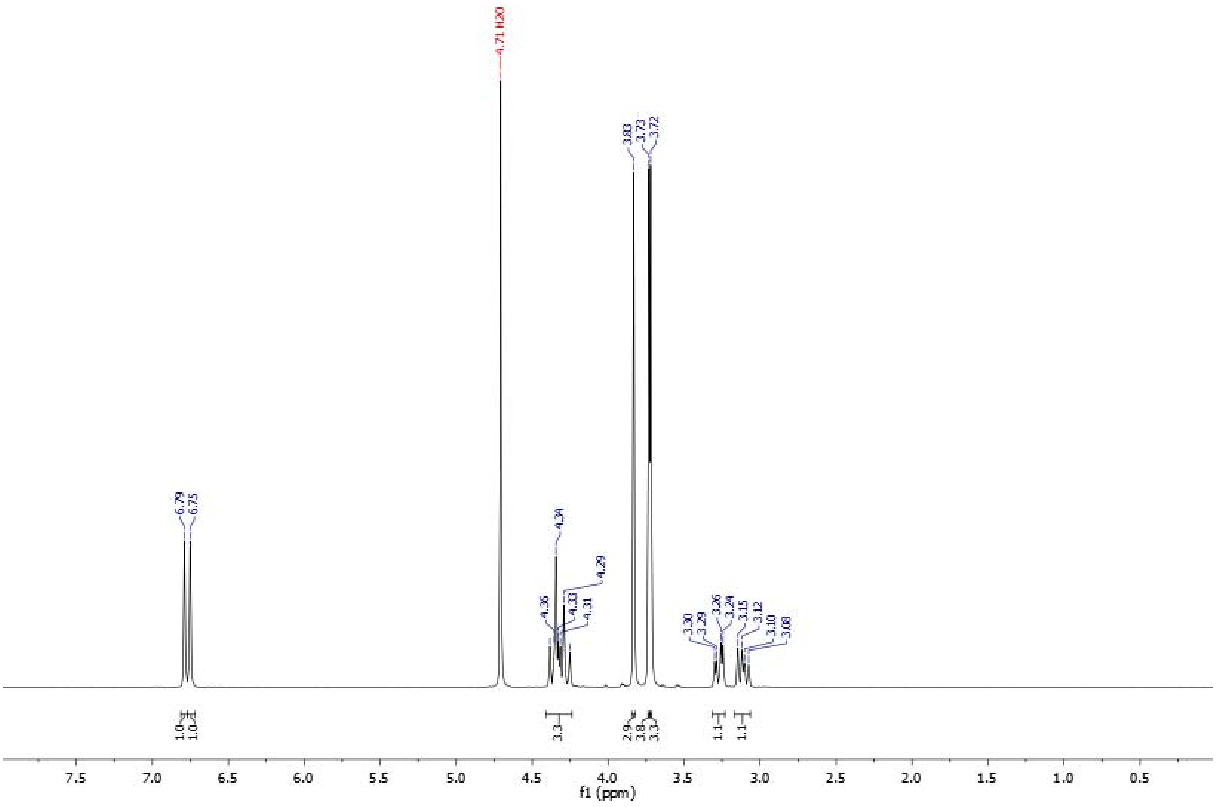

Figure S49: ^{1}H of methyl (S)-6,7-dimethoxy-1,2,3,4-tetrahydroisoquinoline-3-carboxylate(**S7**)

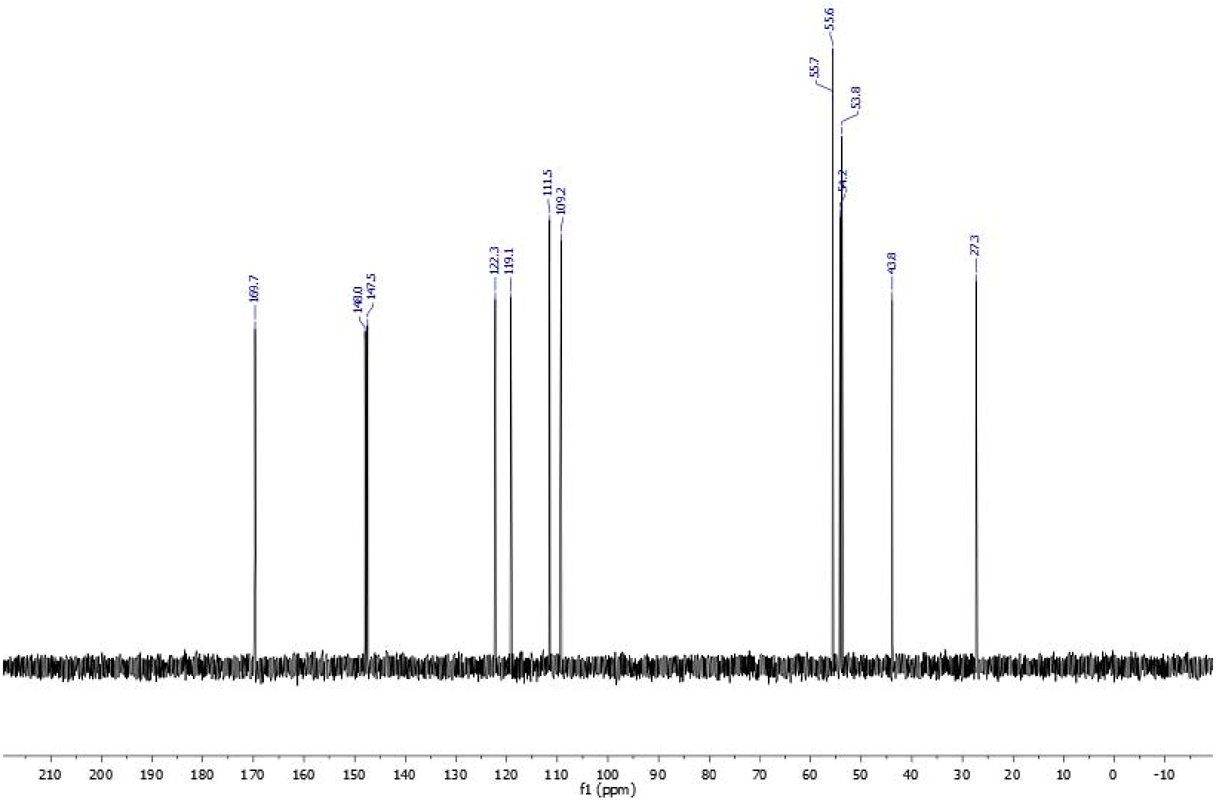

Figure S50: ^{13}C of methyl (S)-6,7-dimethoxy-1,2,3,4-tetrahydroisoquinoline-3-carboxylate(**S7)**

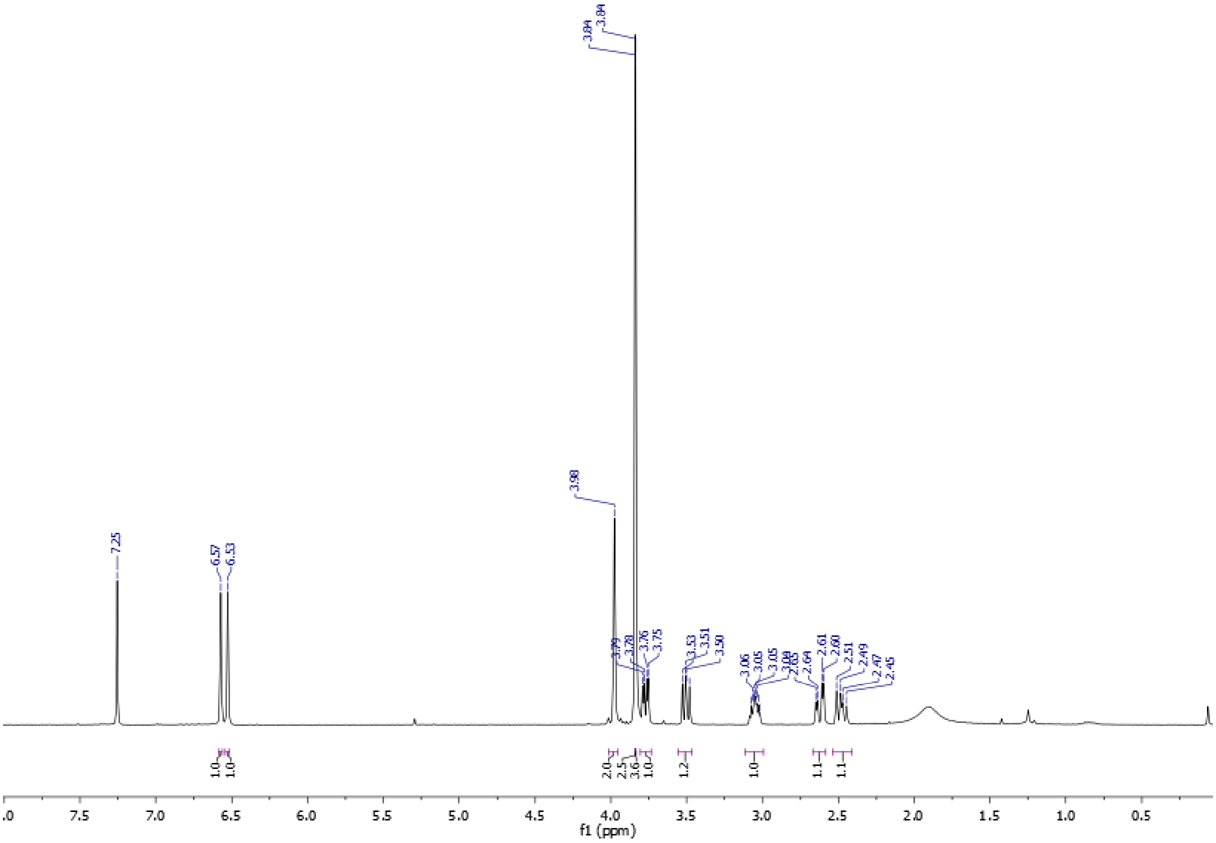

Figure S51: ^{1}H of (*S*)-6,7-Dimethoxy-3-hydroxymethyl-1,2,3,4-tetrahydroisoquinoline (**S8**)

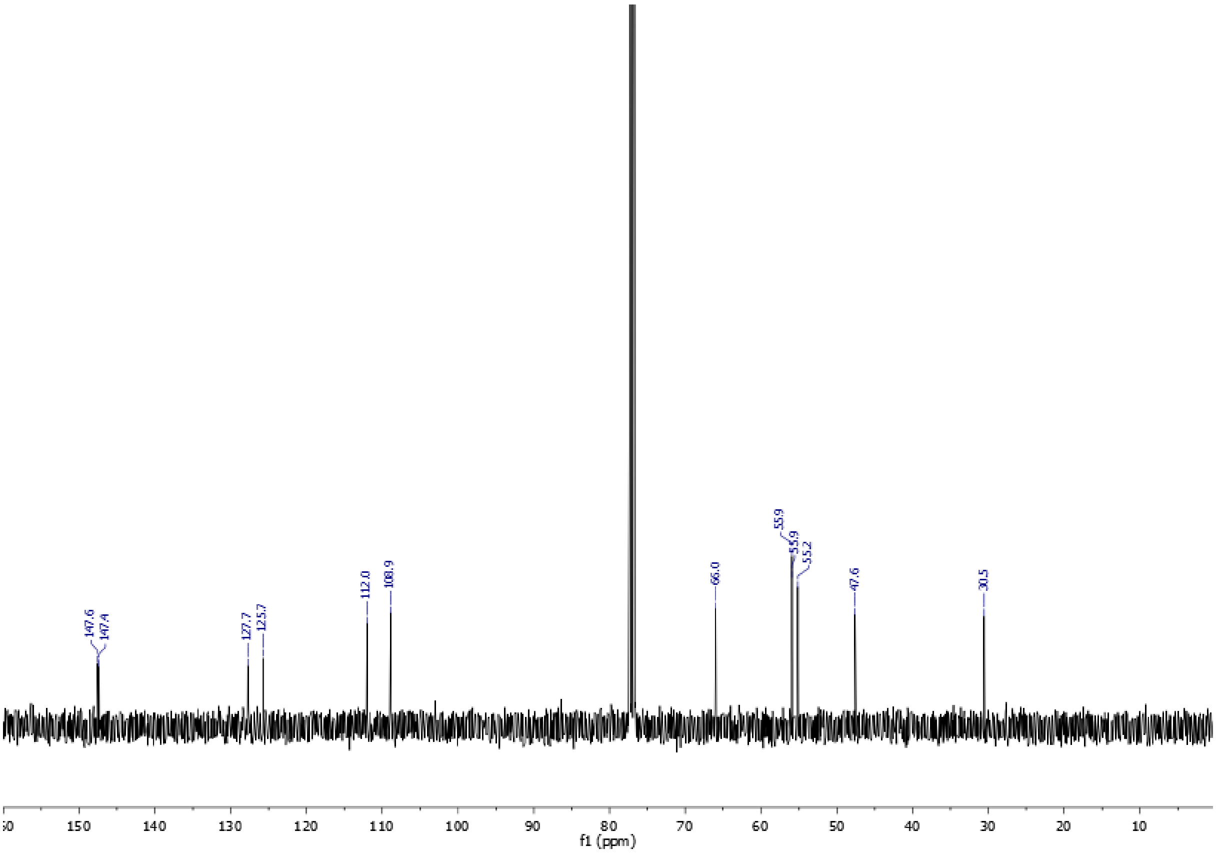

Figure S52: ^{13}C of (*S*)-6,7-Dimethoxy-3-hydroxymethyl-1,2,3,4-tetrahydroisoquinoline (**S8**)

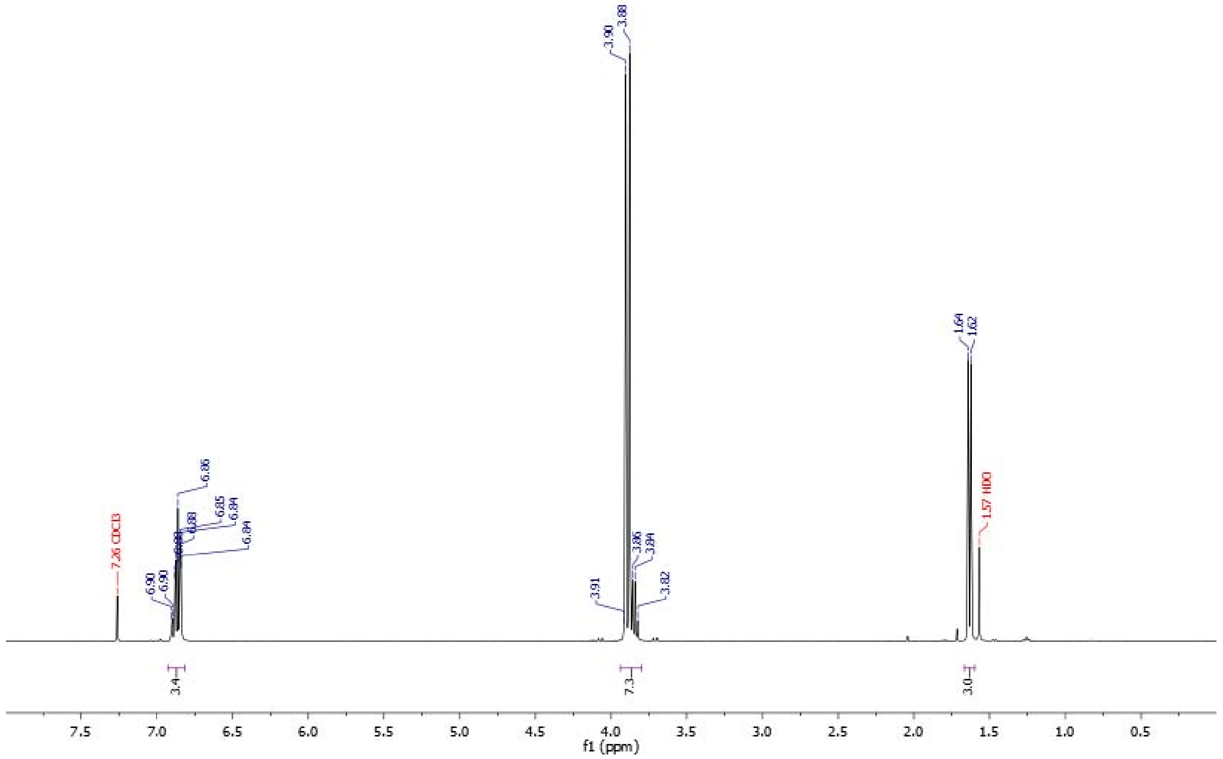

Figure S53: ^{1}H of 2-(3,4-Dimethoxyphenyl)propionitrile **(S16**)

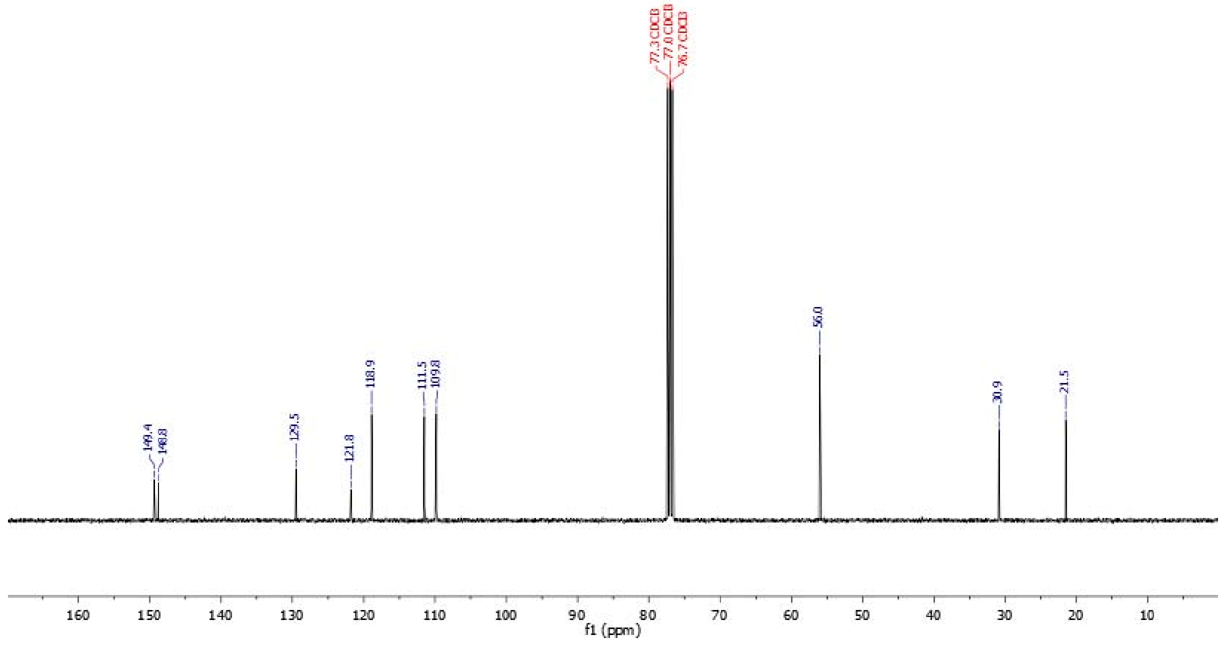

Figure S54: ^{13}C of (2-(3,4-Dimethoxyphenyl)propionitrile (**S16**)

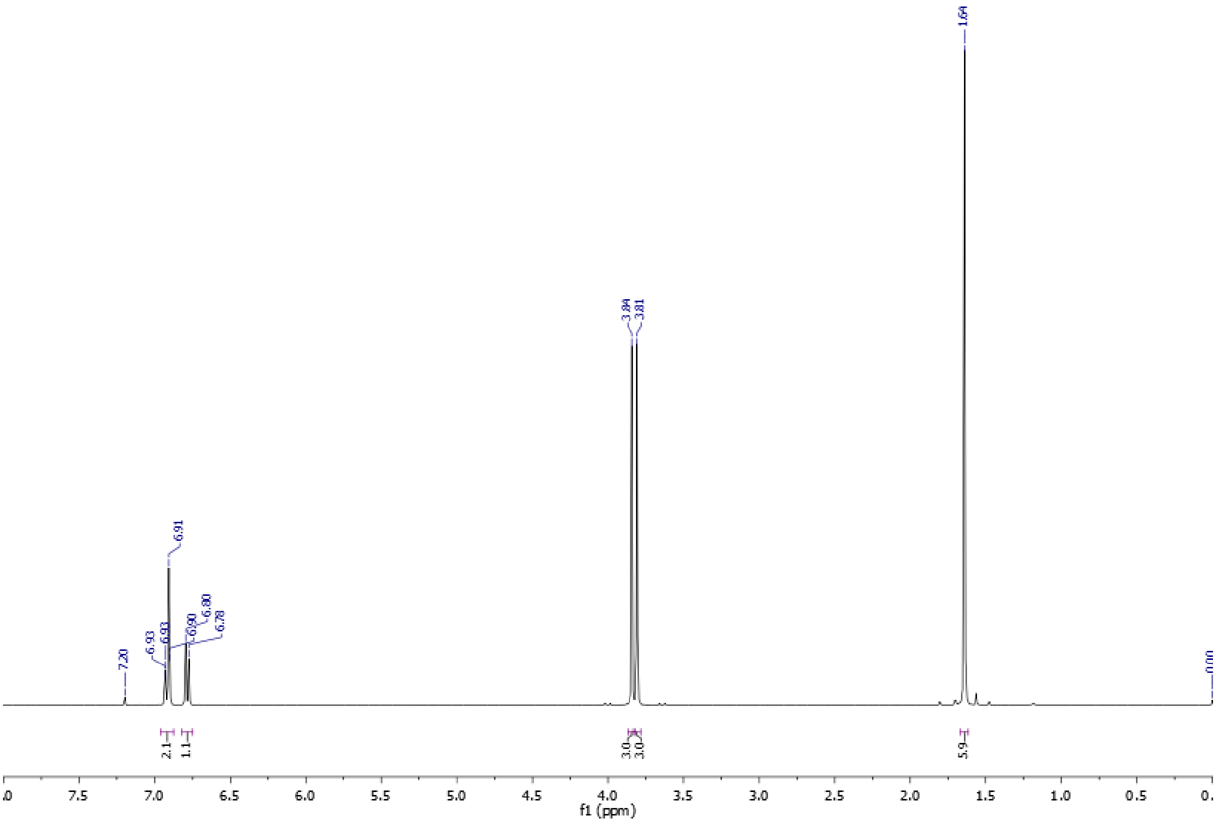

Figure S55: ^{1}H of 2-(3,4-dimethoxyphenyl)-2-methylpropionitrile (**S17**)

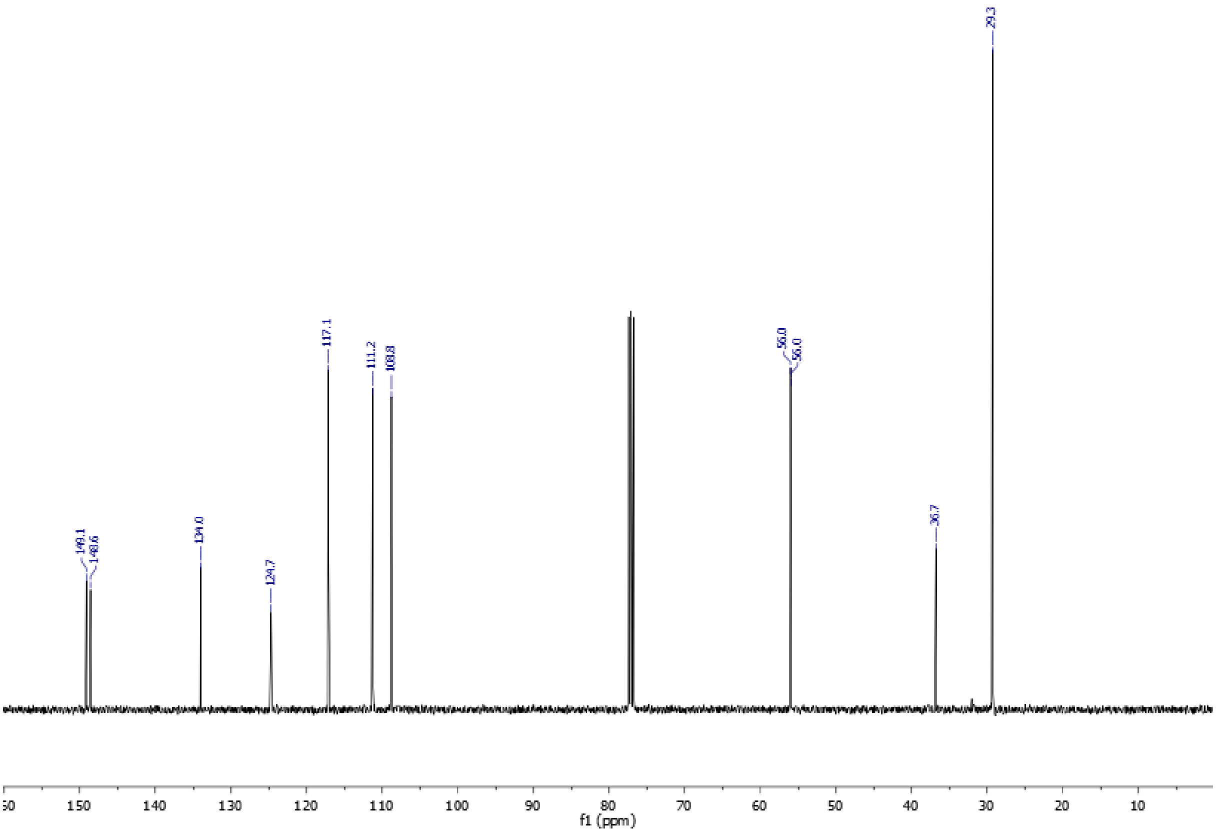

Figure S56: ^{13}C of 2-(3,4-dimethoxyphenyl)-2-methylpropionitrile (**S17**)

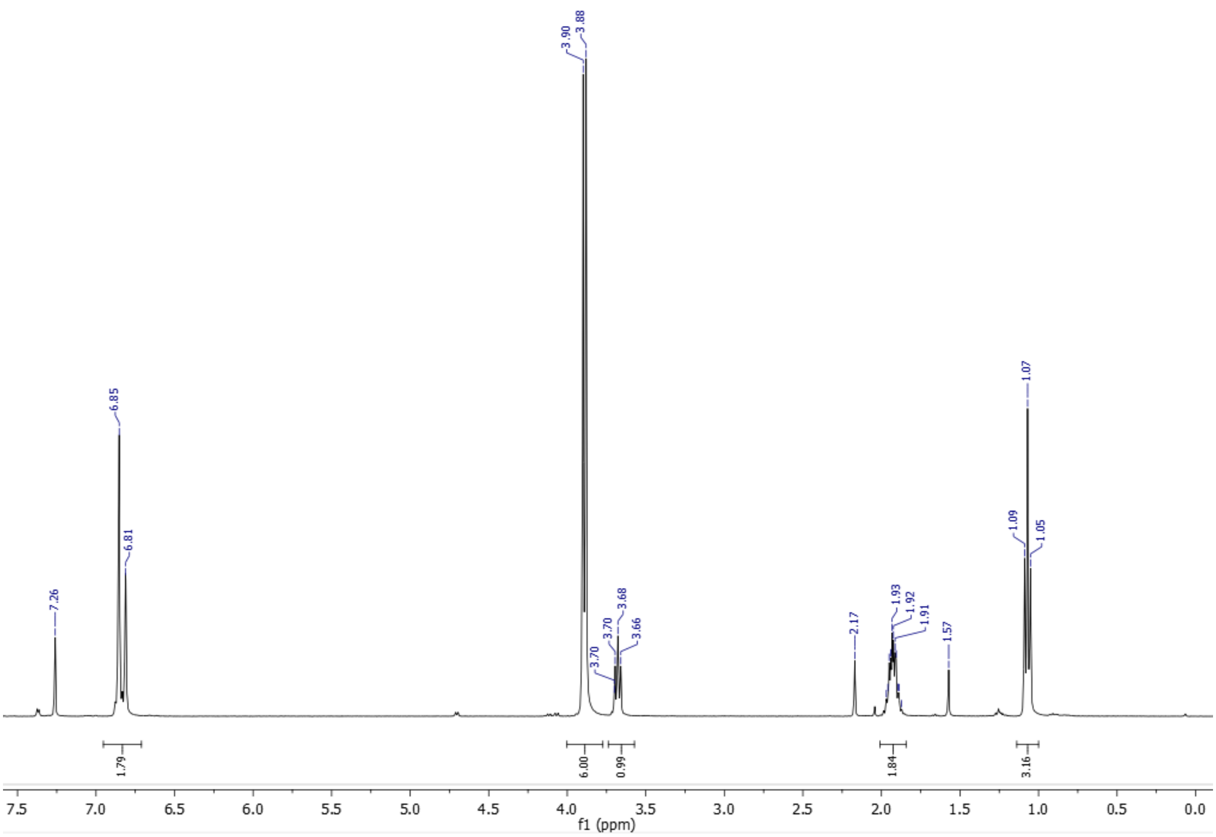

Figure S57: ^{1}H of 2-(3,4-Dimethoxyphenyl)butyronitrile (**S18**)

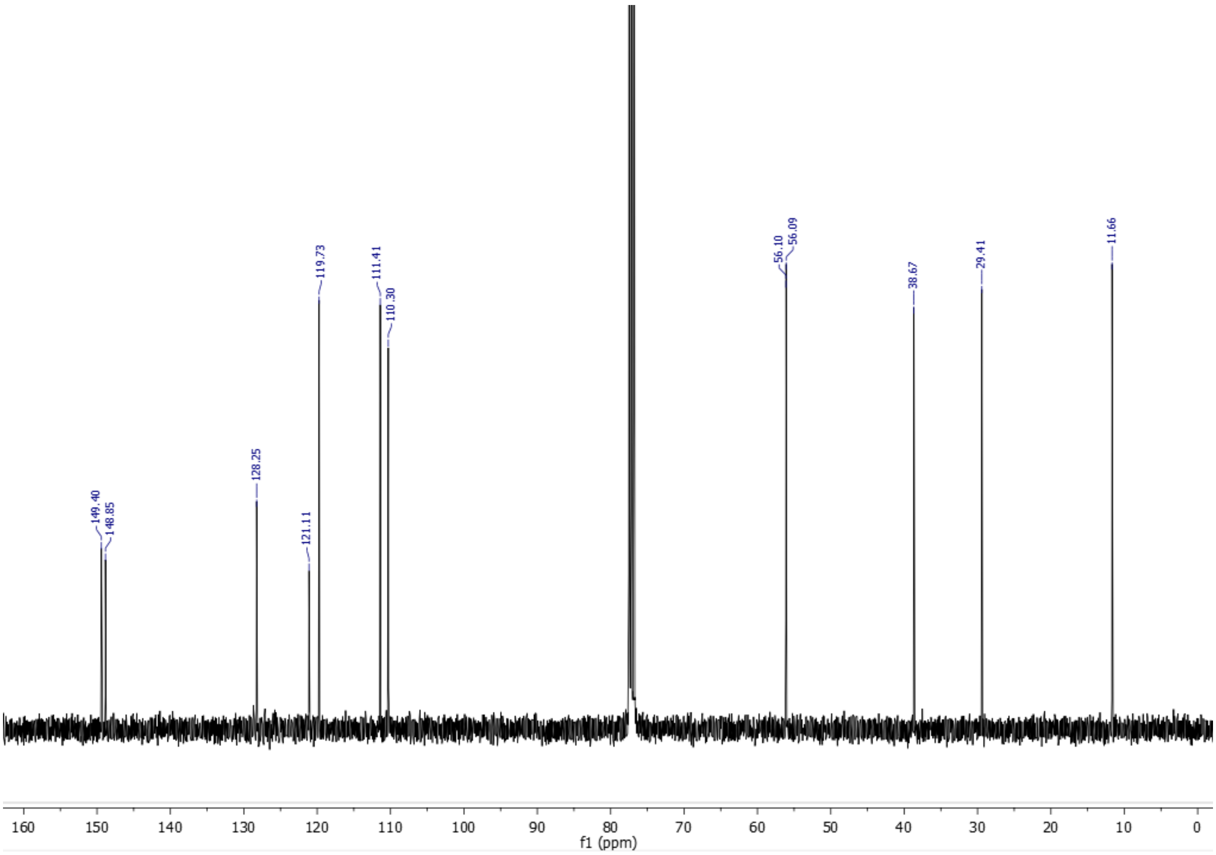

Figure S58: ^{13}C of 2-(3,4-Dimethoxyphenyl)butyronitrile (**S18**)

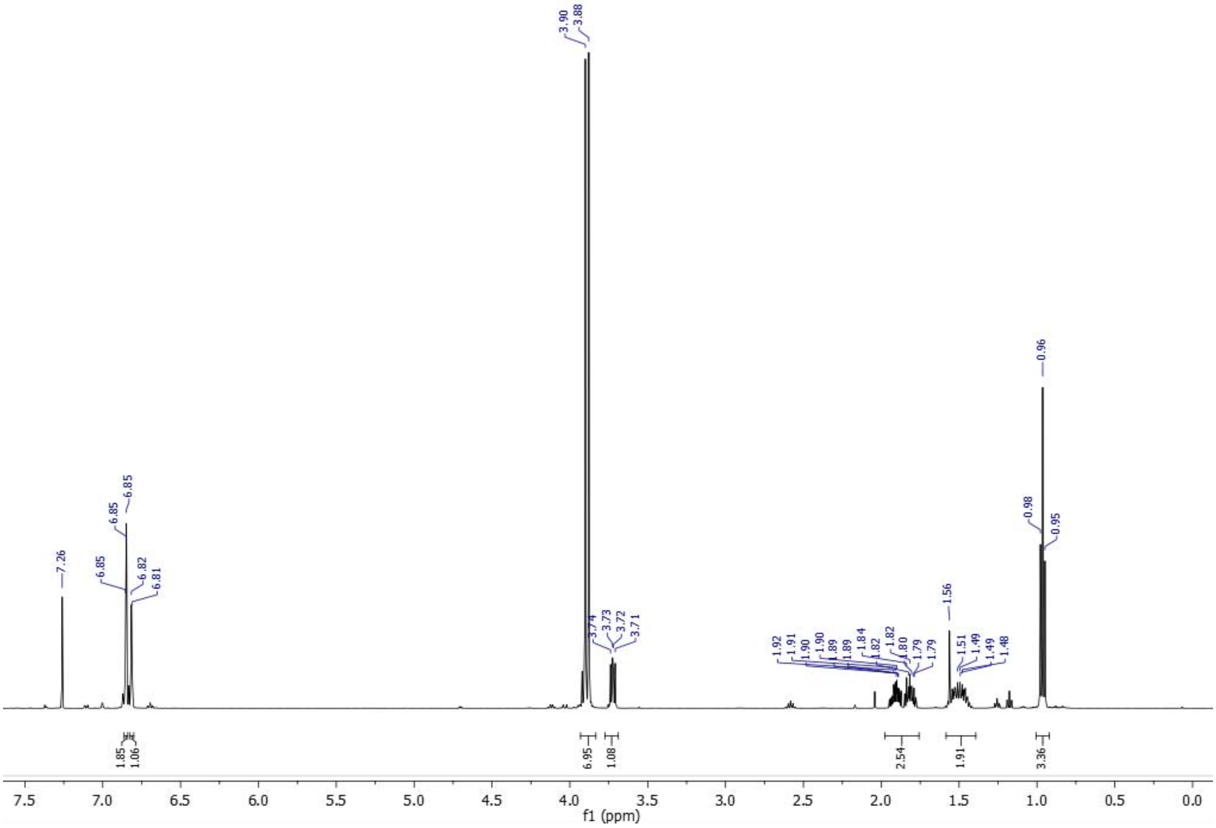

Figure S59: ^{1}H of 2-(3,4-Dimethoxyphenyl)pentanenitrile (**S19**)

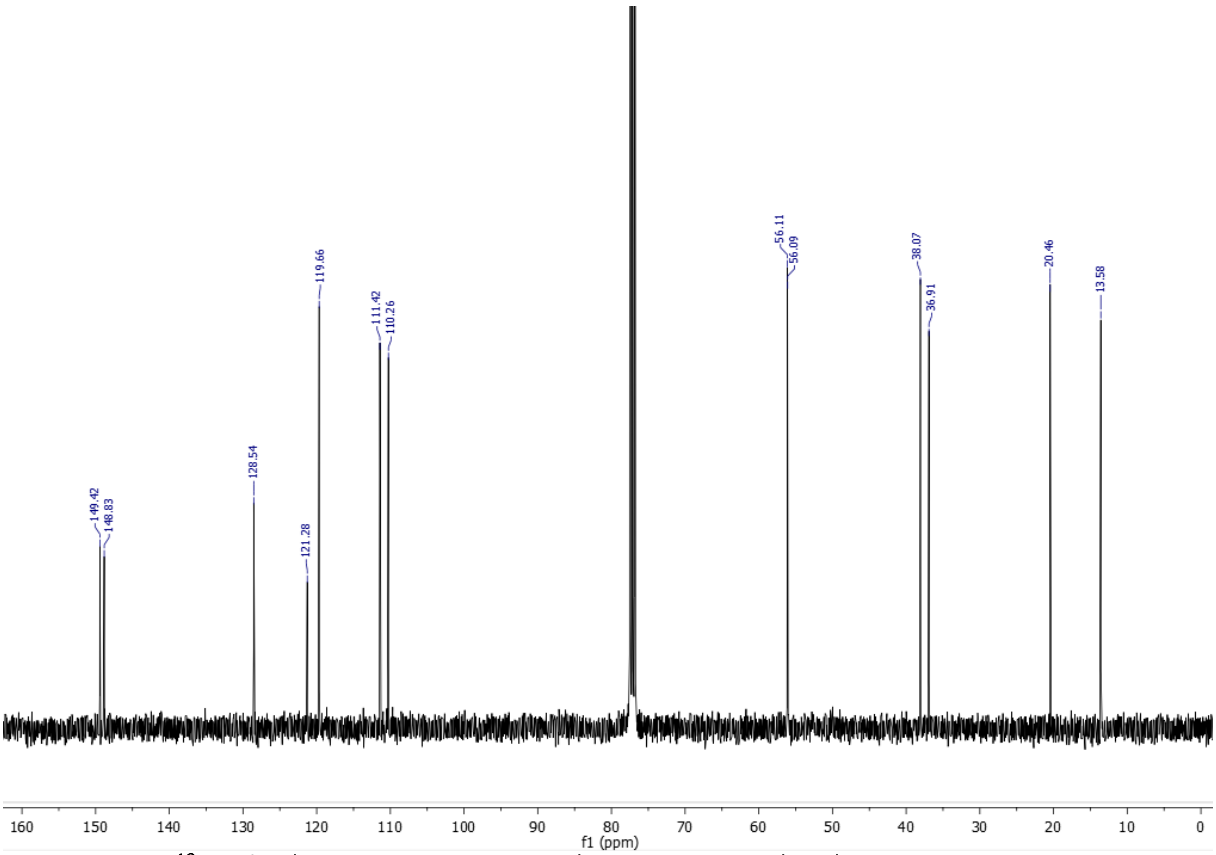

Figure S60: ^{13}C of 2-(3,4-Dimethoxyphenyl)pentanenitrile (**S19**)

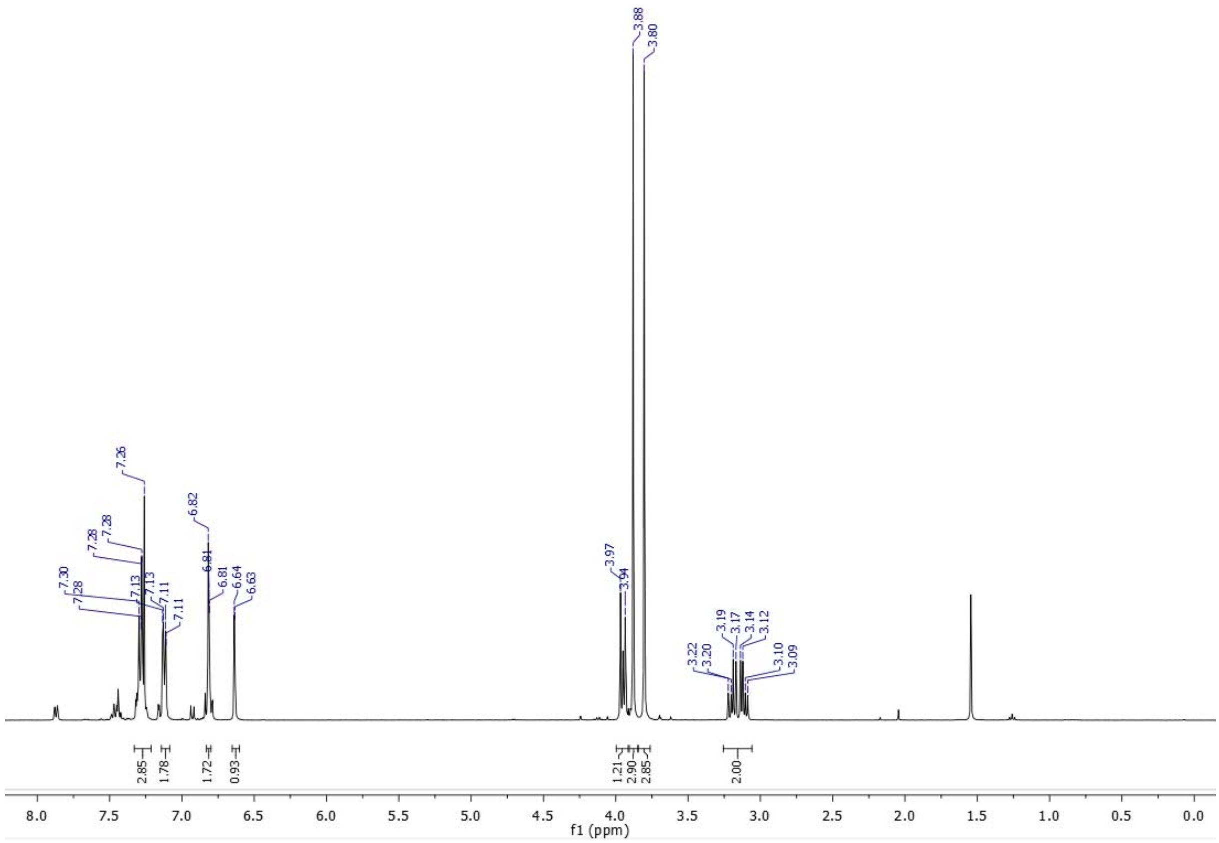

Figure S61: ^{1}H of 2-(3,4-dimethoxyphenyl)-3-phenylpropanenitrile (**S20**)

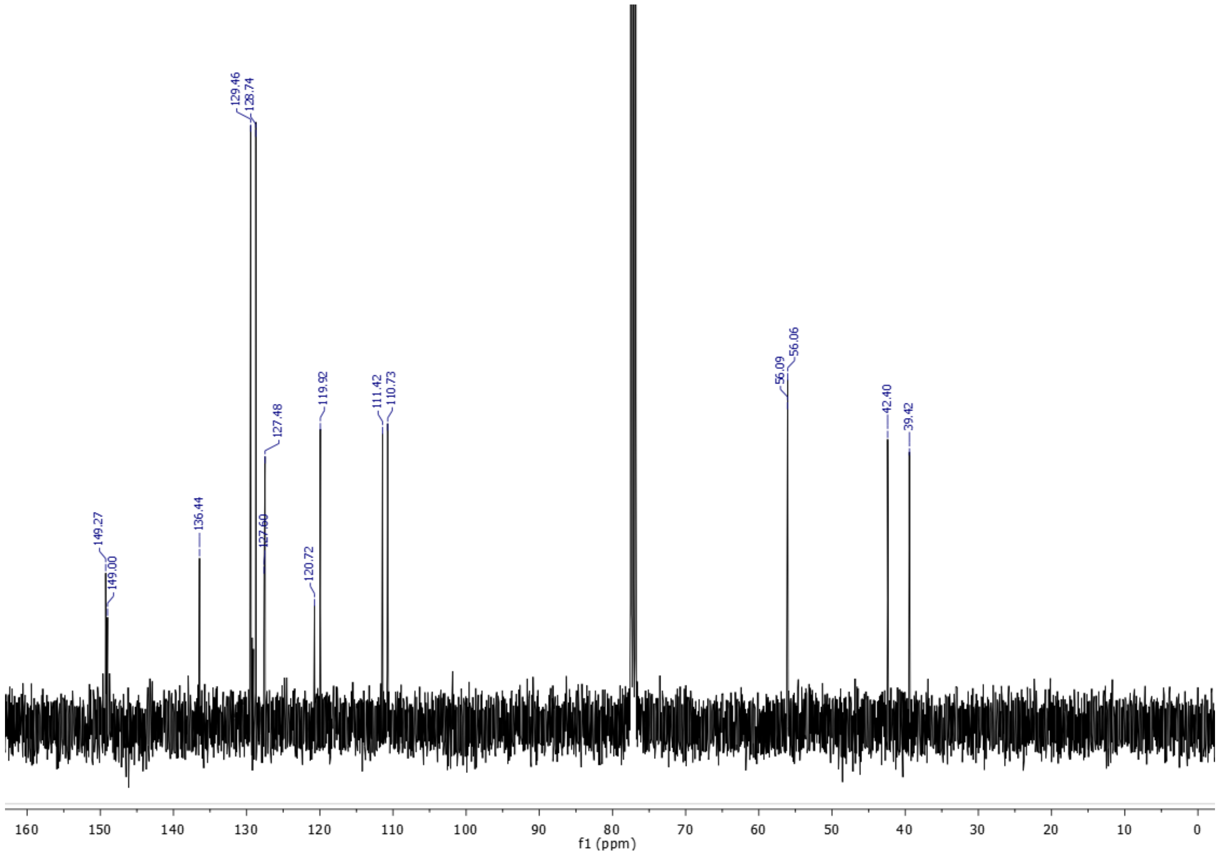

Figure S62: ^{13}C of 2-(3,4-dimethoxyphenyl)-3-phenylpropanenitrile (**S20**)
